# Rapid dissemination of host metabolism–manipulating genes via integrative and conjugative elements

**DOI:** 10.1073/pnas.2309263121

**Published:** 2024-03-08

**Authors:** Elena Colombi, Frederic Bertels, Guilhem Doulcier, Ellen McConnell, Tatyana Pichugina, Kee Hoon Sohn, Christina Straub, Honour C. McCann, Paul B. Rainey

**Affiliations:** ^a^School of Agriculture, Food and Ecosystem Sciences, Faculty of Science, The University of Melbourne, Parkville, VIC 3010, Australia; ^b^Department of Microbial Population Biology, Max Planck Institute for Evolutionary Biology, Plön 24306, Germany; ^c^Laboratoire Biophysique et Évolution, Institut Chemie Biologie Innovation, École Supérieure de Physique et de Chemie Industrielles de la Ville de Paris, Université Paris Science et Lettres, Centre National de al Reserche Scientifique, Paris 75005, France; ^d^Plant Immunity Research Center, Seoul National University, Seoul 08826, Republic of Korea; ^e^Department of Agricultural Biotechnology, Seoul National University, Seoul 08826, Republic of Korea; ^f^Research Institute of Agriculture and Life Sciences, Seoul National University, Seoul 08826, Republic of Korea; ^g^Plant Genomics and Breeding Institute, Seoul National University, Seoul 08826, Republic of Korea; ^h^Health and Environment, Institute of Environmental Science and Research, Auckland 1025, New Zealand; ^i^Division of Microbial Ecology, Center for Microbiology and Environmental Systems Science, University of Vienna, Vienna 1030, Austria; ^j^Plant Pathogen Evolution Research Group, Max Planck Institute for Biology, Tübingen 72076, Germany

**Keywords:** horizontal gene transfer, mobile elements, plant–microbe interactions, microbial evolution

## Abstract

Integrative and conjugative elements (ICEs) are self-transmissible mobile elements that transfer functional genetic units across broad phylogenetic distances. Accessory genes shuttled by ICEs can make significant contributions to bacterial fitness. Most ICEs characterized to date encode readily observable phenotypes contributing to symbiosis, pathogenicity, and antimicrobial resistance, yet the majority of ICEs carry genes of unknown function. Recent observations of rapid acquisition of ICEs in a pandemic lineage of *Pseudomonas syringae* pv. *actinidae* led to investigation of the structural and functional diversity of these elements. Fifty-three unique ICE types were identified across the *P. syringae* species complex. Together they form a distinct family of ICEs (PsICEs) that share a distant relationship to ICEs found in *Pseudomonas aeruginosa*. PsICEs are defined by conserved backbone genes punctuated by an array of accessory cargo genes, are highly recombinogenic, and display distinct evolutionary histories compared to their bacterial hosts. The most common cargo is a recently disseminated 16-kb mobile genetic element designated Tn*6212*. Deletion of Tn*6212* did not alter pathogen growth in planta, but mutants displayed fitness defects when grown on tricarboxylic acid (TCA) cycle intermediates. RNA-seq analysis of a set of nested deletion mutants showed that a Tn*6212*-encoded LysR regulator has global effects on chromosomal gene expression. We show that Tn*6212* responds to preferred carbon sources and manipulates bacterial metabolism to maximize growth.

## Significance

Integrative and conjugative elements (ICEs) are mobile genetic entities that can introduce genes conferring fitness advantages to their bacterial hosts. ICEs associated with *Pseudomonas syringae* plant pathogens were discovered in 2000 and have since been shown to be responsible for the movement of virulence and antimicrobial resistance genes. We identified previously undetected ICEs within the *P. syringae* species complex that define a new family. Tn*6212*, a mobile genetic element with metabolism-associated genes, has recently invaded the *P. syringae* species complex via ICEs. Tn*6212* carries a set of genes that reprogram metabolism in the bacterial cell to maximize growth. The manipulation of bacterial host cell metabolism by Tn*6212* can allow pathogens to rapidly capitalize on preferred carbon sources during niche colonization.

Mobile genetic elements, such as plasmids and integrative and conjugative elements (ICEs), can move functional genetic units over broad phylogenetic distances, mediating abrupt changes in niche preferences and even contributing to speciation (1, 2). Sequence analyses suggest that ICEs are the most abundant type of conjugative element in bacteria (3). ICEs are chromosomally integrated elements that are passively replicated as a part of the genome but are capable of horizontal transmission facilitated by their encoded excision and conjugation systems. During the process of conjugation, ICEs excise and form circular intermediates. A conjugative relaxase introduces a single-strand nick, and the ICE is then transferred to the recipient cell via a type IV conjugation apparatus. Site-specific integration of reconstituted double-stranded DNA occurs in the recipient cell (4–6). Genes encoding integration, excision, conjugation, and regulation are typically encoded within modules referred to as “backbone” genes (6). In addition to essential genes, ICEs carry variable sets of accessory or “cargo” genes that make contributions to both ICE and host cell fitness. These include genes with functions associated with biofilm formation, pathogenicity and symbiosis, bacteriocin synthesis, and antibiotic and heavy metal resistance (4, 5, 7).

*Pseudomonas syringae* is a model organism for the study of microbial evolution and plant–microbe interactions due to its ubiquity in both agricultural and nonagricultural areas. Different lineages of *P. syringae* are responsible for frequent outbreaks of disease in a variety of crop plants, and *P. syringae* can be found in association with wild plants, leaf litter, rivers, snowpack, and even clouds (8–11). *P. syringae* is more appropriately referred to as a species complex, comprising 13 divergent phylogroups (PGs) (12). Although *P. syringae* is among the most well-studied bacterial plant pathogens, only eight related ICEs have been described among 901 complete and draft genomes of members of the *P. syringae* species complex (13–16).

The emergence of a new lineage of *P. syringae* pv. *actinidae* (*Psa*) resulted in a global outbreak of bleeding canker disease on kiwifruit (*Actinidia* spp.), with severe consequences for agricultural production in Europe, Asia, New Zealand, Australia, and Chile (17). Population genomic analyses of *Psa* revealed that the global outbreak was caused by a pandemic sublineage that emerged from a more diverse population of *Psa*-3 (18). Separate introduction events of this clonal sublineage resulted in outbreaks in nearly all kiwifruit growing regions of the world. Initial genome comparisons showed the outbreak strains sampled from Italy, New Zealand, and Chile, which varied by very few single nucleotide polymorphisms (SNPs) across the core genome, independently acquired three divergent ~100-kb ICEs during their global journey. The three ICEs have syntenic backbones sharing ∼75% nucleotide identity and carry identical 16-kb regions flanked by short palindromic sequences. Although these 16-kb regions lack features typical of transposons, they were labeled Tn*6212* (19) [also referred to as “enolase regions” (15)] and predicted to be linked to virulence of the pandemic sublineage of *Psa*-3 (15, 19). After introduction of *Psa*-3 in New Zealand, where foliar copper sprays are frequently used to suppress infections, genomic surveillance revealed that *Psa*-3 acquired a diverse pool of ICEs conferring copper resistance (16).

Well-characterized ICEs carry accessory genes that confer strong phenotypes, such as pathogenicity, antimicrobial resistance, and nodulation/nitrogen fixation (6); however, many ICEs transport uncharacterized cargo. We sought to determine the distribution and evolutionary history of the only known family of ICEs in *P. syringae* and assess the prevalence of Tn*6212* and its contribution to bacterial host fitness. We identified a total of 207 ICEs present among six different PGs of the *P. syringae* species complex. This pool of ICEs is composed of 53 distinct ICEs. Hotspots of cargo gene exchange were observed within otherwise conserved ICE backbones. Although a diverse cargo of accessory genes was identified, Tn*6212* was the most common, present across 175 ICEs. We then sought to determine whether its carriage changes bacterial host phenotypes in plant-associated environments. We found that Tn*6212* alters bacterial host gene expression, conferring a fitness benefit during growth on tricarboxylic acid cycle intermediates. While research on plant–pathogen interactions often focuses on plant immunity, our results suggest that ICEs may play a role in adaptation of pathogens to the plant host environment by fine-tuning metabolism in response to host-derived cues.

## Results

### An Expanded Family of ICEs Is Circulating in the *P. syringae* Species Complex.

Bacterial whole genome sequences (updated to November 2017) and complete bacterial genomes (updated to July 2021) in the NCBI GenBank were interrogated using BLASTn (20) with ICEPsaCL1 (15), ICEPsaI10 (15), ICEPsaNZ13 (15), ICEPsyB728a (14), and ICEPph1302A (21) as queries (*SI Appendix*, Table S1) to identify related ICEs. This search resulted in a collection of 207 ICEs, collectively referred to as PsICEs (*SI Appendix*, Table S1). The 207 PsICEs (*SI Appendix*, Table S1) were found integrated in the 3′ end of two tRNA-Lys genes, 41% in *att-2* and 32% in the *att-1* site (in the remaining 27%, the contig was too short to infer the position of the tRNA-Lys). The integration leads to the formation of 52-bp direct repeats flanking the PsICEs (22) that allow delineation of the ICE sequence. The first integration site (*att-1*) is proximal to *clpB* (*Psa* NZ13 IYO_024910), and the second site (*att-2*) is proximal to *queC* (*Psa* NZ13 IYO_008010) (15, 22). ICEPs309-1 was integrated in a tRNA-Lys not adjacent to *clpB* or *queC* (*att*-3). Although BLASTn searches were not restricted to any bacterial species, only ICEs present in plant-associated *Pseudomonas* spp. [i.e., *P. syringae* species complex (11, 23)] were identified. PsICEs were harbored by diverse strains belonging to PGs 1, 2, 3, 4, 7, and 13 (*SI Appendix*, Fig. S1) (12). PsICEs in PG1 strains are overrepresented due to the availability of *Psa* genomes isolated from kiwifruit in China, South Korea, and Japan (18) (BioProject: PRJNA1018409). This constitutes a source of sampling-generated bias.

**Fig. 1.**
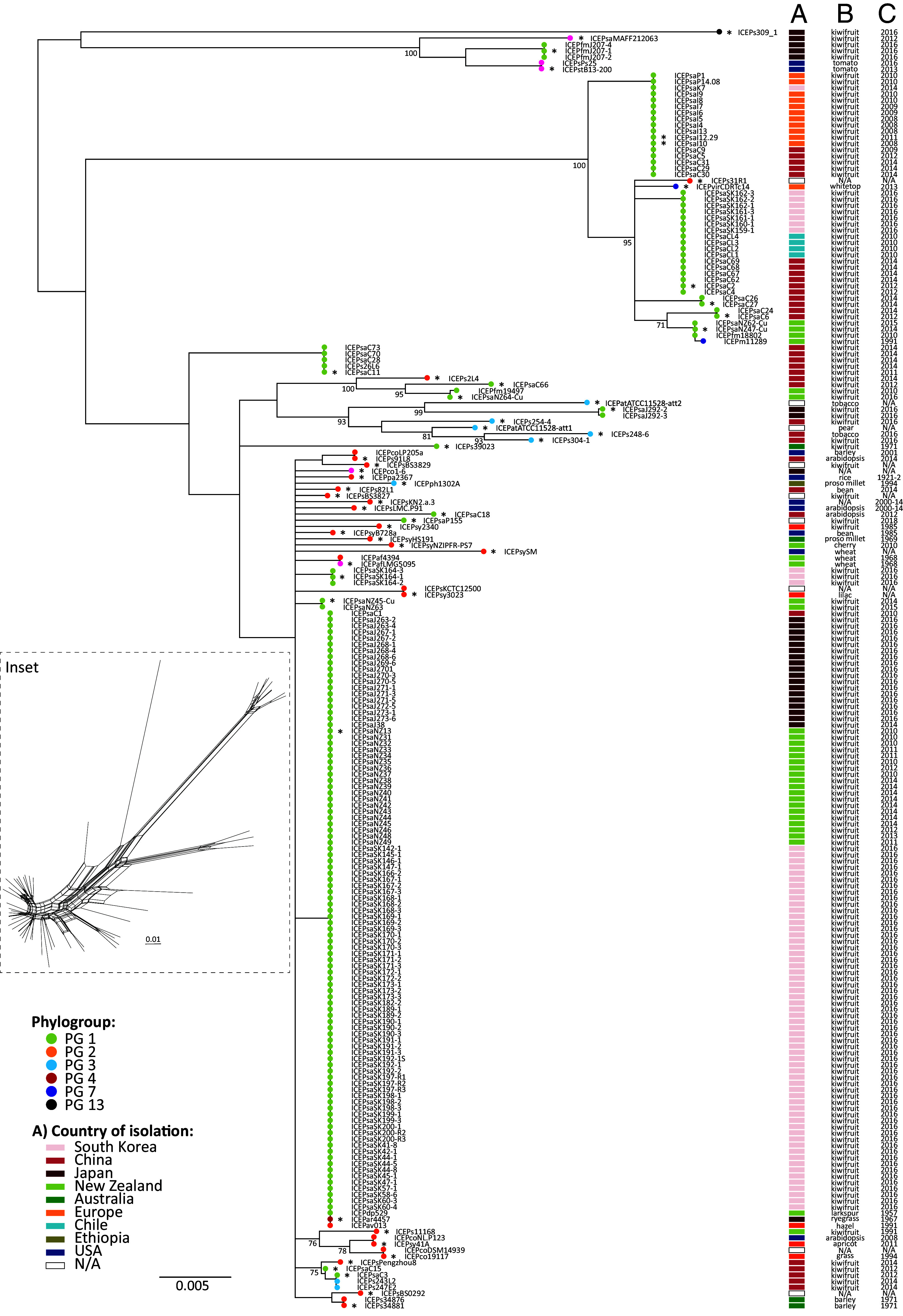
PsICEs are a large family of ICEs in the *P. syringae* species complex. An alignment of conserved positions in all PsICEs identified in the *P. syringae* species complex was generated using REALPHY. The 1,975 bp alignment was used to build a Neighbor Joining tree with 100 bootstrap replicates. The tree was rooted at midpoint. The *Inset* panel shows Neighbor-Net generated in SplitsTree using a concatenated alignment of backbone genes conserved in all 53 nonredundant ICEs. The scale bar indicates substitutions per site. Colors of terminal nodes indicate the phylogroup of the bacterial host genome harboring each PsICE; asterisks before PsICE names indicate nonredundant ICEs. Column *A* depicts the geographic location of the bacterial host; columns *B* and *C* show the plant host from which the bacterium was isolated and the year of isolation, respectively. Numbers at nodes represent bootstrap support values; only values >70 are shown. The scale bar indicates substitutions per site.

SNPs conserved across all 207 PsICEs were used to produce a phylogram (Fig. 1). Inter-ICE recombination (4, 7, 16) and the limited size of the aligned fragment (1,975 bp) mean that the resulting phylogram should be treated with caution and should not be considered a reliable representation of ICEs’ evolutionary history (Fig. 1, *Inset*). It is notable, however, that the largest cluster includes ICEs isolated from kiwifruit in New Zealand, China, Japan, and South Korea from 2010 onward. Interestingly, ICEs from divergent *P. syringae* isolated from larkspur in 1957 (ICEPdp529), hazel in 1991 (ICEPav013), and ryegrass in 1967 (ICEPar4457) fall into the same cluster. This cluster includes the canonical ICEPsaNZ13. ICEs within this cluster that share the same set of accessory genes as ICEPsaNZ13 (see below) are henceforward referred to as ICEPsaNZ13-like elements.

Consistent with their capacity for horizontal transfer, PsICE distribution is incongruent with host strain phylogeny (*SI Appendix*, Fig. S1). Overall, PsICEs show no correlation with year, plant, or geographic location of bacterial host (Fig. 1). For example, ICEPsaNZ13-like elements are present in bacterial isolates distributed across multiple PGs, including PG1 (pandemic sublineage *Psa*-3 strains), PG2 (*P. syringae* pv. *avellanae* ISPaVe013), and PG4 (*P. coronafaciens* pv. *atropurpurea* ICMP4457). ICEPsaC15-like and ICEPsaC3-like elements are highly similar and have been isolated in both *P. syringae* PG1 (*Psa*) and *P. savastanoi* (PG3) host strains found in association with kiwifruit in China. Conversely, distinctly different ICEs are present in otherwise closely related host strains isolated from the same plant host in the same geographical area. ICEs from different years sometimes cluster together, for example, the aforementioned ICEPdp529 (1957) and ICEPar4457 (1967) group with ICEPsaNZ13-like elements isolated in 2016.

To identify ICEs in *Pseudomonas* genomes other than those of *P. syringae*, the NCBI GenBank repository (excluding *P. syringae*) was interrogated in April 2017 with the ICEPsaNZ13 DEAD-box helicase protein sequence, the most highly conserved gene among all PsICEs. Forty-four ICEs carrying DEAD-box helicases were identified in 41 *Pseudomonas* genomes, 82% of which were *Pseudomonas aeruginosa* (*SI Appendix*, Table S2). All ICEs were integrated in one of the two *att* sites described for PsICEs, with the exception of an ICE in *P. aeruginosa* PA38182, which did not harbor recognizable *att* sites. Thirty-three ICEs were part of the pKLC102/PAGI-2 family of ICEs (24) (*SI Appendix*, Table S2). Although the pKLC102/PAGI-2 and PsICEs form clearly distinct families (*SI Appendix*, Fig. S2), most of the conserved ICE life cycle genes are shared among the two families and are syntenic (*SI Appendix*, Fig. S2). This finding thus places the PsICEs in a broader context of ICEs found in the gammaproteobacteria (25).

### Conservation of Genetic Organization Despite Frequent Inter-ICE Recombination.

ICE sequences are typically composed of conserved backbone genes (involved in ICE life cycle function) and variable accessory (or cargo) genes. Identification of the PsICE backbone was guided by identification of the set of genes present among at least 93% of all ICEs using ROARY (26). The backbone consists of a set of 62 genes (~56 kb) predicted to be involved in ICE maintenance, regulation and transmission, as well as a number of conserved hypothetical proteins (*SI Appendix*, Fig. S3 and Table S3). Homologs of these genes in other ICEs have been shown to encode a conjugative pilus (*pil*), ICE transfer (*tra*), partitioning (*par*) and integration (*int*) functions (6). The relaxed criterion chosen here (present in at least 93% ICEs) reflects the possibility of misassembly or gene deletion events (*SI Appendix*, Fig. S3). PsICE backbone genes are syntenic, with an average nucleotide identity across all genes of 86.4%. Average nucleotide identity varies between 94.1% for the gene encoding the DEAD-box helicase and 74.8% for a hypothetical protein-encoding gene (backbone gene #27). When distantly related PsICEs are compared (e.g., ICEPsaNZ13 vs. ICEPsaI10 and ICEPsaNZ13 vs. ICEPs309-1), the pairwise identity of the DEAD-box helicase gene decreases to 92.7% and to 70.3%, respectively (*SI Appendix*, Fig. S3).

Despite conservation of genes required for core ICE function and mobility, signatures of recombination are evident among ICEs. Backbone gene trees display phylogenetic incongruity (ILD tests, *P* of type I error = 0.01) (27). ClonalFrameML (28) detects several recombination events in an alignment of concatenated backbone genes, and Neighbor-net (29) produces a highly reticulated network with a statistically significant Phi test for recombination (*P* < 0.0001) (Fig. 1, *Inset* and *SI Appendix*, Fig. S4) (30). Finally, an alignment-free method of sequence comparison (31) indicates each PsICE is a chimera of other PsICEs, with short stretches of sequence displaying no homology to any known PsICE (*SI Appendix*, Table S4). Thus, inter-ICE recombination is rampant, has shaped PsICE evolution and diversity, and obscured evolutionary history.

### Variable Cargo Genes Are Present in PsICE Insertion Hotspots.

**Fig. 2.**
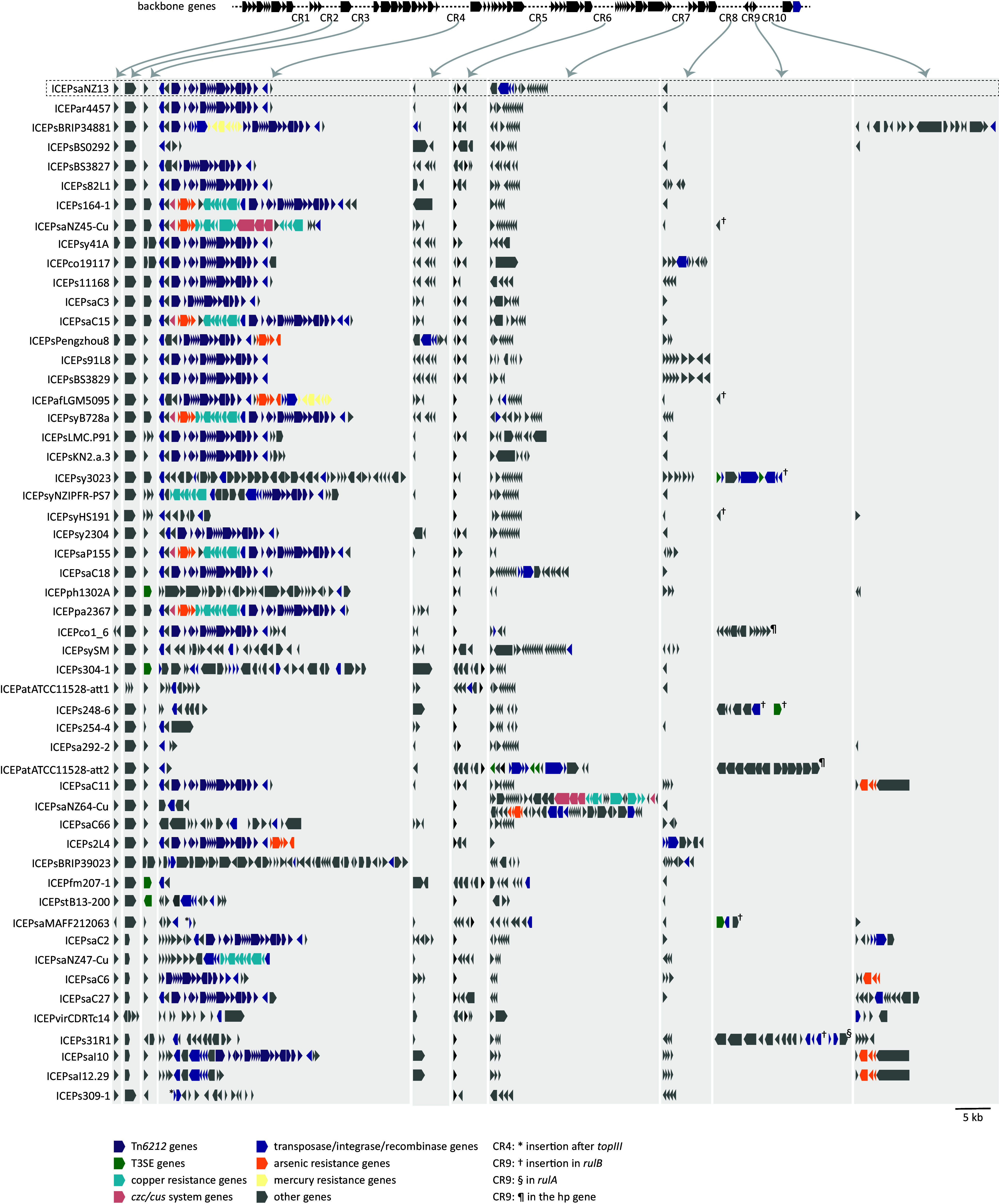
Genetic organization of PsICEs and hotspots of cargo gene integration. The gene content of the CR is highlighted with gray background; arrows indicate their position on the backbone. The distance between contiguous backbone genes varies accordingly to the content of the CR. ICE PsaNZ13 is highlighted with a rectangular box.

A subset of 53 nonredundant PsICEs was identified from the initial 207 PsICEs based on their position in the Neighbor Joining tree and on differences in gene content within clusters (*SI Appendix*, Table S1 and Fig. 1). This set of nonredundant PsICEs was used for all subsequent analyses. Comparison of the 53 nonredundant PsICEs reveals that the backbone serves as a scaffold for variation introduced at ten specific regions, referred to as cargo regions (CR). The CRs are found in intergenic positions, with the exception of CR9, an integration hotspot likely driven by the presence of *rulAB* (genes involved in ultraviolet tolerance and SOS response) (32) (Fig. 2). The integration of genes in CR9 results in the partial deletion of *rulA* or *rulB.* EGGNOG functional prediction (33) of the complete set of cargo genes shows that 70% have no predicted function. Some cargo genes are notably abundant: Tn*6212* is integrated into CR4 in 30 of the 53 nonredundant PsICEs. Imperfect direct repeats found at the extremities of Tn*6212* (19) may constitute sequences recognized by the XerC site-specific tyrosine integrase. Clusters of heavy-metal resistance genes integrated in CR4, alone, or in tandem with Tn*6212*, are surrounded by the same repeats. It thus seems possible that these elements specifically target ICE sequences, as was previously shown for integrative and mobilizable elements targeting loci in ICEs of the Tn*5252* superfamily present in *Streptococcus* genomes (34). Arsenic resistance genes are present in 13 nonredundant PsICEs; copper (and cadmium) resistance genes are found in 8, and two ICEs (ICEPaf4394 and Ps34881) harbor a ~7-kb transposon encoding mercury resistance. In contrast, PsICEs harbor few type 3 secretion system (T3SS) effectors (T3SEs): *hopAR1* (formerly *avrPphB*) is present in ICEPph1302A (21), ICEPs304-1 and ICEPfm207, ICEPatATCC11528-att2 carries *hopF2*, *hopO1-1*, *hopT1-1*, ICEPsaMAFF212036 harbors *hopAU1*, and ICEPs248-6 captured *avrRmp1* in the *rulAB* hotspot.

### Tn*6212* Enhances the Growth of *Psa* NZ13 on TCA Cycle Intermediates.

The high frequency and nucleotide identity of Tn*6212* across otherwise divergent PsICEs suggest that the element has recently spread and may confer a fitness advantage in plant-associated bacteria. Tn*6212* is a ~16-kb mobile genetic element first described as a tyrosine recombinase transposon (19) that consists of 20 genes, of which seven are predicted to encode hypothetical proteins (*SI Appendix*, Table S5). While full-length Tn*6212* is the most common cargo element, eight PsICEs carry only subsets of Tn*6212* (*SI Appendix*, Fig. S5). Although PsICE backbones often share low levels of pairwise identity, the full-length Tn*6212* elements share over 99% pairwise nucleotide identity.

The genes encoded by Tn*6212* are not obviously associated with pathogen virulence or antibacterial resistance. However, Tn*6212* encodes genes implicated in metabolism, including a transporter of dicarboxylic acids (DctT) predicted to import TCA cycle intermediates, and enolase, which encodes the penultimate step of glycolysis. *P. syringae* is known to use reverse carbon catabolite repression and prefers organic acids as carbon sources (35). The presence of a T3SS-targeting signal at the *N* terminus of DctT led to the hypothesis that DctT might be exported via T3SS into plant cells to deprive the plant of C_4_ sugars (15). To determine whether DctT is exported, the *dctT* promoter and its putative T3SS-targeting signal (1 to 52 aa) was fused to the C-terminal sequence of *avrRpt2* (pMT1), a T3SE recognized by *Arabidopsis thaliana* Col-0 (36). A second construct (pMT2) included the promoter and full-length *dctT*. Both *dctT:avrRpt2* constructs were confirmed as functional using transient expression via agroinfiltration into *Nicotiana benthamiana* (*SI Appendix*, Fig. S6). After introduction into *Psa* NZ13 and *Psa* NZ13Δ*hrcC,* which lacks a functional T3SS (37), strains were inoculated into *A. thaliana*, and plants were monitored for AvrRpt2 recognition via ion leakage assays and development of a hypersensitive response (HR) (*SI Appendix*, Fig. S6). These experiments showed that DctT was not exported via the T3SS, or via other means, by *Psa* NZ13. We then investigated whether carriage of Tn*6212* was involved in bacterial growth on plant hosts: *Psa* NZ13ΔTn*6212* was not significantly impaired in growth compared to the wild type after flood inoculation of *Actinidia chinensis* var. *chinensis* Hort16A (*SI Appendix*, Fig. S7).

**Fig. 3.**
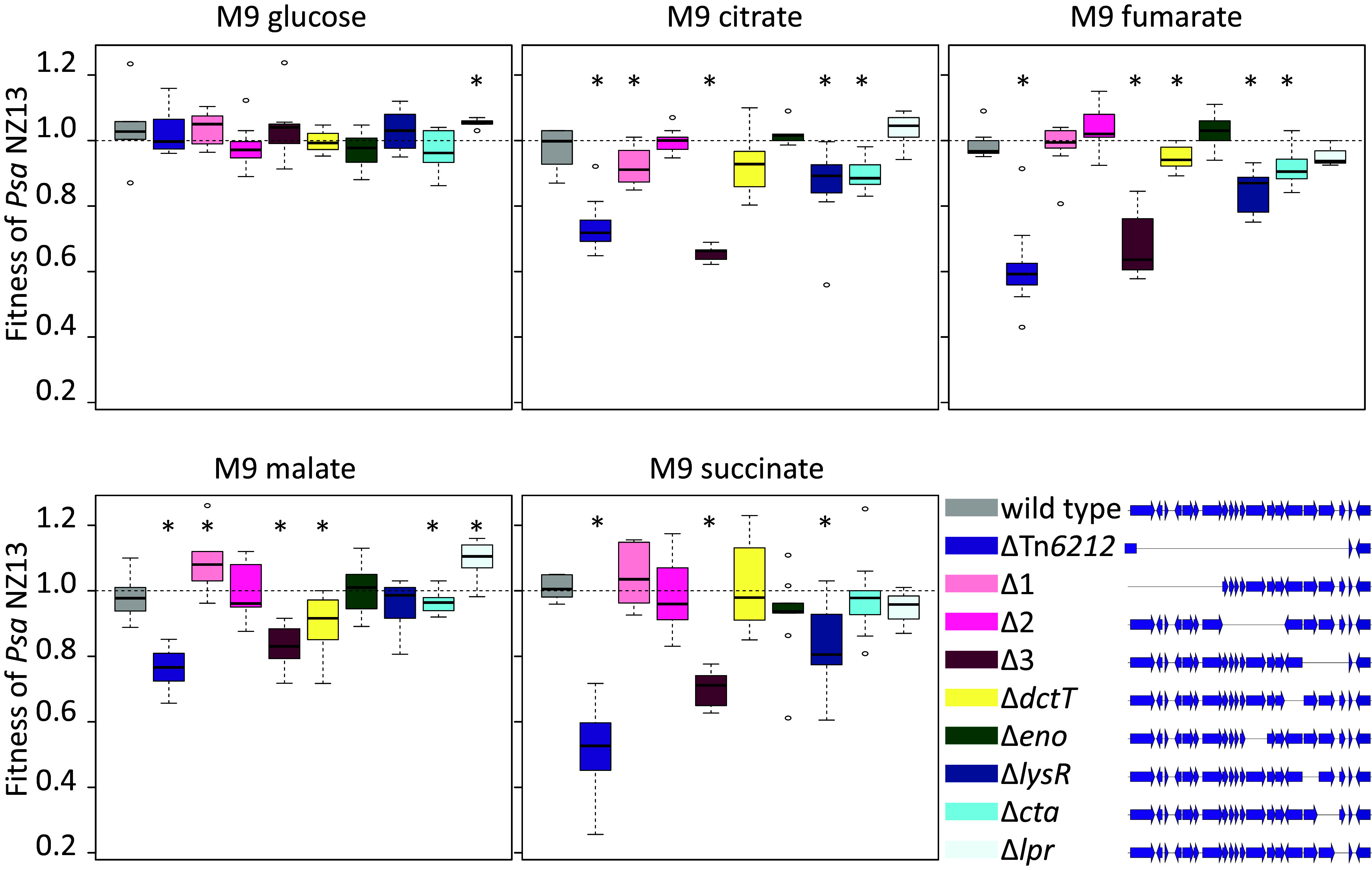
Competition assays on different carbon sources. Box plots showing the fitness of Tn*6212* mutants relative to *Psa* NZ13 *Psa* NZ::tn7-*lacZ*. Competition assays (1:1) were performed in M9 supplemented with different carbon sources; only day 4 is shown. Data for days 2, 3, and 4 are shown in *SI Appendix*, Fig. S8. Values smaller than 1 indicate a lower relative fitness of competitor. The experiment was performed with three replicates and repeated three times. From left to right: *Psa* NZ13 wild type, *Psa* NZ13ΔTn*6212*, *Psa* NZ13 Tn*6212*Δ1, *Psa* NZ13 Tn*6212*Δ2, *Psa* NZ13 Tn*6212*Δ3, *Psa* NZ13Δ*dctT*, *Psa* NZ13Δ*eno*, *Psa* NZ13Δ*lysR*, *Psa* NZ13Δ*cta*, and *Psa* NZ13*Δlpr*. * indicates that the difference in fitness is statistically significant (one-sided one-sample *t* test *P* < 0.05).

Although there was no evidence of DctT secretion, and no in planta phenotype was detected, the presence of genes whose products are associated with energy production and sugar utilization (enolase *eno*, inorganic pyrophosphatase, a catabolism associated protein *cta*, and *dctT*) nevertheless suggested that Tn*6212* is associated with bacterial growth and metabolism. To test this hypothesis, competitive fitness assays between *Psa* NZ13 and *Psa* NZ13ΔTn*6212* were performed in M9 minimal media supplemented with glucose or TCA cycle intermediates (citrate, succinate, malate or fumarate) as sole carbon sources. *Psa* NZ13ΔTn*6212* showed a significant reduction in fitness compared to the wild type in M9 containing TCA cycle intermediates, but not in glucose (Fig. 3 and *SI Appendix*, Fig. S8). In order to identify the genes responsible, three nonoverlapping deletion mutants were generated within different regions of Tn*6212* (Fig. 3): *Psa* NZ13 Tn*6212*Δ1 (~6-kb deletion including 8 genes), *Psa* NZ13 Tn*6212*Δ2 (~6-kb deletion including 7 genes), *Psa* NZ13 Tn*6212*Δ3 (~4-kb deletion including 3 genes and *xerC*), and *Psa* NZ13 Tn6*212*Δ*dctT* (*SI Appendix*, Table S5). *Psa* NZ13 Tn*6212*Δ3 displayed a growth deficit comparable to *Psa* NZ13ΔTn*6212* in all TCA intermediates, prompting further construction of single gene mutants in this region (*Psa* NZ13Δ*lysR*, Δ*cta*, Δ*lpr*). *Psa* NZ13Δ*lysR* and *Psa* NZ13Δ*cta* exhibited reduced growth on all TCA intermediates, although this reduction was not comparable to the growth deficit exhibited by the deletion of Tn*6212* (Fig. 3 and *SI Appendix*, Fig. S8). Curiously, *Psa* NZ13Δ*lpr* exhibited enhanced growth in M9 supplemented with citrate and malate. It thus seems *lysR* and *cta* interact synergistically with effects that are distinct from those caused by Δ*lpr*.

### Tn*6212* Has Global Effects on *Psa* NZ13 Gene Regulation.

After observing the contribution of Tn*6212* to *Psa* NZ13 fitness on TCA cycle intermediates, we asked whether Tn*6212* has an impact on the regulation of PsICE activity, and whether Tn*6212* instigates broader changes consistent with manipulation of host cell metabolism. The latter seemed conceivable given the presence of the versatile and promiscuous LysR regulator (38). RNA-seq was performed on *Psa* NZ13 and a set of nested deletion mutants (*Psa* NZ13ΔTn*6212*, *Psa* NZ13 Tn*6212*Δ3, and *Psa* NZ13Δ*lysR*), plus *Psa* NZ13Δ*dctT*. All strains were grown in M9 with glucose, citrate and succinate as sole carbon sources, and cells were harvested for RNA extractions once cultures reached late exponential growth phase. Transcriptional responses of each mutant were compared to wild type *Psa* NZ13 grown in the same media and genes and transcriptional responses were annotated with KEGG (39).

**Fig. 4.**
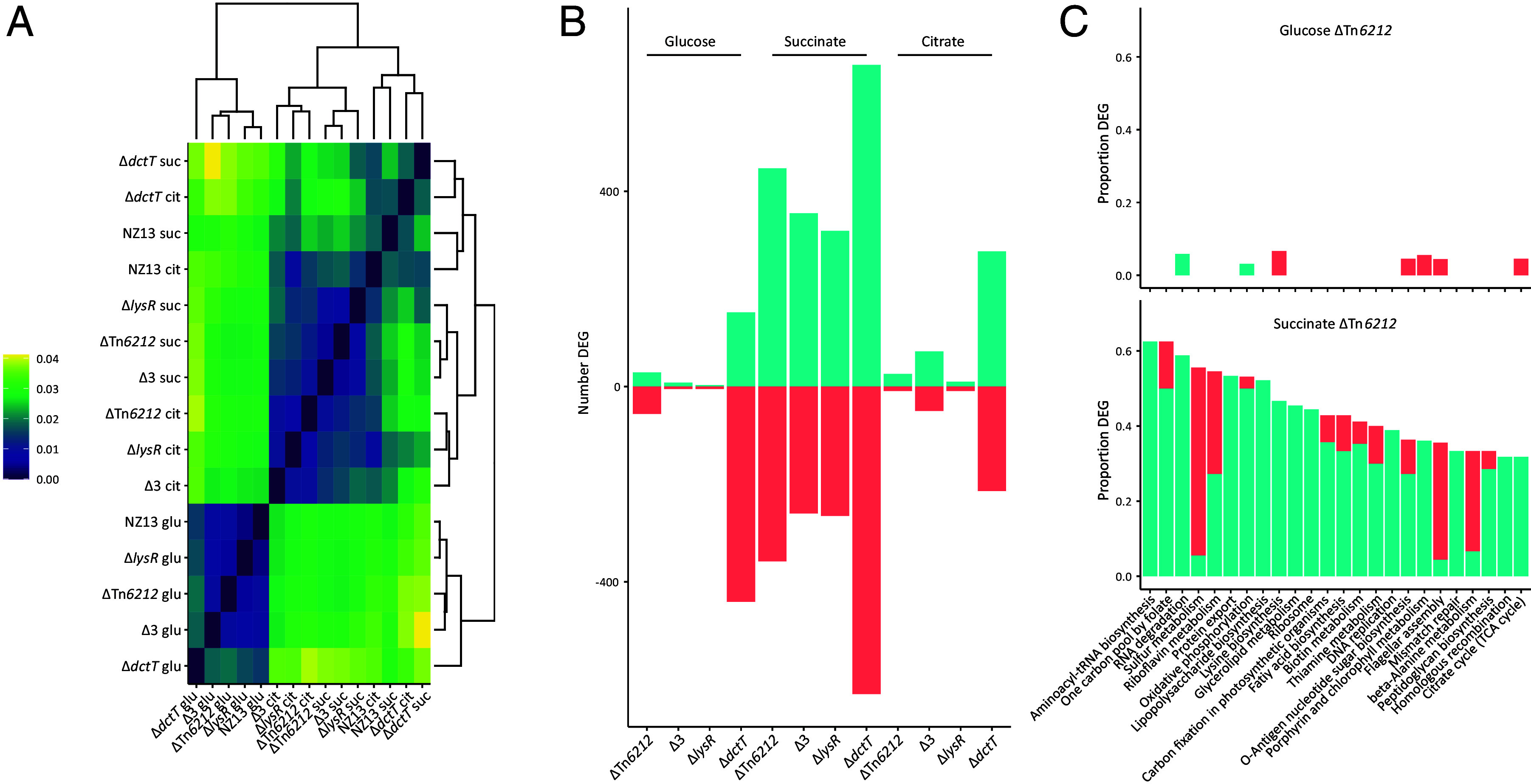
Tn*6212* alters bacterial host transcriptional responses. (*A*) For each RNA seq dataset the normalized mean coverage of every open reading frame encoded in the chromosome was calculated using Deseq2 (40). The Euclidean distance between the datasets was also calculated with each dataset being represented as a vector of normalized mean expression values. A distance of 0.04 means that expression differs on average by 4% per gene. The datasets cluster by carbon source used for growth except for NZ13 wild type and NZ13Δ*dctT* grown in succinate and citrate, which cluster by genotype rather than carbon source. (*B*) Number of differentially expressed genes in each of the genotypes grown in different carbon sources. Negative values indicate the number of genes that are significantly (*P* ≤ 0.05) underexpressed, and positive values the number of genes that are significantly overexpressed compared to the NZ13 wild type grown in the same carbon source. (*C*) KEGG functional categories that are significantly over- (turquoise) or under-expressed (red) in NZ13ΔTn*6212* compared to the wild type when grown in glucose and succinate. All functional categories containing at least 10 genes and where at least 30% of those genes are significantly differentially expressed are shown. We also show level 2 categories from the following top-level KEGG hierarchies: Environmental Information Processing, Cellular Processes, Genetic Information Processing, and Metabolism. For example, over 60% of all genes in the aminoacyl-tRNA biosynthesis pathway are overexpressed in NZ13ΔTn*6212* when grown on succinate compared to the NZ13 wild type. In contrast, there is not a single gene in the same pathway that is differentially expressed when grown in glucose.

A plausible null expectation is that strains cluster based on carbon source. The Euclidean distance plot displaying the normalized mean expression of the wild type and mutants grown in M9 supplemented with glucose, citrate, or succinate shows that this is only the case when strains are grown in glucose (Fig. 4*A*). *Psa* NZ13ΔTn*6212*, Tn*6212*Δ3, and Tn*6212*Δ*lysR* mutants share more similar expression profiles with each other when grown on citrate and succinate than with the wild type strain (Fig. 4*A*). This indicates the absence of Tn*6212*; *lysR*, *cta* and *lpr*; and *lysR* alone results in significant differences in expression compared to the wild type during growth on citrate and succinate. There is greater similarity between Tn6212Δ*dctT* and wild type expression on TCA intermediates than between Tn6212Δ*dctT* and other Tn*6212* mutants. This is at odds with the fact that the Δ*dctT* mutation is nested within Tn*6212* and thus expected to have just a subset of the effects wrought by the entire element. Its clustering with the wild type, *Psa* NZ13, suggests that the transcriptional effects of *dctT* are distinct from those caused by the totality of genes on Tn*6212*.

The number of differentially expressed genes (*P* < 0.05) and the relationship with genetic background and carbon source shows that the number of differentially expressed genes is greatest for strains grown on succinate (Fig. 4*B*). The magnitude of effects extends well beyond both Tn*6212* and the ICE, indicating that Tn*6212* manipulates host cell metabolism. Leaving aside *dctT*, whose deletion had no effects on fitness at later time points, the majority of regulatory effects can be directly attributed to *lysR*: in succinate, of the 792 genes whose expression is significantly altered on deletion of Tn*6212*, 411 are also affected in *Psa*NZ13Δ*lysR* (r = 0.978, *P* < 0.001) (*SI Appendix*, Fig. S9). For each carbon source, the number of differentially expressed genes is highest in *Psa* NZ13Δ*dctT*. This indicates that DctT functions, either directly or indirectly, as a repressor of genes on Tn*6212*. Given that DctT is a predicted transporter for dicarboxylic acids, it seems likely that the transporter is a conduit of information concerning the nature of the external environment that allows Tn*6212* to coordinate ensuing effects on host cell gene expression.

Making sense of the myriad transcriptional changes poses a major challenge. Of particular interest are those genes causally responsible for the observed changes. Fig. 4*C* shows the proportion of genes differentially expressed by Tn*6212* when grown on succinate and connection to various cellular functions as defined by the KEGG database resource (39). Data are ranked by the proportion of genes with significantly altered patterns of expression: the entire dataset with graphical mapping to KEGG pathways can be viewed at https://micropop.evolbio.mpg.de/data/2020_ICE/ with graphical mapping to KEGG pathways at https://micropop.evolbio.mpg.de/data/2020_ICE/kegg/. The number of genes whose expression is differentially affected by Tn*6212* when grown on glucose, for the same set of KEGG pathways, is shown for comparison. When grown on succinate, Tn*6212* significantly, and with primarily positive effects, affects the expression of genes in multiple KEGG pathways involved in translation (tRNA biosynthesis, one carbon pool by folate, protein export, and ribosome), posttranscriptional control (RNA degradation), energy metabolism (oxidative phosphorylation and sulfur metabolism), carbohydrate metabolism (amino sugar and nucleotide sugar biosynthesis and TCA cycle), metabolism of cofactors and vitamins (thiamine, riboflavin, and biotin metabolism), and DNA metabolism (DNA replication, mismatch repair, and homologous recombination).

Repressive effects are few, with notable exceptions being in sulfur metabolism, flagella assembly (and chemotaxis), and beta-alanine metabolism. Closer inspection of the affected genes shows that for sulfur metabolism, the activity of genes involved in production of sulfate or sulfite is decreased, whereas genes contributing to the synthesis of homocysteine and thus methionine show enhanced expression. For beta-alanine metabolism, genes responsible for conversion of beta-alanine to 3-oxopropanoate and then acetyl-CoA are repressed, but expression of pantoate-beta-alanine ligase, which converts beta-alanine to D-4-phosphopantothenate (and thus pantothenate), is significantly increased. The major repressive effect is on flagella assembly and chemotaxis where numerous genes are significantly repressed, although the magnitude of change is low (~1.5-fold decrease).

### Motility of Tn*6212* Mutants When Grown on TCA Cycle Intermediates.

We attempted to connect observed alterations in gene regulation to phenotypic changes by focusing on genes contributing to motility and chemotaxis. Measurement of cell swimming speed, directionality, and cell density in Adler chambers showed no significant differences between the mutant and wild type genotypes. Having failed to detect differences in cell-level behavior, we then examined the rate of radial expansion of wild type and ΔTn*6212* genotypes stab-inoculated with 10^5^ cells into semisolid M9 agar containing either succinate (at pH 7.0 and pH 6.0), glucose (pH 7.0) or casamino acids (CAA) (pH 7.0) as growth substrates. No significant difference in the rate of radial expansion was detected on M9 glucose (*P* = 0.97) or CAA (*P* = 0.25), but on M9 succinate, carriage of Tn*6212* significantly increased the rate of radial expansion, with the highest rate evident at pH 6.0 (*P* = 0.009 and *P* < 0.001, at pH 6.0 and pH 7.0, respectively). We observed substantial variability among all genotypes in growth initiation time using an initial density of 10^5^ cells on glucose. Whereas growth on succinate or CAA was readily detectable by 1 d for all replicates starting at 10^5^ cells, the initiation of growth in glucose was highly variable, requiring at least 4 d, and in one replicate, no growth was visible even at the final time point of 8 d. Suspecting density-dependent behavior, an additional treatment was included in which 10^6^ cells were used to establish the centrally located population. This increased inoculum density largely eliminated variation in growth initiation and showed that the presence of the Tn*6212* decreased the rate of radial expansion on glucose (*SI Appendix*, Fig. S10).

## Discussion

ICEs associated with the *P. syringae* species complex were first identified in 2000 as “pathogenicity islands” whose spontaneous excision caused switches in virulence phenotypes of *P. syringae* pv. *phaseolicola* (21). Since 2000, awareness of ICEs as vehicles that move ecologically significant genes has rapidly grown, with particular evidence of diversity and horizontal transfer coming from genomic analysis of *Psa* strains associated with the global kiwifruit canker disease pandemic (15, 19), and dramatic evidence of their impact arising from study of copper resistant strains in New Zealand (16). Here, our bioinformatic analyses show that the *P. syringae* complex harbors numerous and diverse ICEs of the same family (PsICEs) distributed across a range of PGs. Although core genes show overall synteny, genes of potential ecological relevance are found in defined CR.

Comparative analyses clearly show that ICEs are facilitators of horizontal transfer, mediating movement of diverse sets of genes among a wide range of bacterial hosts and over significant spatial scales. Near-identical PsICEs were found in hosts sampled decades and thousands of kilometers apart, and near-identical host strains carry PsICEs with backbone genes that share less than 95% average nucleotide identity, a threshold commonly used to define distinct bacterial species (41). While PsICEs are defined by a conserved set of core genes, they carry variable accessory genes likely to confer ecologically significant functions to hosts. In some instances, these accessory genes show matches to known genes, but many are either function unknown or have no homologue in databases. The relatedness of PsICEs to those from *P. aeruginosa* raises the possibility that ICEs might move genes between plant and opportunistic human pathogens, although minimally overlapping ecological niches likely limit ICE movement across larger phylogenetic distances.

Many PsICEs carry accessory genes that confer a selective advantage to agricultural plant pathogens, like antimicrobial resistance and virulence-associated genes. The identification of cargo genes relevant to the bacterial host niche is not unusual; STX-R391 ICEs of clinical origin typically carry antibiotic resistance genes (42), while STX‐R391 ICEs from free-living marine bacteria *Alteromonas* mainly encode for metal resistance and restriction modification systems (43). We have previously shown that copper resistance genes on a PsICE confer a growth advantage to *Psa* on leaf surfaces sprayed with copper (16). The widespread application of bactericides, insecticides, and herbicides is likely to select for the maintenance of copper and arsenic resistance genes after ICE acquisition. Type 3 secreted effectors (T3SE), known to play an important role in disrupting plant host recognition and immune responses, are also present on ICEs. The very first ICE characterized in the *P. syringae* species complex (ICEPph1302A, previously named PPHGI-1) carries *hopAR1*, which is also present on ICEPs304-1 and ICEPfm207. ICEPs248-8 carries *avrRpm1*, ICEPsaMAFF212036 harbors *hopAU1*, and ICEPatATCC11528-att2 carries three effector genes: *hopF2*, *hopO1-1,* and *hopT1-1*. Notably, all of these effectors are known to elicit effector-triggered immune responses. The transfer of a recognized T3SE onto an ICE may allow the pathogen to evade plant host recognition by silencing or maintaining the virulence gene at low frequencies in the population (44). In our analysis, the most common cargo carried by PsICEs is not associated with agricultural sprays or plant host immunity but is rather a mobile genetic element associated with the transport, regulation, and metabolism of preferred carbon sources including succinate and other TCA cycle intermediates (Tn*6212*).

The widespread occurrence and high sequence identity of Tn*6212* from diverse ICEs indicates that it recently invaded ICEs circulating among the species complex and likely confers a selective advantage to *P. syringae*. We show here that Tn*6212* enhances bacterial fitness on TCA cycle intermediates, which are abundant in plant tissues and up-regulated during pathogen invasion (45). The Tn*6212* dicarboxylic acid transporter DctT and transcriptional regulator LysR may sense shifts in carbon source availability and initiate a signaling cascade altering the expression of chromosomal genes. Accordingly, the recent global dissemination of Tn*6212* is likely connected to capacity of the element to manipulate bacterial host cell metabolism and rapidly shift resources toward growth. The widespread impact on chromosomal gene expression is a challenge to interpret; however, examination of patterns of altered expression, functional categories, and connections to genes carried on Tn*6212* suggest that the mechanism behind altered fitness caused by Tn*6212* may reside in RNA degradation.

**Fig. 5.**
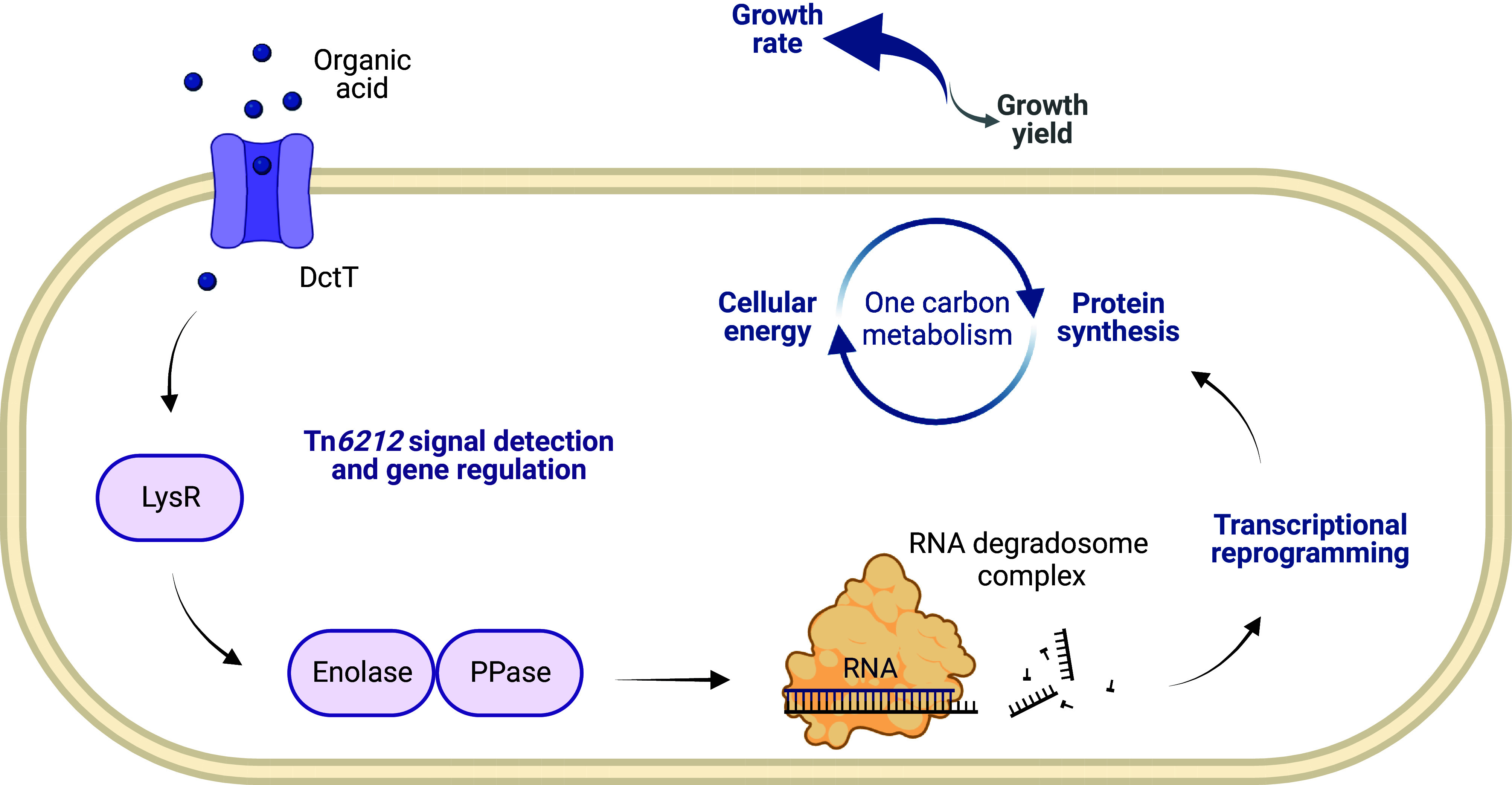
Model of Tn*6212*-ICE-bacterium–plant interactions. The Tn*6212* C_4_-transporter DctT and transcriptional regulator LysR are predicted to sense shifts in carbon source availability and initiate a signaling cascade altering the expression of chromosomal and Tn*6212*-encoded genes. The expression of genes involved in the RNA degradosome complex, such as the genes encoding for enolase, inorganic pyrophosphatase, RNAse E, and two DEAD/DEAH box helicases, is induced. Subsequent to posttranscriptional control activity of the RNA degradosome, the expression of genes involved in TCA cycle and gluconeogenesis is stimulated. This transcriptional reprogramming rapidly redistributes incoming carbon to maximize ATP, promoting growth.

Tn*6212* encodes the glycolytic enzyme enolase, a major component of the RNA degradosome, connecting the physiological status of the cell to RNA degradation (46), and enolase is significantly overexpressed in the *Psa* NZ13 Tn*6212*Δ3 mutant [and with elevated expression in Δ*lysR* (*P* = 0.90)]. Tn*6212* also encodes an inorganic pyrophosphatase that contributes to RNA degradosome function with the possibility of additional contributions from uracil-DNA glycosylase. Beyond genes encoded by Tn*6212*, all major additional components of the RNA degradosome, including RNAse E and two different DEAD/DEAH box helicases are significantly overexpressed in *Psa* NZ13 growing on succinate, compared with ΔTn*6212*. Further reason to suggest a role of the RNA degradosome stems from its role in posttranscriptional regulation. Growth on succinate triggers the post-transcriptional repression of mRNAs involved in the use of alternate carbon sources (47, 48). Tn*6212* may strengthen carbon catabolite repression in some manner, perhaps hastening target mRNA degradation while up-regulating genes involved in organic acid acquisition and utilization, rapidly redistributing incoming carbon to maximize ATP, promoting growth (Fig. 5).

Our results suggest Tn*6212* enhances utilization of preferred carbon sources present in the plant host and up-regulated during pathogen invasion (45). We did not, however, identify a strong phenotype for Tn*6212* in our pathogenicity tests on kiwifruit plants. This may be due to a number of factors. First, Tn*6212* does not confer a completely new phenotype to the pathogen, but may instead accelerate bacterial detection and response to the presence of preferred carbon sources. Plant-based assays are not suitable to detect small but significant effects on pathogen fitness, as fitness is a complex phenotype in which multiple traits may contribute. The timing and duration of the phenotype may not be observable under the conditions we assayed, particularly if Tn*6212* is involved in other stages of plant colonization, such as epiphytic growth, even in non-host plants. The rapid detection and response to fluctuating levels of preferred carbon sources could also enhance the fitness or persistence of *P. syringae* in non-plant-associated environments like rivers, snowpack, and even clouds (8, 9).

The rapid redirection of resources to maximize growth may enhance the fitness of bacterial strains carrying ICEs with Tn*6212*. It is possible this growth advantage also underlies ICE-mediated dissemination of Tn*6212*. In a mixed population of bacteria that are either ICE-less or carrying ICEs without Tn*6212*, the subpopulation of cells carrying ICE with Tn*6212* may be overrepresented once the population reaches the stationary phase, when ICE transfer is more likely to occur. Enhanced ICE dissemination— and for that matter, enhanced virulence—may be a by-product of the effect of Tn*6212* on bacterial growth in plant tissues. ICEs may therefore contribute to bacterial adaptation by adjusting bacterial transcriptional responses to preferred carbon sources.

## Materials and Methods

### Identification and Assembly of ICEs.

A broad family of *P. syringae* ICEs (PsICEs) was identified using BLASTn searches of a collection of sequenced *Psa* genomes, combined with genomes deposited in the NCBI Genbank and WGS databases (updated to July 2021 and November 2017, respectively) (20). When matches were identified in draft assemblies, contigs were downloaded and used to join contigs that overlapped by at least 6 bp. PsICEs sequences are available at https://github.com/EC-Rufina/PsICEs. Ten additional PsICEs were identified but discarded from the analysis because the elements did not encode a conjugative system. To delineate the chromosomal integration sites, flanking sequences were inspected for direct repeats sites which form when ICEs integrate in their *att* site. The broader family of ICEs was defined with tBLASTn (20) searches in the NCBI GenBank WGS database (updated to April 2017). The DEAD-box helicase from ICEPsaNZ13 (IYO_024645) was used as the query, retaining hits with minimum 81% amino acid identity, excluding all hits in *P. syringae*.

### PsICE Structure and Classification.

REALPHY (49) was used to examine and classify PsICE diversity, identifying nonredundant PsICEs. REALPHY produced a final 1,975 bp alignment, which was in turn used to cluster similar PsICEs by building a Neighbor Joining (NJ) tree with 100 bootstrap replicates (50). The NJ tree was rooted at midpoint using the midpoint() function from the phangorn package in R (51). The NJ tree was used as a guide for the selection of a reduced set of representative, nonredundant PsICEs. Nonredundant ICEs may vary in multiple ways: 1) they share identical backbone genes but differ according to their complement of accessory genes, 2) they share the same accessory genes but the backbone genes have average pairwise nucleotide identity values lower than 95%, or both (3). For example, ICEPsaI12.29 was selected as nonredundant because even though it falls in the same clade of ICEPsaI10, ICEPsaI12.29 does not carry Tn*6212*. The resulting set of 53 nonredundant PsICEs was used for subsequent analyses. The identification of conserved backbone genes in PsICEs was performed using the pangenome identification tool ROARY (26). Backbone genes are here defined as genes present in at least 93% of all nonredundant PsICEs. MAFFT alignments using automatic alignment parameters (52) were used to examine structural conservation of the backbone genes and identify sites of accessory gene integration. Alignments were then separately generated for 58 backbone genes using MAFFT with automatic parameters (52). Phylogenetic incongruence between individual backbone genes was evaluated using the ILD test (27) using PAUP* (53). The extent of inter-PsICE recombination was evaluated with Neighbor-Net (29) using SplitsTree (54). ClonalFrameML was also used on the concatenated alignment of the backbone genes (28). Alfy 9 (31) was used to assess the inter-ICE recombination.

### Bacterial Host Phylogeny.

A PhyML tree (55), using default parameters and 100 bootstrap replicates, was built using the concatenation of the alignments of the housekeeping genes *gapA*, *gltA*, *gyrB,* and *rpoD* of each *P. syringae* genome, *P. fluorescens* SBW25 (AM181176.4) was used as outgroup.

### Deletion Mutant Generation and Plant and Bacterial Growth Conditions.

*Pseudomonas* strains were routinely grown in KB at 28 °C, *Escherichia coli* in LB at 37 °C, and *Agrobacterium tumefaciens* in LB at 28 °C. *N. benthamiana* and *A. thaliana* Col0 assays were performed as previously described (56). Deletion mutants in genes and regions of ICEPsaNZ13 (*SI Appendix*, Table S5) were constructed by marker exchange mutagenesis as described in ref. 37 with plasmids and primers listed in *SI Appendix*, Tables S6 and S7. A *lacZ* reporter gene (*SI Appendix*, Table S6) was introduced into *Psa* NZ13 for competition experiments via triparental mating as in ref. 16.

### Bacterial Protein Secretion and Host Recognition Assays.

Two plasmids (pMT-1 and pMT-2) were constructed by GenScript® using vector pUCP22. Each plasmid was introduced into both *Psa* NZ13 and *Psa* NZ13Δ*hrcC* (37) via triparental mating. Binary vectors carrying the DctT:AvrRpt2 constructs were created to confirm truncated or full-length DctT did not interfere with AvrRpt2-mediated recognition of secreted proteins. The *dctT:avrRpt2* was fused to a C-terminal epitope tag (3xFlag) and introduced into pICH86988 using Golden Gate cloning (57). The plasmids were electroporated into *A. tumefaciens* AGL1 as described in ref. 56.

HR assays were carried out in *A. thaliana* Col-0 with strains diluted in 10 mM MgCl_2_ to a final OD_600_ of 0.2 as described in ref. 56. The experiment was repeated twice. Ion leakage experiments were carried out as in ref. 56. *Agrobacterium* infiltration was used to transiently express DctT:AvrRpt2 constructs in *N. benthamiana*, along with *A. tumefaciens* strains carrying functional RIN4 and RPS2 expression constructs (AGLRIN4 and AGLRPS2, respectively) (58) as previously described (56).

### Competition and Growth Experiments.

Competition experiments between wild type *Psa* NZ13, *Psa* NZ13::*lacZ,* and ICE mutant genotypes were performed in vitro using minimal M9 medium (59) supplemented with glucose (10 mM), fumarate (10 mM), citrate (10 mM), malate (20 mM), or succinate (10 mM) as sole carbon sources. 100 µL of washed cells at OD_600_ 0.2 were used to inoculate 10 mL of M9 media (final OD_600_ of 0.002). Cultures were shaken at 250 rpm at 28 °C, measuring bacterial density at 0, 2, 3, and 4 d by plating dilutions on KB amended with X-gal (60 µg mL^−1^) to distinguish between deletion mutants (white colonies) and ancestral *Psa* NZ13 marked with *lacZ* (blue colonies) (Dataset S1). The experiment was performed using three replicates and repeated three times. The fitness of each strain in the competition experiments is expressed as the Malthusian parameter (60).

### RNA Extraction, Sequencing, and Analysis.

Strains were streaked to single colonies on KB plates and incubated at 28 °C for 48 to 72 h. Single colonies were used to inoculate 5 mL of M9 medium supplemented with 20 mM glucose, citrate, or succinate. Liquid cultures were set up with 3 replicates per strain per carbon source and shaken at 230 rpm at 28 °C. Cultures were incubated for 26 h (M9+succinate), 28 h (M9+citrate) or 48 h (M9+glucose). Cultures were then diluted into fresh media using three dilutions per sample to ensure collection at mid-log phase: 1:10, 1:25, and 1:50 (M9+glucose and M9+succinate) or 1:10, 1:20, and 1:40 (M9+citrate). Cells were collected for RNA extraction at OD_600_ between 0.4 and 0.5. RNA was extracted using the RNeasy Mini Kit (Qiagen 74106) according to the manufacturer’s instructions. Samples were further treated with the Turbo DNA-free TM kit (ThermoFisher AM1907) according to the manufacturer’s instructions. 210 ng total RNA was used for rRNA depletion with the bacterial Ribo-Zero kit (Illumina 20037135), according to the manufacturer’s recommendations. After rRNA depletion, the remaining RNA was fragmented, and Illumina-compatible libraries were prepared using the NEBNext Ultra™ II Directional RNA Library Prep Kit for Illumina (New England Biolabs E7760S). Libraries were sequenced on the Illumina HiSeq3000 system. RNA sequencing data were analyzed as described in ref. 61. Genes with statistically significant differential expressions (*P* < 0.05 and *P* < 0.01) were annotated with KEGG pathways (39) and visualized using scripts stored at https://gitlab.gwdg.de/guilhem.doulcier/pseudomonas_rnaseq/. The results are available for browsing at https://micropop.evolbio.mpg.de/data/2020_ICE/ and https://micropop.evolbio.mpg.de/data/2020_ICE/kegg/).

### Phenotypic Characterization of Motility on TCA Cycle Intermediates.

Wild type and ΔTn*6212* mutant cells were grown in succinate and placed in Adler chambers with succinate or casamino acids (CAA) as attractants and a PBS buffer control. Time-resolved data were collected by imaging at regular intervals (either 5-min intervals over the course of 1 h or 500 ms over 15 s). The resulting images were subject to analyses that included measurement of cell swimming speed, directionality, and cell density at the moving swarm. The rate of radial expansion of both wild type and ΔTn*6212* cells was measured by stab-inoculating cells into semisolid M9 agar containing either succinate (at pH 7.0 and pH 6.0), glucose (pH 7.0), or CAA (pH 7.0) as growth substrates. Stab inoculation was performed using an initial cell density of either 10^5^ or 10^6^ cells, measuring radial expansion daily for 8 d using precision calipers. The experiment was performed twice with, on each occasion, five replicates per treatment.

## Supplementary Material

Appendix 01 (PDF)

Appendix 02 (PDF)

Dataset S01 (XLSX)

Dataset S02 (XLSX)

## Data Availability

Sequencing data have been deposited in NCBI. All other data are included in the manuscript and/or supporting information.
