# Supplementary material for: Rapid dissemination of host metabolism–manipulating genes via integrative and conjugative elements
Source: Proc Natl Acad Sci U S A. 2024 Mar 8;121(11):e2309263121. doi: 10.1073/pnas.2309263121 (PMC10945833; doi:10.1073/pnas.2309263121)
Supplement: Supplementary file 1 — Appendix 01 (PDF) [file pnas.2309263121.sapp1.pdf]

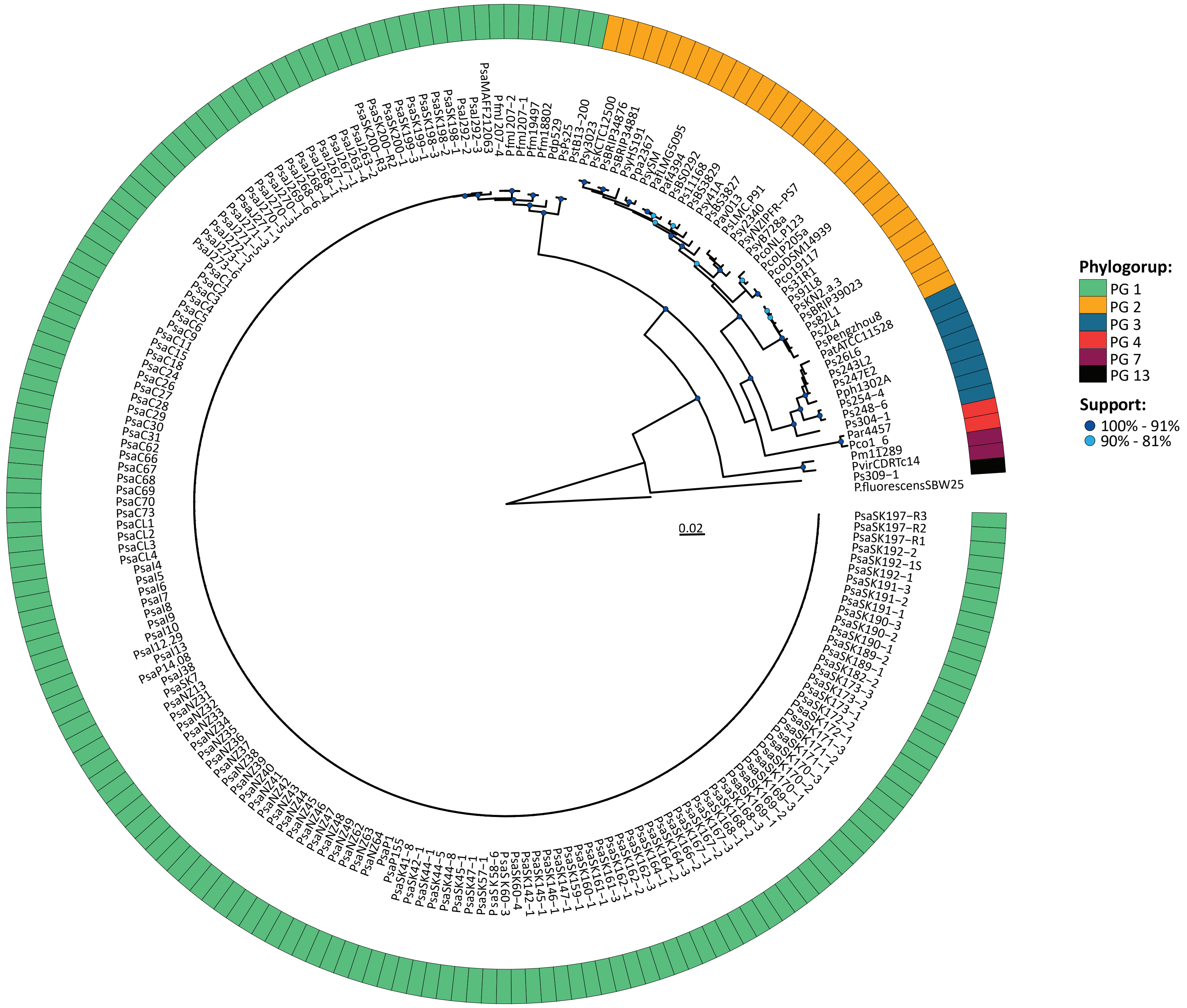


**Fig. S1. Phylogeny of the strains harbouring the PsICEs.** PhyML tree based on the concatenation of *gapA*, *gltA*, *gyrB* and *rpoD*. *P. fluorescens* SBW25 was used as outgroup. The ring displays the phylogroup of the *P. syringae* species complex strain. Support indicates bootstrap support values. Scale bar indicates substitution per site.

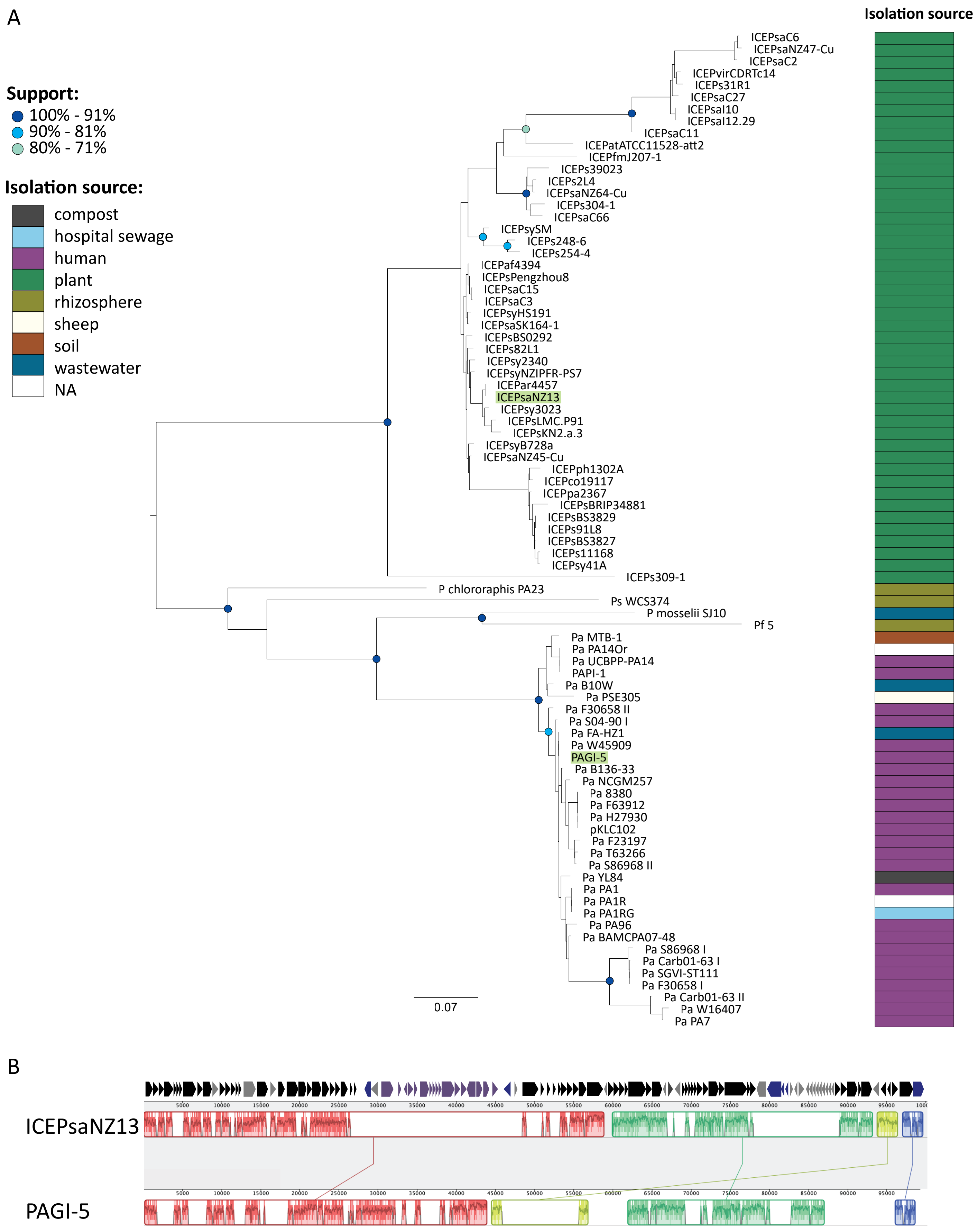


**Fig. S2. PsICEs are members of a broad family of ICEs circulating in the genus *Pseudomonas*.** A) *parA*, *topIII* and *xerC* were aligned with MAFFT and concatenated. After gaps removal, the maximum-likelihood tree was built with PhyML and the tree was rooted at midpoint. Support indicates bootstrap support values. ICEs in other species are identified according to accepted names (e.g. 'PAGI-5', 'pKLC102') or using the species/pathovar prefix and strain ID suffix (e.g. 'PaPA1'). Scale bar indicates substitution per site. B) A progressiveMauve alignment of entire ICEPsaNZ13 (representative of *P. syringae* ICEs) and entire PAGI-5 ICE (representative of the *P. aeruginosa* pKLC102/PAGI-2 family of ICEs). Colored regions in the progressiveMauve alignment indicate LCBs (locally colinear blocks) across the two ICEs. Colourless stretches indicate ICE unique regions. The genes in the map of ICEPsaNZ13 are colored as in Figure 2.

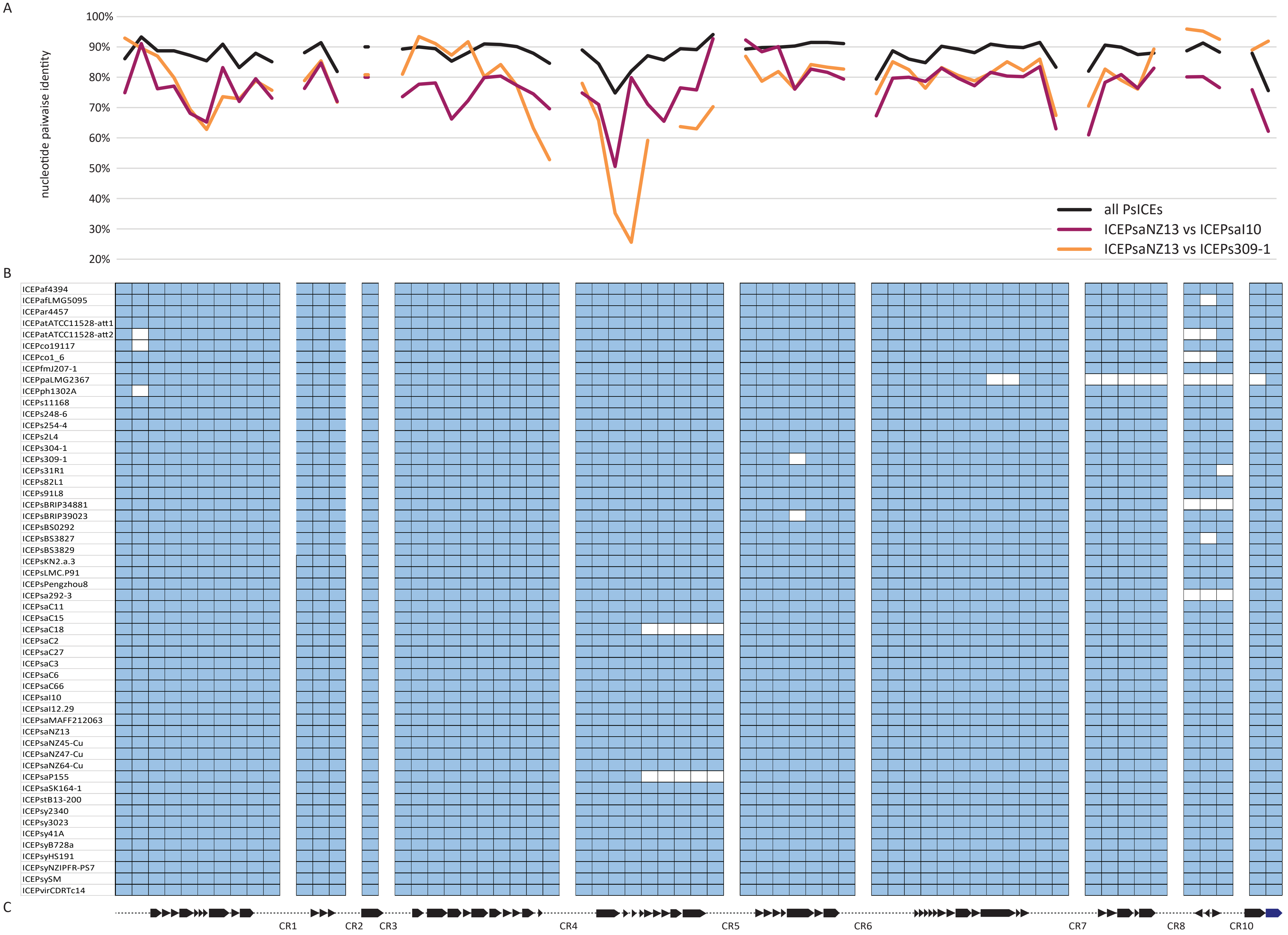


**Fig. S3. The backbone genes of the PsICEs.** A) Nucleotide pairwise identity of each backbone gene. B) Graphical representation of the backbone genes. C) Presence and absence of backbone genes in the non-redundant PsICE. In light blue gene presence, in white gene absence. CR stands for cargo region.

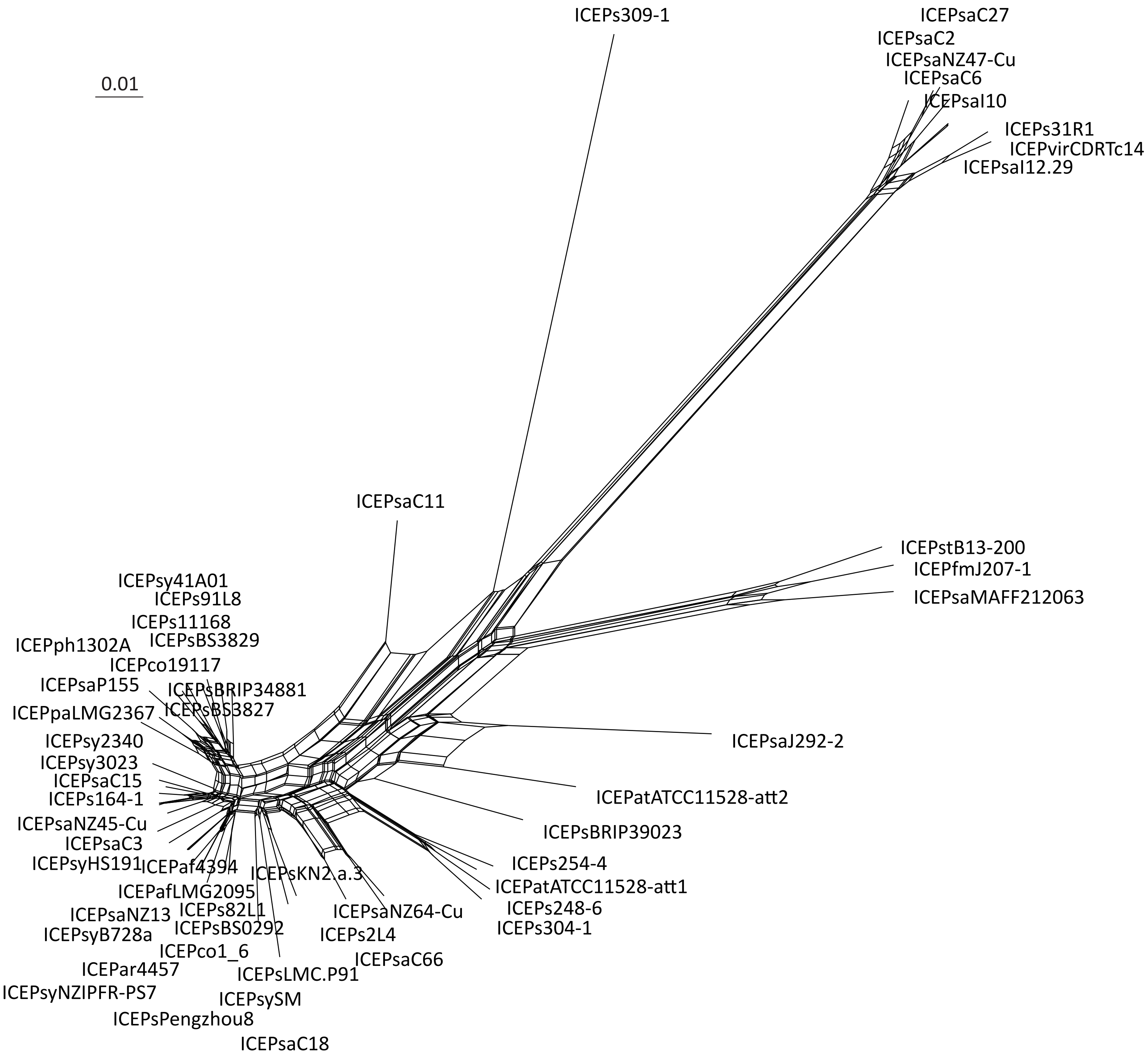


**Fig. S4. Neighbor-net network tree of backbone genes of PsICEs.** Neighbor-Net generated in Splitstree using a concatenated alignment of 58 backbone genes conserved in all 53 non-redundant ICEs. Scale bar indicates substitution per site.

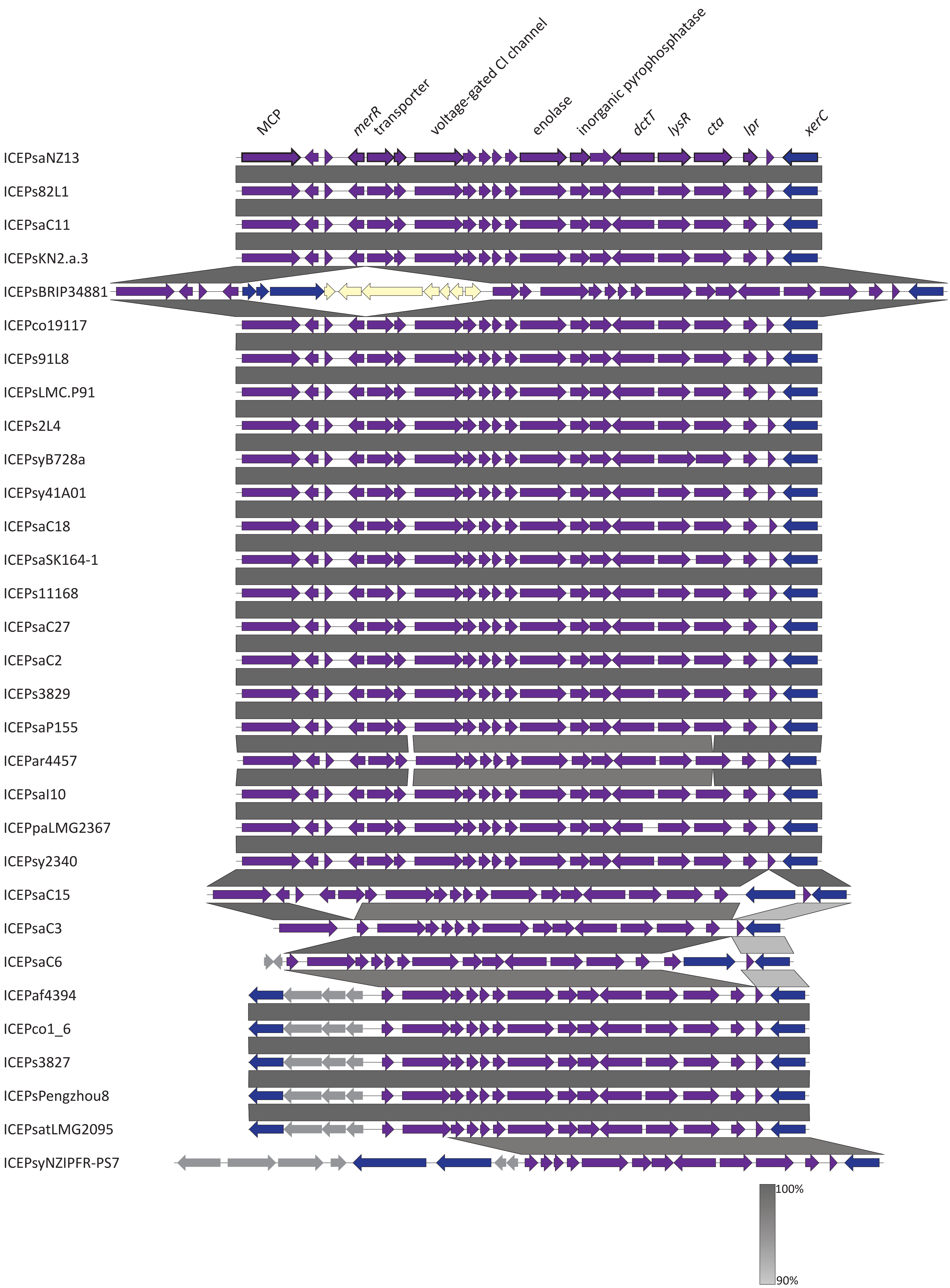


**Fig. S5. Conservation of Tn*6212*.** The percentage of sequence similarity is indicated by the intensity of the gray color. Vertical blocks between analyzed sequences indicate regions with at least 90% of similarity.

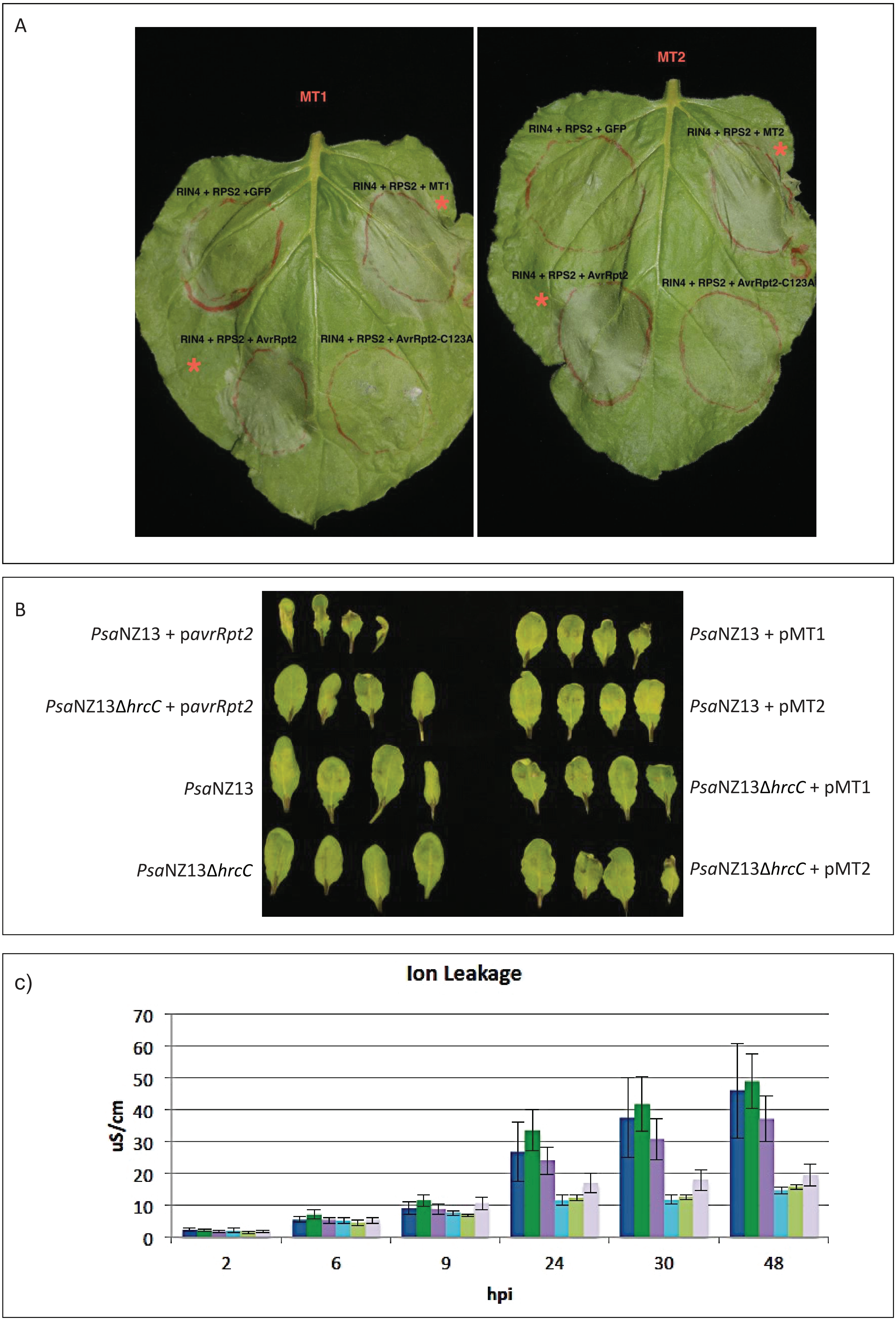


**Fig. S6. Tn*6212* DctT secretion assays.** A) Agroinfiltration in *Nicotiana bethamiana* leaves using the indicated expression constructs. Pictures were taken at 24 hpi, an asterisk indicates hypersensitive response (HR) observed. B) Secretion assay in *Arabidopsis thaliana*. Leaves of *A. thaliana* Col-0 were pressure infiltrated with the indicated strain and HR development recorded at 48 hpi. The experiment was repeated two times inoculating two leaves of three plants per strain used. C) Ion leakage in *A. thaliana*. Conductivity (µS/cm) of solution containing leaf discs inoculated with *Psa* N13 (blue bars), *Psa* NZ13 + pMT-1 (dark green bars), *Psa* NZ13 + pMT-2 (purple bars), *Psa* NZ13 Δ*hrcC* (azure bars), *Psa* NZ13 Δ*hrcC* + pMT-1 (light green bars) and *Psa* NZ13 Δ*hrcC* + pMT-2 (lilac bars). Data are means and standard deviation of four replicates.

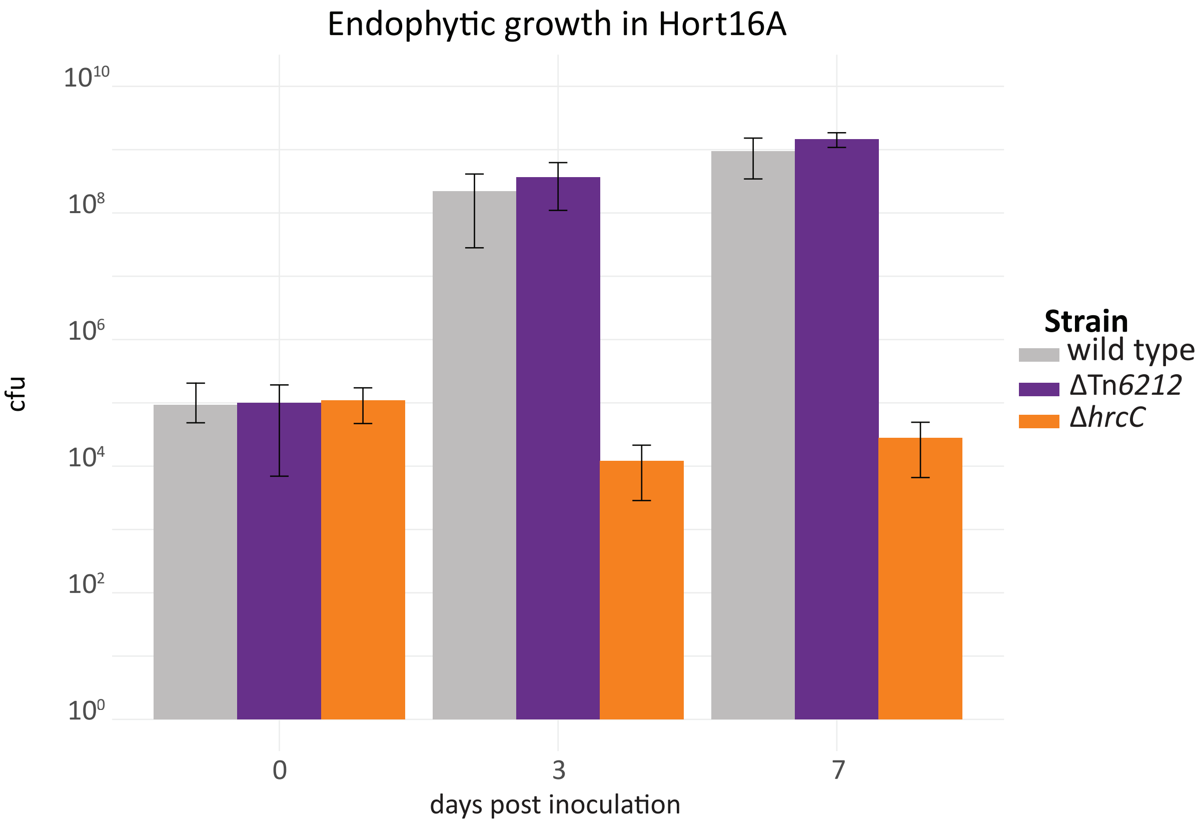


**Fig S7. Growth of *Psa* NZ13 and *Psa* NZ13 ΔTn*6212* on kiwifruit.** Growth of *Psa* NZ13 (grey bars), *Psa* NZ13 ΔTn*6212* (purple bars) and *Psa* NZ13 Δ*hrcC* (orange bars) was assessed endophytically on leaves of the kiwifruit cultivar Hort16A. Data are means and standard deviation of five replicates. Two tailed t-test revealed no statistical difference ($P>0.05$) between *Psa* NZ13 and the Tn*6212* mutants.

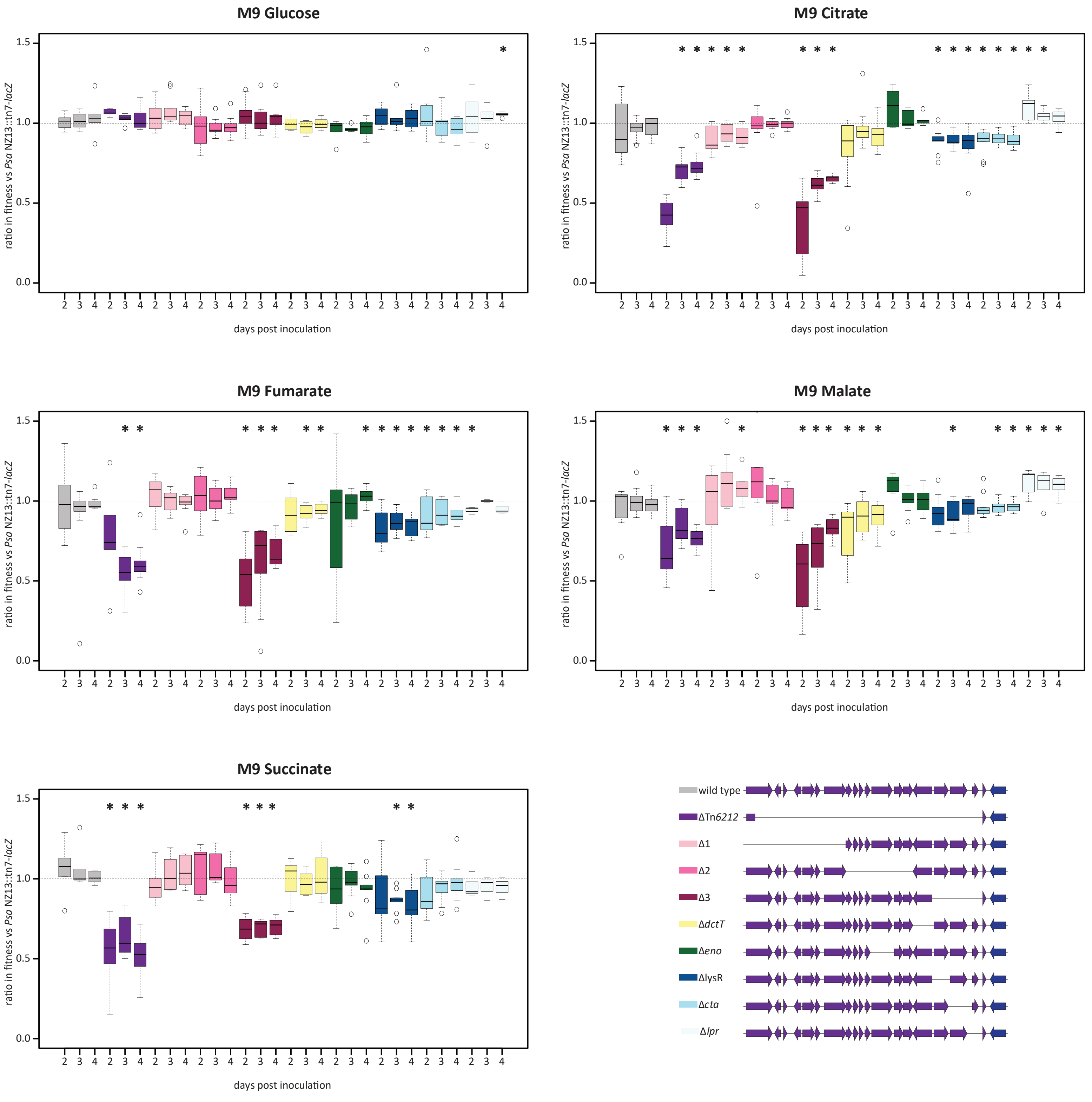


**Fig. S8. Tn*6212* mutant fitness on multiple carbon sources.** Box plots of the fitness of the *Psa* NZ13 and the mutants on Tn*6212* versus *Psa* NZ13::tn7-*lacZ*. Competition assays were established using equal starting densities (1:1) in M9 minimal medium supplemented with different carbon sources, measuring wildtype to mutant growth at 2, 3 and 4 days post inoculation. Values smaller than 1 indicate the competitor exhibits lower fitness relative to the wildtype strain. The experiment was performed with three replicates and repeated three times. From left to right, *Psa* NZ13 wild type, *Psa* NZ13ΔTn*6212*, *Psa* NZ13 Tn*6212*Δ1, *Psa* NZ13 Tn*6212*Δ2, *Psa* NZ13 Tn*6212*Δ3, *Psa* NZ13 Δ*dctT*, *Psa* NZ13 Δ*eno*, *Psa* NZ13 Δ*lysR*, *Psa* NZ13 Δ*cta*, *Psa* NZ13 Δ*lpr*. An asterisk indicates the fitness difference is statistically significant (one-sided one sample t-test $p < 0.05$).

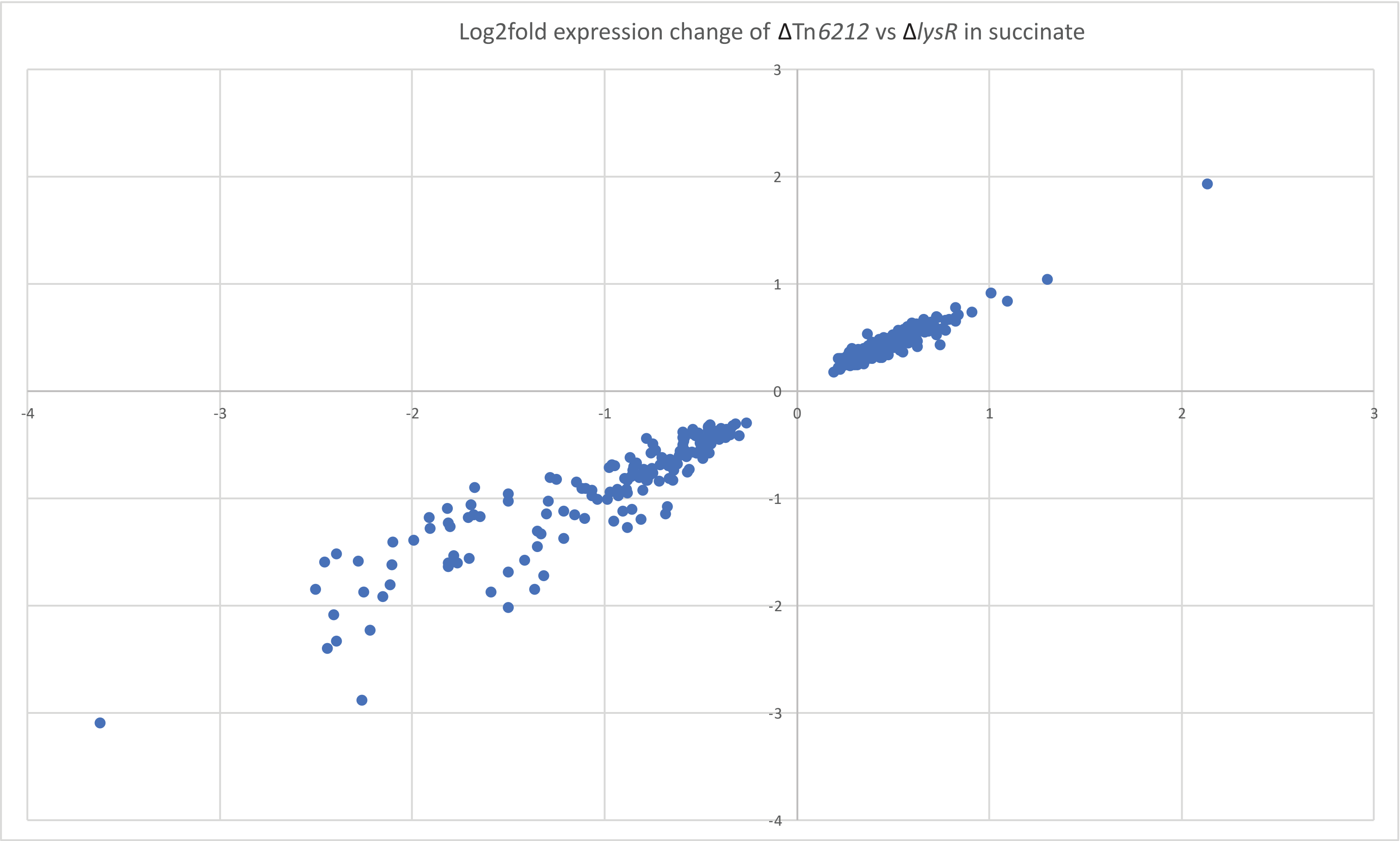


**Fig. S9. Correlation between genes exhibiting significant expression fold change in *Psa* NZ13ΔTn*6212* and *Psa* NZ13 Δ*lysR*.** Log2-fold expression change of genes exhibiting significant differences in both ΔTn*6212* (x-axis) and Δ*lysR* (y-axis) strains during growth on succinate.

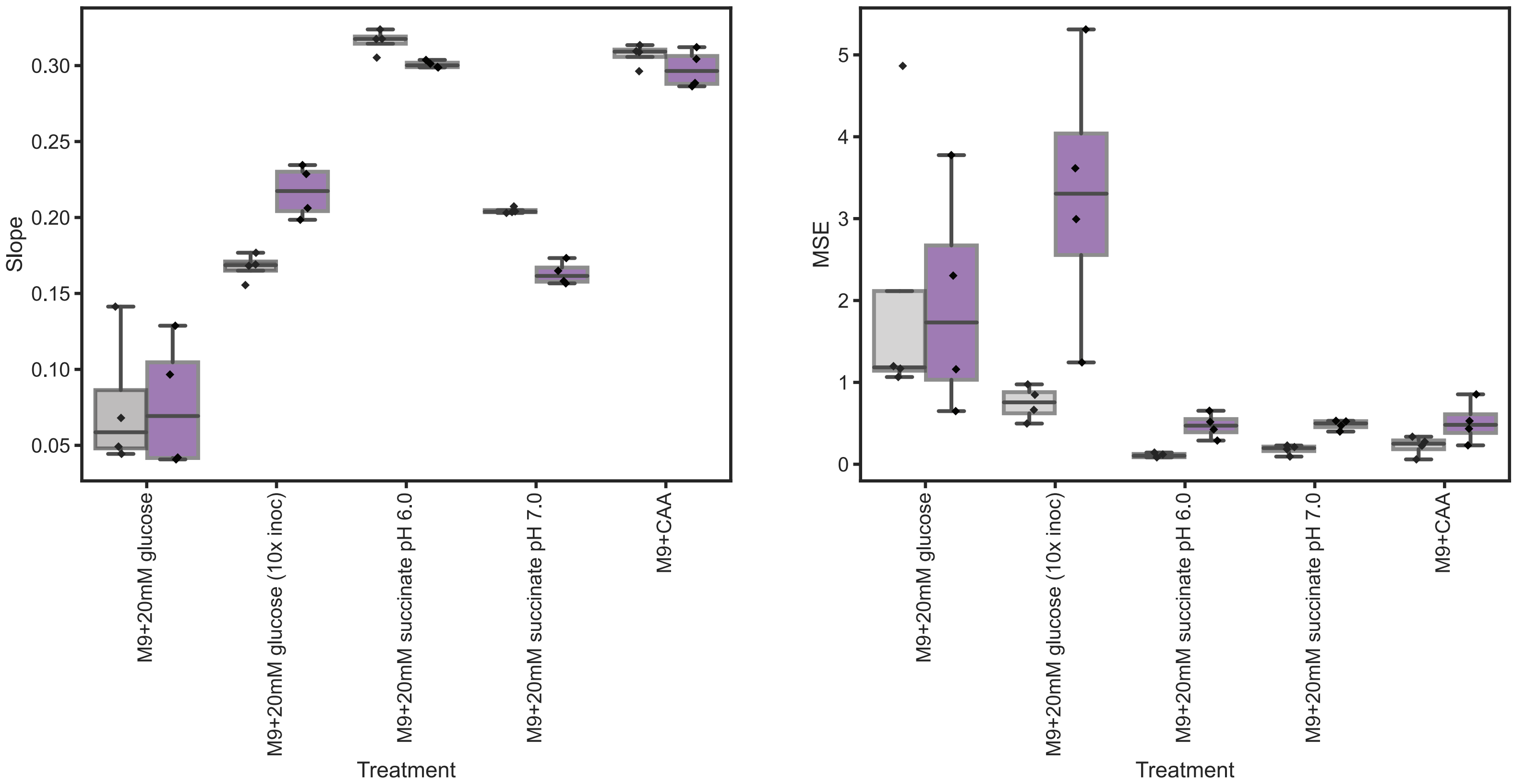


**Fig. S10. Colony expansion rate on multiple carbon sources.** The dynamics of colony growth was fitted with a line (Swarm = Slope * t + Intercept). Here, we show the statistics of Slope parameter (left panel), which is the expansion rate of colonies. The mean square error (MSE) is the average squared difference between the predicted fit and the actual data value, which is an estimator of the quality of fit. On panels, each colony is represented by a dot. They are grouped by the treatment (x-axis) and genotype (bar color: grey – Psa NZ13, purple – ΔTn6212). The boxplots display datasets based on the five-number summary: the minimum, the maximum (shown as whiskers), the sample median (central line), and the first and third quartiles. The parameter R^2 is the fraction of the variation of data explained by the linear model. R^2 > 0.8 for all replicates and genotypes indicates linear relationship of the colony expansion dynamic. Statistical significance was calculated with the t-test, the experiment was performed with 5 biological replicates.

Statistical analysis using Student's t-test tested whether the mean expansion rate statistically differs between genotypes for the same treatment, where H0: means of expansion rates of Psa NZ13 and ΔTn6212 for given conditions are equal and H1: means of expansion rates of Psa NZ13 and ΔTn6212 for given conditions are not equal.

| **Treatment** | **p value** |
|---|---|
| M9+20mM glucose | 0.968302 |
| M9+20mM glucose (10x inoc)* | 0.002232 |
| M9+20mM succinate pH 6.0* | 0.009191 |
| M9+20mM succinate pH 7.0* | 0.000043 |
| M9+CAA | 0.251976 |

The statistical analysis using a one-way ANOVA tested whether the expansion rate (Slope) for a particular genotype statistically differs between the treatments.

| **Genotype** | **p_value** |
|---|---|
| *Psa* NZ13* | 2.419240e-10 |
| *Psa* NZ13 ΔTn*6212* | 9.747942e-10 |

**Table S1. *P. syringae* ICEs. PsICEs isolated in this study.** * indicates the PsICE is non-redundant.

| ICE ID | Strain | non-redundant representative ICE | Country | Year | Isolated from | ICE integration locus | Tn*6212* | GenBank accession number | Original ICE name |
|---|---|---|---|---|---|---|---|---|---|
| ICE*Paf* 4394 * | *P. syringae* pv. *atrofaciens* ICMP 4394 | | New Zealand | 1968 | wheat | att-1 | ✓ | LJPO01000188.1; LJPO01000111.1 | |
| ICE*Paf* LMG5095 * | *P. syringae* pv. *atrofaciens* LMG5095 | | New Zealand | 1968 | wheat | att-1 | ✓ | CP028490 (coordinates: 3,496,500-3,600,924) | |
| ICE*Par* 4457 * | *P. coronafaciens* pv. *atropurpurea* ICMP4457 | | Japan | 1967 | ryegrass | att-2 | ✓ | LJPS01000162.1, LJPS01000123.1, LJPS01000111.1, LJPS01000149.1, LJPS01000047.1 | |
| ICE*Pat* ATCC11528-att1 * | *P. amygdali* pv. *tabaci* ATCC 11584 | | unkown | unkown | unkown | att-1 | ✗ | CP042804 (coordinates: 775,183-861,564) | |
| ICE*Pat* ATCC11528-att2 * | *P. amygdali* pv. *tabaci* ATCC 11584 | | unkown | unkown | unkown | att-2 | ✗ | CP042804 (coordinates: 1,612,730-1,720,434) | |
| ICE*Pav* 013 | *P. syrinage* pv. *avellanae* ISPaVe013 | ICE*Psa* NZ13 | Italy | 1991 | hazelnut | unkown | ✓ | AKCJ01000063.1, AKCJ01000059.1, AKCJ01000060.1, AKCJ01000061.1, AKCJ01000062.1 | |
| ICE*Pco* 1_6 * | *P. coronafaciens* pv. *oryzae* 1_6 | | Japan | unknown | rice | att-1 | ✓ | CP046035 (coordinates: 4,540,266-4,649,347) | |
| ICE*Pco* 19117 * | *P. congelans* ICMP 19117 | | Germany | 1994 | grass | att-2 | ✓ | LJQB01000076.1, LJQB01000035.1 | |
| ICE*Pco* DSM14939 | *P. congelans* DSM 14939 | ICE*Pco* 19117 | unknown | unknown | unknown | att-2 | ✓ | FNJH01000002.1 (coordinates: 211,040-320,216) | |
| ICE*Pco* LP205a | *P. congelans* LP205a | ICE*Ps* 91L8 | USA | 2001 | A. thaliana | unkown | ✓ | NQXX01000026.1, NQXX01000149.1 | |
| ICE*Pco* NL.P123 | *P. congelans* NL.P123 | ICE*Psy* 41A | USA | 2001 | A. thaliana | unkown | ✓ | NQYA01000023.1; NQYA01000134.1 | |
| ICE*Pdp* 529 | *P. syringae* pv. *delphinii* ICMP529 | ICE*Psa* NZ13 | New Zealand | 1957 | larkspur | att-1 | ✓ | LJQH01000365.1, LJQH01000290.1, LJQH01000355.1 | |
| ICE*Pfm* 18802 | *P. syringae* pv. *actinidifoliorum* ICMP18802 | ICE*Psa* NZ47-Cu | New Zealand | 2010 | kiwifruit | att-1 | ✗ | MUKM01000026.1 (coordinates: 61,728-152,968) | |
| ICE*Pfm* 19497 | *P. syringae* pv. *actinidifoliorum* ICMP 19497 | ICE*Psa* NZ64-Cu | New Zealand | 2010 | kiwifruit | att-1 | ✗ | LKBQ01000112.1 (coordinates: 22,274-149,483) | |
| ICE*Pfm* J207-1 * | *P. syringae* pv. *actinidifoliorum* J207-1 | | Japan | 2016 | kiwifruit | att-2 | ✓ | JAWHOS010000007 (coordinates 31,084-120,606) | |
| ICE*Pfm* J207-2 | *P. syringae* pv. *actinidifoliorum* J207-2 | ICE*Pfm* J207-1 | Japan | 2016 | kiwifruit | att-2 | ✓ | JAWHOR010000003 | |
| ICE*Pfm* J207-4 | *P. syringae* pv. *actinidifoliorum* J207-4 | ICE*Pfm* J207-1 | Japan | 2016 | kiwifruit | att-2 | ✓ | JAWHOQ010000024; JAWHOQ010000073 | |
| ICE*Pm* 11289 | *P. marginalis* ICMP 11289 | ICE*Psa* NZ47-Cu | New Zealand | 1991 | kiwifruit | att-1 | ✗ | LKGX01000080.1 (coordinates: 60,190-151,410) | |
| ICE*Ppa* 2367 * | *P. syringae* pv. *panici* LMG2367 | | USA | 1921-22 | proso millet | att-2 | ✓ | ALAC01000062.1, ALAC01000019.1 | |

| | | | | | | | | | |
|---|---|---|---|---|---|---|---|---|---|
| ICE*Pph* 1302A * | *P. syringae* pv. *phaseolicola* 1302A | | Ethiopia | 1994 | bean | att-2 | ✗ | AJ870974.1 (coordinates: 1,346-107,259) | PPHGI-1 |
| ICE*Ps* 11168 * | *P. syringae* ICMP 11168 | | New Zealand | 1991 | kiwifruit | att-2 | ✓ | LKGV01000001.1 (coordinates: 160,711-263,297) | |
| ICE*Ps* 243L2 | *P. syringae* 243L2 | ICE*Psa* C3 | China | 2014 | kiwifruit | att-1 | ✓ | JAWHPL010000014 (coordinates: 96,749-194,895) | |
| ICE*Ps* 247E2 | *P. syringae* 247E2 | ICE*Psa* C3 | China | 2014 | kiwifruit | att-1 | ✓ | JAWHPK010000011 (coordinates: 108,077-205,981) | |
| ICE*Ps* 248-6 * | *P. syringae* J248-6 | | Japan | 2016 | kiwifruit | att-1 | ✗ | JAWHOP010000009 (coordinates: 103,124-207,313) | |
| ICE*Ps* 254-4 * | *P. syringae* J254-4 | | Japan | 2016 | kiwifruit | att-1 | ✗ | JAWHOO010000007 (coordinates: 61,657-144,753) | |
| ICE*Ps* 26L6 | *P. syringae* 26L6 | ICE*Psa* C11 | China | 2014 | kiwifruit | att-1 | ✓ | JAWHPJ010000053 | |
| ICE*Ps* 2L4 * | *P. syringae* 2L4 | | China | 2014 | kiwifruit | att-2 | ✓ | JAWHPI010000026 (coordinates: 126,175-233,996) | |
| ICE*Ps* 304-1 * | *P. syringae* J304-1 | | Japan | 2016 | kiwifruit | att-1 | ✗ | JAWHNV010000009 (coordinates: 1-110,968) | |
| ICE*Ps* 309-1 * | *P. syringae* J309-1 | | Japan | 2016 | kiwifruit | att-3 | ✗ | JAWHNU010000008 (coordinates: 19,078-126,295) | |
| ICE*Ps* 31R1 * | *P. syringae* 31R1 | | unknown | unknown | unknown | att-2 | ✗ | LT629769.1 (coordinates: 398,653-516,816) | |
| ICE*Ps* 82L1 * | *P. syringae* 82L1 | | China | 2014 | kiwifruit | att-1 | ✓ | JAWHPE010000007 (coordinates: 125,239-225,777) | |
| ICE*Ps* 91L8 * | *P. syringae* 91L8 | | China | 2014 | kiwifruit | att-2 | ✓ | JAWHPD010000049; JAWHPD010000021 | |
| ICE*Psa* C1 | *P. syringae* pv. *actinidiae* C1 | ICE*Psa* NZ13 | China | 2010 | kiwifruit | att-1 | ✓ | KC148185.1 | |
| ICE*Psa* C11 * | *P. syringae* pv. *actinidiae* C11 | | China | 2012 | kiwifruit | att-2 | ✓ | MTHK01000001.1 (coordinates: 232,974-338,270) | |
| ICE*Psa* C15 * | *P. syringae* pv. *actinidiae* C15 | | China | 2012 | kiwifruit | att-2 | ✓ | MTHO01000001.1, MTHO01000028.1, MTHO01000264.1 | |
| ICE*Psa* C18 * | *P. syringae* pv. *actinidiae* C18 | | China | 2012 | kiwifruit | att-1 | ✓ | MTHR01000006.1 (coordinates: 32,416-130,045) | |
| ICE*Psa* C2 * | *P. syringae* pv. *actinidiae* C2 | | China | 2012 | kiwifruit | att-2 | ✓ | JAWHPM010000018; JAWHPM010000139; JAWHPM010000036; JAWHPM010000162 | |
| ICE*Psa* C24 | *P. syringae* pv. *actinidiae* C24 | ICE*Psa* C6 | China | 2014 | kiwifruit | att-2 | ✓ | MTHS01000003.1 (coordinates: 38,590-126,565) | |
| ICE*Psa* C26 | *P. syringae* pv. *actinidiae* C26 | ICE*Psa* C27 | China | 2014 | kiwifruit | att-2 | ✓ | MTHT01000001.1 (coordinates: 1,770-109,176) | |

| | | | | | | | | | |
|---|---|---|---|---|---|---|---|---|---|
| ICE*Psa* C27 * | *P. syringae* pv. *actinidiae* C27 | | China | 2014 | kiwifruit | att-2 | ✓ | MTHU01000001.1 (coordinates: 1-107,407) | |
| ICE*Psa* C28 | *P. syringae* pv. *actinidiae* C28 | ICE*Psa* C11 | China | 2014 | kiwifruit | att-2 | ✓ | MTHV01000001.1 (coordinates: 233,304-338,706) | |
| ICE*Psa* C29 | *P. syringae* pv. *actinidiae* C29 | ICE*Psa* I10 | China | 2014 | kiwifruit | att-2 | ✓ | MTHW01000005.1, MTHW01000045.1, MTHW01000257.1 | |
| ICE*Psa* C3 * | *P. syringae* pv. *actinidiae* C3 | | China | 2012 | kiwifruit | unknown | ✓ | MTCQ01000008.1 (coordinates: 1-97,777) | |
| ICE*Psa* C30 | *P. syringae* pv. *actinidiae* C30 | ICE*Psa* I10 | China | 2014 | kiwifruit | att-2 | ✓ | MTHX01000007.1, MTHX01000050.1, MTHX01000252.1 | |
| ICE*Psa* C31 | *P. syringae* pv. *actinidiae* C31 | ICE*Psa* I10 | China | 2014 | kiwifruit | att-2 | ✓ | MTHY01000007.1, MTHY01000050.1, MTHY01000250.1 | |
| ICE*Psa* C4 | *P. syringae* pv. *actinidiae* C4 | ICE*Psa* C2 | China | 2012 | kiwifruit | att-2 | ✓ | JAWHPH010000003 | |
| ICE*Psa* C5 | *P. syringae* pv. *actinidiae* C5 | ICE*Psa* I10 | China | 2012 | kiwifruit | att-2 | ✓ | JAWHPG010000018; JAWHPG010000148; JAWHPG010000001 | |
| ICE*Psa* C6 * | *P. syringae* pv. *actinidiae* C6 | | China | 2012 | kiwifruit | att-2 | ✓ | JAWHPF010000011; JAWHPF010000003 | |
| ICE*Psa* C62 | *P. syringae* pv. *actinidiae* C62 | ICE*Psa* C2 | China | 2014 | kiwifruit | att-1 | ✓ | MTIB01000003.1 (coordinates: 1,770-102,550) | |
| ICE*Psa* C66 * | *P. syringae* pv. *actinidiae* C66 | | China | 2012 | kiwifruit | att-1 | ✗ | MTYK01000003.1 (coordinates: 32,770-133,393) | |
| ICE*Psa* C67 | *P. syringae* pv. *actinidiae* C67 | ICE*Psa* C2 | China | 2012 | kiwifruit | att-2 | ✓ | MTYL01000003.1 (coordinates: 4-100,784) | |
| ICE*Psa* C68 | *P. syringae* pv. *actinidiae* C68 | ICE*Psa* C2 | China | 2014 | kiwifruit | att-1 | ✓ | MTYM01000003.1 (coordinates: 4-100,784) | |
| ICE*Psa* C69 | *P. syringae* pv. *actinidiae* C69 | ICE*Psa* C2 | China | 2014 | kiwifruit | att-1 | ✓ | MTYO01000003.1 (coordinates: 1,770-102,550) | |
| ICE*Psa* C70 | *P. syringae* pv. *actinidiae* C70 | ICE*Psa* C11 | China | 2014 | kiwifruit | att-2 | ✓ | MTYN01000001.1 (coordinates: 233,300-338,600) | |
| ICE*Psa* C73 | *P. syringae* pv. *actinidiae* C73 | ICE*Psa* C11 | China | 2014 | kiwifruit | att-2 | ✓ | MTYP01000001.1 (coordinates: 233,352-338,600) | |
| ICE*Psa* C9 | *P. syringae* pv. *actinidiae* C9 | ICE*Psa* I10 | China | 2010 | kiwifruit | att-2 | ✓ | CP032631.1 (coordinates: 1,753,593-1,862,396) | |
| ICE*Psa* CL1 | *P. syringae* pv. *actinidiae* CL1 | ICE*Psa* C2 | Chile | 2010 | kiwifruit | att-2 | ✓ | KC148188.1 | Andean island, Pac_ICE3 |
| ICE*Psa* CL2 | *P. syringae* pv. *actinidiae* CL2 | ICE*Psa* C2 | Chile | 2010 | kiwifruit | att-2 | ✓ | JAWHPC010000018; JAWHPC010000143; JAWHPC010000166; JAWHPC010000038 | |

| | | | | | | | | | |
|---|---|---|---|---|---|---|---|---|---|
| ICE*Psa* CL3 | *P. syringae* pv. *actinidiae* CL3 | ICE*Psa* C2 | Chile | 2010 | kiwifruit | att-2 | ✓ | JAWHPB010000002 (ccordinates: 1-100,772) | |
| ICE*Psa* CL4 | *P. syringae* pv. *actinidiae* CL4 | ICE*Psa* C2 | Chile | 2010 | kiwifruit | att-2 | ✓ | JAWHPA010000032; JAWHPA010000005 | |
| ICE*Psa* I10 * | *P. syringae* pv. *actinidiae* I10 | | Italy | 2008 | kiwifruit | att-2 | ✓ | ANGD01000014.1 (coordinates: 30,828-139,631) | Mediterranean island, Pac_ICE2 |
| ICEPsaI12.29 * | *P. syringae* pv. *actinidiae* CRAFRU12.29 | | Italy | 2011 | kiwifruit | att-2 | ✗ | CP019730.1 (coordinates: 1,737,475-1,829,979) | |
| ICE*Psa* I13 | *P. syringae* pv. *actinidiae* I13 | ICE*Psa* I10 | Italy | 2008 | kiwifruit | att-2 | ✓ | JAWHOZ010000001; JAWHOZ010000128 | |
| ICE*Psa* I4 | *P. syringae* pv. *actinidiae* I4 | ICE*Psa* I10 | Italy | 2008 | kiwifruit | att-2 | ✓ | JAWHOY010000015; JAWHOY010000159; JAWHOY010000162; JAWHOY010000035 | |
| ICE*Psa* I5 | *P. syringae* pv. *actinidiae* I5 | ICE*Psa* I10 | Italy | 2009 | kiwifruit | att-2 | ✓ | JAWHOX010000013; JAWHOX010000101; JAWHOX010000034 | |
| ICE*Psa* I6 | *P. syringae* pv. *actinidiae* I6 | ICE*Psa* I10 | Italy | 2009 | kiwifruit | att-2 | ✓ | JAWHOW010000001 (coordinates: 30,787-139,583) | |
| ICE*Psa* I7 | *P. syringae* pv. *actinidiae* I7 | ICE*Psa* I10 | Italy | 2009 | kiwifruit | att-1 | ✓ | JAWHOV010000002 (coordinates: 32,387-141,186) | |
| ICE*Psa* I8 | *P. syringae* pv. *actinidiae* I8 | ICE*Psa* I10 | Italy | 2010 | kiwifruit | att-2 | ✓ | JAWHOU010000002 (coordinates: 30,787-139,587) | |
| ICE*Psa* I9 | *P. syringae* pv. *actinidiae* I9 | ICE*Psa* I10 | Italy | 2010 | kiwifruit | att-2 | ✓ | JAWHOT010000002 (coordinates: 30,787-139,539) | |
| ICE*Psa* J263-2 | *P. syringae* pv. *actinidiae* J263-2 | ICE*Psa* NZ13 | Japan | 2016 | kiwifruit | att-1 | ✓ | JAWHON010000040; JAWHON010000010 | |
| ICE*Psa* J263-4 | *P. syringae* pv. *actinidiae* J263-4 | ICE*Psa* NZ13 | Japan | 2016 | kiwifruit | unknown | ✓ | JAWHOM010000009; JAWHOM010000129; JAWHOM010000184; JAWHOM010000173 | |
| ICE*Psa* J267-1 | *P. syringae* pv. *actinidiae* J267-1 | ICE*Psa* NZ13 | Japan | 2016 | kiwifruit | att-1 | ✓ | JAWHOL010000007 | |
| ICE*Psa* J267-2 | *P. syringae* pv. *actinidiae* J267-2 | ICE*Psa* NZ13 | Japan | 2016 | kiwifruit | unknown | ✓ | JAWHOK010000002 (coordinates: 1,104-101,724) | |
| ICE*Psa* J268-1 | *P. syringae* pv. *actinidiae* J268-1 | ICE*Psa* NZ13 | Japan | 2016 | kiwifruit | att-2 | ✓ | JAWHOJ010000004 | |
| ICE*Psa* J268-4 | *P. syringae* pv. *actinidiae* J268-4 | ICE*Psa* NZ13 | Japan | 2016 | kiwifruit | att-2 | ✓ | JAWHOI010000005; JAWHOI010000057 | |
| ICE*Psa* J268-6 | *P. syringae* pv. *actinidiae* J268-6 | ICE*Psa* NZ13 | Japan | 2016 | kiwifruit | att-1 | ✓ | JAWHOH010000002 (coordinates: 1,042-101,955) | |
| ICE*Psa* J269-6 | *P. syringae* pv. *actinidiae* J269-6 | ICE*Psa* NZ13 | Japan | 2016 | kiwifruit | att-2 | ✓ | JAWHOG010000006 | |

| | | | | | | | | | |
|---|---|---|---|---|---|---|---|---|---|
| ICE*Psa* J270-1 | *P. syringae* pv. *actinidiae* J270-1 | ICE*Psa* NZ13 | Japan | 2016 | kiwifruit | att-2 | ✓ | JAWHOF010000003 (coordinates: 30,809-131,660) | |
| ICE*Psa* J270-5 | *P. syringae* pv. *actinidiae* J270-5 | ICE*Psa* NZ13 | Japan | 2016 | kiwifruit | att-2 | ✓ | JAWHOD010000006 | |
| ICE*Psa* J271-1 | *P. syringae* pv. *actinidiae* J271-1 | ICE*Psa* NZ13 | Japan | 2016 | kiwifruit | unknown | ✓ | JAWHOC010000003 | |
| ICE*Psa* J271-3 | *P. syringae* pv. *actinidiae* J271-3 | ICE*Psa* NZ13 | Japan | 2016 | kiwifruit | att-2 | ✓ | JAWHOB010000002 (coordinates: 2: 30,809-131,661) | |
| ICE*Psa* J271-5 | *P. syringae* pv. *actinidiae* J271-5 | ICE*Psa* NZ13 | Japan | 2016 | kiwifruit | att-2 | ✓ | JAWHOA010000004; JAWHOA010000060 | |
| ICE*Psa* J272-5 | *P. syringae* pv. *actinidiae* J272-5 | ICE*Psa* NZ13 | Japan | 2016 | kiwifruit | att-2 | ✓ | JAWHNZ010000002 (coordinates: 30,809-131,661) | |
| ICE*Psa* J273-1 | *P. syringae* pv. *actinidiae* J273-1 | ICE*Psa* NZ13 | Japan | 2016 | kiwifruit | att-2 | ✓ | JAWHNY010000002 (coordinates: 30,909-131,760) | |
| ICE*Psa* J273-6 | *P. syringae* pv. *actinidiae* J273-6 | ICE*Psa* NZ13 | Japan | 2016 | kiwifruit | att-2 | ✓ | JAWHNX010000001 (coordinates: 26-100,925) | |
| ICE*Psa* J292-2 * | *P. syringae* pv. *actinidiae* J292-2 | | Japan | 2016 | kiwifruit | att-2 | ✗ | JAWHNW010000333; JAWHNW010000175; JAWHNW010000179; JAWHNW010000158; JAWHNW010000054 | |
| ICE*Psa* J38 | *P. syringae* pv. *actinidiae* J38 | ICE*Psa* NZ13 | Japan | 2014 | kiwifruit | att-2 | ✓ | MTYS01000003.1 (coordinates: 1,056-101,907) | |
| ICE*Psa* MAFF212063 * | *P. syringae* pv. *actinidiae* MAFF212063 | | Japan | 2012 | kiwifruit | att-2 | ✗ | CP024712 (coordinates: 4,717,464-4,816,692) | |
| ICE*Psa* NZ13 * | *P. syringae* pv. *actinidiae* NZ13 | | New Zealand | 2010 | kiwifruit | att-1 | ✓ | CP011972.2 (coordinates: 5,410,821-5,511,719) | Pacific Island, Pac_ICE1 |
| ICE*Psa* NZ31 | *P. syringae* pv. *actinidiae* NZ31 | ICE*Psa* NZ13 | New Zealand | 2010 | kiwifruit | unknown | ✓ | ANJD01000023.1, ANJD01000118.1, ANJD01000111.1 | |
| ICE*Psa* NZ32 | *P. syringae* pv. *actinidiae* NZ32 | ICE*Psa* NZ13 | New Zealand | 2010 | kiwifruit | att-1 | ✓ | ANJC01000006.1 (coordinates: 33,546-134,397) | |
| ICE*Psa* NZ33 | *P. syringae* pv. *actinidiae* NZ33 | ICE*Psa* NZ13 | New Zealand | 2011 | kiwifruit | att-1 | ✓ | ANJG01000046.1 (coordinates: 33,494-134,397) | |
| ICE*Psa* NZ34 | *P. syringae* pv. *actinidiae* NZ34 | ICE*Psa* NZ13 | New Zealand | 2011 | kiwifruit | att-1 | ✓ | ANJH01000079.1, ANJH01000040.1, ANJH01000162.1 | |
| ICE*Psa* NZ35 | *P. syringae* pv. *actinidiae* NZ35 | ICE*Psa* NZ13 | New Zealand | 2010 | kiwifruit | att-1 | ✓ | MTYX01000007.1 (coordinates: 354-101,113) | |
| ICE*Psa* NZ36 | *P. syringae* pv. *actinidiae* NZ36 | ICE*Psa* NZ13 | New Zealand | 2012 | kiwifruit | att-2 | ✓ | JAWHNT010000004 | |
| ICE*Psa* NZ37 | *P. syringae* pv. *actinidiae* NZ37 | ICE*Psa* NZ13 | New Zealand | 2010 | kiwifruit | att-1 | ✓ | MTYY01000003.1 (coordinates: 1,056-101,907) | |
| ICE*Psa* NZ38 | *P. syringae* pv. *actinidiae* NZ38 | ICE*Psa* NZ13 | New Zealand | 2014 | kiwifruit | att-2 | ✓ | MTYZ01000003.1 (coordinates: 170-101,073) | |

| | | | | | | | | | |
|---|---|---|---|---|---|---|---|---|---|
| ICE*Psa* NZ39 | *P. syringae* pv. *actinidiae* NZ39 | ICE*Psa* NZ13 | New Zealand | 2014 | kiwifruit | att-1 | ✓ | MTZA01000003.1 (coordinates: 93-100,944) | |
| ICE*Psa* NZ40 | *P. syringae* pv. *actinidiae* NZ40 | ICE*Psa* NZ13 | New Zealand | 2014 | kiwifruit | att-2 | ✓ | MTZB01000003.1 (coordinates: 170-101,073) | |
| ICE*Psa* NZ41 | *P. syringae* pv. *actinidiae* NZ41 | ICE*Psa* NZ13 | New Zealand | 2014 | kiwifruit | att-2 | ✓ | MTZC01000003.1 (coordinates: 170-101,021) | |
| ICE*Psa* NZ42 | *P. syringae* pv. *actinidiae* NZ42 | ICE*Psa* NZ13 | New Zealand | 2014 | kiwifruit | att-1 | ✓ | MTZD01000004.1 (coordinates: 759-101,610) | |
| ICE*Psa* NZ43 | *P. syringae* pv. *actinidiae* NZ43 | ICE*Psa* NZ13 | New Zealand | 2014 | kiwifruit | att-1 | ✓ | MTZE01000003.1 (coordinates: 26-100,877) | |
| ICE*Psa* NZ44 | *P. syringae* pv. *actinidiae* NZ44 | ICE*Psa* NZ13 | New Zealand | 2014 | kiwifruit | unknown | ✓ | JAWHNS010000003 (coordinates: 3: 1,056-101,955) | |
| ICE*Psa* NZ45 | *P. syringae* pv. *actinidiae* NZ45 | ICE*Psa* NZ13 | New Zealand | 2014 | kiwifruit | att-2 | ✗ | MTZF01000001.1 (coordinates: 1,733,917-1,834,820) | |
| ICE*Psa* NZ45-Cu * | *P. syringae* pv. *actinidiae* NZ45 | | New Zealand | 2014 | kiwifruit | att-1 | ✗ | MTZF01000001.1 (coordinates: 5,534,722-5,642,405) | $Psa_{NZ45}$ICE_Cu |
| ICE*Psa* NZ46 | *P. syringae* pv. *actinidiae* NZ46 | ICE*Psa* NZ13 | New Zealand | 2012 | kiwifruit | att-1 | ✓ | JAWHNR010000015; JAWHNR010000116 | |
| ICE*Psa* NZ47-Cu * | *P. syringae* pv. *actinidiae* NZ47 | | New Zealand | 2014 | kiwifruit | att-1 | ✗ | CP017009.1 (coordinates: 5,410,821-5,502,013) | $Psa_{NZ47}$ICE_Cu |
| ICE*Psa* NZ48 | *P. syringae* pv. *actinidiae* NZ48 | ICE*Psa* NZ13 | New Zealand | 2013 | kiwifruit | att-2 | ✓ | MTZH01000003.1 (coordinates: 26-100,929) | |
| ICE*Psa* NZ49 | *P. syringae* pv. *actinidiae* NZ49 | ICE*Psa* NZ13 | New Zealand | 2011 | kiwifruit | att-1 | ✓ | MTZJ01000003.1 (coordinates: 1,056-101,959) | |
| ICE*Psa* NZ62-Cu | *P. syringae* pv. *actinidiae* NZ62 | ICE*Psa* NZ47-Cu | New Zealand | 2015 | kiwifruit | att-2 | ✗ | MOMK01000003.1 (coordinates: 1,106-92,298) | |
| ICE*Psa* NZ63 | *P. syringae* pv. *actinidiae* NZ63 | ICE*Psa* NZ45-Cu | New Zealand | 2015 | kiwifruit | att-1 | ✗ | MOML01000002.1 (coordinates: 32,503-140,115) | |
| ICE*Psa* NZ64-Cu * | *P. syringae* pv. *actinidiae* NZ64 | | New Zealand | 2016 | kiwifruit | att-1 | ✗ | MOMM01000021.1, MOMM01000065.1 MOMM01000141.1 MOMM01000150.1 MOMM01000185.1 MOMM01000221.1 MOMM01000225.1, MOMM01000243.1 | $Psa_{NZ64}$ICE_Cu |
| ICE*Psa* P1 | *P. syringae* pv. *actinidiae* P1 | ICE*Psa* I10 | Portugal | 2010 | kiwifruit | att-2 | ✓ | JAWHNQ010000013; JAWHNQ010000203; JAWHNQ010000280; JAWHNQ010000007 | |
| ICE*Psa* P14.08 | *P. syringae* pv. *actinidiae* CRAFRU14.08 | ICEPsaI12.29 | Portugal | 2010 | kiwifruit | att-2 | ✗ | CP019732.1 (coordinates: 1,735,804-1,828,308) | |

| | | | | | | | | | |
|---|---|---|---|---|---|---|---|---|---|
| ICE*Psa* P155 * | *P. syringae* pv. *actinidiae* P155 | | unknown | 2018 | kiwifruit | att-2 | ✓ | CP032871 (coordinates 1,689,941-1,797,268) | |
| ICE*Psa* SK142-1 | *P. syringae* pv. *actinidiae* SK142-1 | ICE*Psa* NZ13 | South Korea | 2016 | kiwifruit | unknown | ✓ | JAWHNO010000008 | |
| ICE*Psa* SK145-1 | *P. syringae* pv. *actinidiae* SK145-1 | ICE*Psa* NZ13 | South Korea | 2016 | kiwifruit | unknown | ✓ | JAWHNN010000003 (coordinates: 1,056-101,955) | |
| ICE*Psa* SK146-1 | *P. syringae* pv. *actinidiae* SK146-1 | ICE*Psa* NZ13 | South Korea | 2016 | kiwifruit | unknown | ✓ | JAWHNM010000003 (ccordinates: 1,056-101,955) | |
| ICE*Psa* SK147-1 | *P. syringae* pv. *actinidiae* SK147-1 | ICE*Psa* NZ13 | South Korea | 2016 | kiwifruit | unknown | ✓ | JAWHNL010000003 (coordinates: 32,282-133,134) | |
| ICE*Psa* SK159-1 | *P. syringae* pv. *actinidiae* SK159-1 | ICE*Psa* C2 | South Korea | 2016 | kiwifruit | unknown | ✓ | JAWHNK010000009 | |
| ICE*Psa* SK160-1 | *P. syringae* pv. *actinidiae* SK160-1 | ICE*Psa* C2 | South Korea | 2016 | kiwifruit | att-1 | ✓ | JAWHNJ010000003 (coordinates: 1,059-101,867) | |
| ICE*Psa* SK161-1 | *P. syringae* pv. *actinidiae* SK161-1 | ICE*Psa* C2 | South Korea | 2016 | kiwifruit | unknown | ✓ | JAWHNI010000008 | |
| ICE*Psa* SK161-3 | *P. syringae* pv. *actinidiae* SK161-3 | ICE*Psa* C2 | South Korea | 2016 | kiwifruit | unknown | ✓ | JAWHNH010000005 | |
| ICE*Psa* SK162-1 | *P. syringae* pv. *actinidiae* SK162-1 | ICE*Psa* C2 | South Korea | 2016 | kiwifruit | att-1 | ✓ | JAWHNG010000008 | |
| ICE*Psa* SK162-2 | *P. syringae* pv. *actinidiae* SK162-2 | ICE*Psa* C2 | South Korea | 2016 | kiwifruit | unknown | ✓ | JAWHNF010000005 | |
| ICE*Psa* SK162-3 | *P. syringae* pv. *actinidiae* SK162-3 | ICE*Psa* C2 | South Korea | 2016 | kiwifruit | unknown | ✓ | JAWHNE010000006 | |
| ICE*Psa* SK164-1 * | *P. syringae* pv. *actinidiae* SK164-1 | | South Korea | 2016 | kiwifruit | unknown | ✓ | JAWHND010000002 (coordinates: 7,905-118,852) | |
| ICE*Psa* SK164-2 | *P. syringae* pv. *actinidiae* SK164-2 | ICE*Psa* SK164-1 | South Korea | 2016 | kiwifruit | unknown | ✓ | JAWHNC010000003 | |
| ICE*Psa* SK164-3 | *P. syringae* pv. *actinidiae* SK164-3 | ICE*Psa* SK164-1 | South Korea | 2016 | kiwifruit | unknown | ✓ | JAWHNB010000004 | |
| ICE*Psa* SK166-2 | *P. syringae* pv. *actinidiae* SK166-2 | ICE*Psa* NZ13 | South Korea | 2016 | kiwifruit | att-1 | ✓ | JAWHNA010000002 (coordinates: 32,409-133,261) | |
| ICE*Psa* SK167-1 | *P. syringae* pv. *actinidiae* SK167-1 | ICE*Psa* NZ13 | South Korea | 2016 | kiwifruit | unknown | ✓ | JAWHMZ010000001 (coordinates: 1,056-101,955) | |
| ICE*Psa* SK167-2 | *P. syringae* pv. *actinidiae* SK167-2 | ICE*Psa* NZ13 | South Korea | 2016 | kiwifruit | unknown | ✓ | JAWHMY010000002 | |
| ICE*Psa* SK167-3 | *P. syringae* pv. *actinidiae* SK167-3 | ICE*Psa* NZ13 | South Korea | 2016 | kiwifruit | att-1 | ✓ | JAWHMX010000005; JAWHMX010000187 | |
| ICE*Psa* SK168-1 | *P. syringae* pv. *actinidiae* SK168-1 | ICE*Psa* NZ13 | South Korea | 2016 | kiwifruit | att-1 | ✓ | JAWHMW010000003 (coordinates: 1,056-101,955) | |
| ICE*Psa* SK168-2 | *P. syringae* pv. *actinidiae* SK168-2 | ICE*Psa* NZ13 | South Korea | 2016 | kiwifruit | att-1 | ✓ | JAWHMV010000002 (coordinates: 93-100,992) | |

| | | | | | | | | | |
|---|---|---|---|---|---|---|---|---|---|
| ICE*Psa* SK168-3 | *P. syringae* pv. *actinidiae* SK168-3 | ICE*Psa* NZ13 | South Korea | 2016 | kiwifruit | att-2 | ✓ | JAWHMU010000003 (coordinates: 93-100,991) | |
| ICE*Psa* SK169-1 | *P. syringae* pv. *actinidiae* SK169-1 | ICE*Psa* NZ13 | South Korea | 2016 | kiwifruit | unknown | ✓ | JAWHMT010000003 (coordinates: 30,809-131,661) | |
| ICE*Psa* SK169-2 | *P. syringae* pv. *actinidiae* SK169-2 | ICE*Psa* NZ13 | South Korea | 2016 | kiwifruit | unknown | ✓ | JAWHMS010000004 | |
| ICE*Psa* SK169-3 | *P. syringae* pv. *actinidiae* SK169-3 | ICE*Psa* NZ13 | South Korea | 2016 | kiwifruit | unknown | ✓ | JAWHMR010000004 | |
| ICE*Psa* SK170-1 | *P. syringae* pv. *actinidiae* SK170-1 | ICE*Psa* NZ13 | South Korea | 2016 | kiwifruit | unknown | ✓ | JAWHMQ010000001 (coordinates: 32,400-133,299) | |
| ICE*Psa* SK170-2 | *P. syringae* pv. *actinidiae* SK170-2 | ICE*Psa* NZ13 | South Korea | 2016 | kiwifruit | att-1 | ✓ | JAWHMP010000011; JAWHMP010000034 | |
| ICE*Psa* SK170-3 | *P. syringae* pv. *actinidiae* SK170-3 | ICE*Psa* NZ13 | South Korea | 2016 | kiwifruit | unknown | ✓ | JAWHMO010000006 | |
| ICE*Psa* SK171-1 | *P. syringae* pv. *actinidiae* SK171-1 | ICE*Psa* NZ13 | South Korea | 2016 | kiwifruit | unknown | ✓ | JAWHMN010000008 | |
| ICE*Psa* SK171-2 | *P. syringae* pv. *actinidiae* SK171-2 | ICE*Psa* NZ13 | South Korea | 2016 | kiwifruit | unknown | ✓ | JAWHMM010000002 (coordinates: 1,056-101,954) | |
| ICE*Psa* SK171-3 | *P. syringae* pv. *actinidiae* SK171-3 | ICE*Psa* NZ13 | South Korea | 2016 | kiwifruit | att-1 | ✓ | JAWHML010000003 (coordinates: 3: 32,409-133,260) | |
| ICE*Psa* SK172-1 | *P. syringae* pv. *actinidiae* SK172-1 | ICE*Psa* NZ13 | South Korea | 2016 | kiwifruit | unknown | ✓ | JAWHMK010000002 (coordinates: 1,056-101,955) | |
| ICE*Psa* SK172-2 | *P. syringae* pv. *actinidiae* SK172-2 | ICE*Psa* NZ13 | South Korea | 2016 | kiwifruit | unknown | ✓ | JAWHMJ010000002 (coordinates: 32,282-133,134) | |
| ICE*Psa* SK173-1 | *P. syringae* pv. *actinidiae* SK173-1 | ICE*Psa* NZ13 | South Korea | 2016 | kiwifruit | att-1 | ✓ | JAWHMI010000003 | |
| ICE*Psa* SK173-2 | *P. syringae* pv. *actinidiae* SK173-2 | ICE*Psa* NZ13 | South Korea | 2016 | kiwifruit | unknown | ✓ | JAWHMH010000004 | |
| ICE*Psa* SK173-3 | *P. syringae* pv. *actinidiae* SK173-3 | ICE*Psa* NZ13 | South Korea | 2016 | kiwifruit | att-1 | ✓ | JAWHMG010000005 | |
| ICE*Psa* SK182-2 | *P. syringae* pv. *actinidiae* SK182-2 | ICE*Psa* NZ13 | South Korea | 2016 | kiwifruit | unknown | ✓ | JAWHMF010000005 | |
| ICE*Psa* SK189-1 | *P. syringae* pv. *actinidiae* SK189-1 | ICE*Psa* NZ13 | South Korea | 2016 | kiwifruit | att-1 | ✓ | JAWHME010000008 | |
| ICE*Psa* SK189-2 | *P. syringae* pv. *actinidiae* SK189-2 | ICE*Psa* NZ13 | South Korea | 2016 | kiwifruit | att-1 | ✓ | JAWHMD010000001 (coordinates: 32,400-133,251) | |
| ICE*Psa* SK190-1 | *P. syringae* pv. *actinidiae* SK190-1 | ICE*Psa* NZ13 | South Korea | 2016 | kiwifruit | unknown | ✓ | JAWHMC010000008 | |
| ICE*Psa* SK190-2 | *P. syringae* pv. *actinidiae* SK190-2 | ICE*Psa* NZ13 | South Korea | 2016 | kiwifruit | unknown | ✓ | JAWHMB010000002 (coordinates: 32,400-133,299) | |
| ICE*Psa* SK190-3 | *P. syringae* pv. *actinidiae* SK190-3 | ICE*Psa* NZ13 | South Korea | 2016 | kiwifruit | att-2 | ✓ | JAWHMA010000004 | |

| | | | | | | | | | | |
|---|---|---|---|---|---|---|---|---|---|---|
| ICE*Psa* SK191-1 | *P. syringae* pv. *actinidiae* SK191-1 | ICE*Psa* NZ13 | South Korea | 2016 | kiwifruit | att-1 | ✓ | JAWHLZ010000001 (coordinates: 32,282-133,134) | |
| ICE*Psa* SK191-2 | *P. syringae* pv. *actinidiae* SK191-2 | ICE*Psa* NZ13 | South Korea | 2016 | kiwifruit | unknown | ✓ | JAWHLY010000003 | |
| ICE*Psa* SK191-3 | *P. syringae* pv. *actinidiae* SK191-3 | ICE*Psa* NZ13 | South Korea | 2016 | kiwifruit | unknown | ✓ | JAWHLX010000003 (coordinates: 32,282-133,134) | |
| ICE*Psa* SK192-1 | *P. syringae* pv. *actinidiae* SK192-1 | ICE*Psa* NZ13 | South Korea | 2016 | kiwifruit | unknown | ✓ | JAWHLW010000008 | |
| ICEPsaSK192-1S | *P. syringae* pv. *actinidiae* SK192-1S | ICE*Psa* NZ13 | South Korea | 2016 | kiwifruit | unknown | ✓ | JAWHLV010000003 (coordinates: 33,468-134-320) | |
| ICE*Psa* SK192-2 | *P. syringae* pv. *actinidiae* SK192-2 | ICE*Psa* NZ13 | South Korea | 2016 | kiwifruit | att-1 | ✓ | JAWHLU010000001 (coordinates: 1,089-101,683) | |
| ICE*Psa* SK197-R1 | *P. syringae* pv. *actinidiae* SK197-R1 | ICE*Psa* NZ13 | South Korea | 2016 | kiwifruit | att-1 | ✓ | JAWHLT010000003 (coordinates: 33,468-134,320) | |
| ICE*Psa* SK197-R2 | *P. syringae* pv. *actinidiae* SK197-R2 | ICE*Psa* NZ13 | South Korea | 2016 | kiwifruit | unknown | ✓ | JAWHLS010000005 | |
| ICE*Psa* SK197-R3 | *P. syringae* pv. *actinidiae* SK197-R3 | ICE*Psa* NZ13 | South Korea | 2016 | kiwifruit | unknown | ✓ | JAWHLR010000006 | |
| ICE*Psa* SK198-1 | *P. syringae* pv. *actinidiae* SK198-1 | ICE*Psa* NZ13 | South Korea | 2016 | kiwifruit | unknown | ✓ | JAWHLQ010000003 (coordinates: 1,056-101,955) | |
| ICE*Psa* SK198-2 | *P. syringae* pv. *actinidiae* SK198-2 | ICE*Psa* NZ13 | South Korea | 2016 | kiwifruit | unknown | ✓ | JAWHLP010000003 (coordinates: 32,400-133,299) | |
| ICE*Psa* SK198-3 | *P. syringae* pv. *actinidiae* SK198-3 | ICE*Psa* NZ13 | South Korea | 2016 | kiwifruit | att-1 | ✓ | JAWHLO010000002 (coordinates: 1,056-101,954) | |
| ICE*Psa* SK199-1 | *P. syringae* pv. *actinidiae* SK199-1 | ICE*Psa* NZ13 | South Korea | 2016 | kiwifruit | unknown | ✓ | JAWHLN010000003 (coordinates: 1,056-101,955) | |
| ICE*Psa* SK199-3 | *P. syringae* pv. *actinidiae* SK199-3 | ICE*Psa* NZ13 | South Korea | 2016 | kiwifruit | att-1 | ✓ | JAWHLM010000003 (coordinates: 93--100,794) | |
| ICE*Psa* SK200-1 | *P. syringae* pv. *actinidiae* SK200-1 | ICE*Psa* NZ13 | South Korea | 2016 | kiwifruit | unknown | ✓ | JAWHLL010000008 | |
| ICE*Psa* SK200-R2 | *P. syringae* pv. *actinidiae* SK200-R2 | ICE*Psa* NZ13 | South Korea | 2016 | kiwifruit | att-2 | ✓ | JAWHLK010000001 (coordinates: 1,056-101,955) | |
| ICE*Psa* SK200-R3 | *P. syringae* pv. *actinidiae* SK200-R3 | ICE*Psa* NZ13 | South Korea | 2016 | kiwifruit | att-1 | ✓ | JAWHLJ010000002 | |
| ICE*Psa* SK41-8 | *P. syringae* pv. *actinidiae* SK41-8 | ICE*Psa* NZ13 | South Korea | 2016 | kiwifruit | att-1 | ✓ | JAWHLI010000008 | |
| ICE*Psa* SK42-1 | *P. syringae* pv. *actinidiae* SK42-1 | ICE*Psa* NZ13 | South Korea | 2016 | kiwifruit | unknown | ✓ | JAWHLH010000003 (coordinates: 32,527-132,999) | |
| ICE*Psa* SK44-1 | *P. syringae* pv. *actinidiae* SK44-1 | ICE*Psa* NZ13 | South Korea | 2016 | kiwifruit | unknown | ✓ | JAWHLG010000007 | |
| ICE*Psa* SK44-5 | *P. syringae* pv. *actinidiae* SK44-5 | ICE*Psa* NZ13 | South Korea | 2016 | kiwifruit | unknown | ✓ | JAWHLF010000001 (coordinates: 30,809-131,661) | |

| | | | | | | | | | |
|---|---|---|---|---|---|---|---|---|---|
| ICE*Psa* SK44-8 | *P. syringae* pv. *actinidiae* SK44-8 | ICE*Psa* NZ13 | South Korea | 2016 | kiwifruit | unknown | ✓ | JAWHLE010000003 | |
| ICE*Psa* SK45-1 | *P. syringae* pv. *actinidiae* SK45-1 | ICE*Psa* NZ13 | South Korea | 2016 | kiwifruit | att-2 | ✓ | JAWHLD010000003 (coordinates: 30,909-131,808) | |
| ICE*Psa* SK47-1 | *P. syringae* pv. *actinidiae* SK47-1 | ICE*Psa* NZ13 | South Korea | 2016 | kiwifruit | unknown | ✓ | JAWHLC010000003 (coordinates: 1,056-101,723) | |
| ICE*Psa* SK57-1 | *P. syringae* pv. *actinidiae* SK57-1 | ICE*Psa* NZ13 | South Korea | 2016 | kiwifruit | att-1 | ✓ | JAWHLB010000008 | |
| ICE*Psa* SK58-6 | *P. syringae* pv. *actinidiae* SK58-6 | ICE*Psa* NZ13 | South Korea | 2016 | kiwifruit | unknown | ✓ | JAWHLA010000008 | |
| ICE*Psa* SK60-3 | *P. syringae* pv. *actinidiae* SK60-3 | ICE*Psa* NZ13 | South Korea | 2016 | kiwifruit | unknown | ✓ | JAWHKZ010000008 | |
| ICE*Psa* SK60-4 | *P. syringae* pv. *actinidiae* SK60-4 | ICE*Psa* NZ13 | South Korea | 2016 | kiwifruit | unknown | ✓ | JAWHKY010000005 | |
| ICE*PsaS* K7 | *P. syringae* pv. *actinidiae* K7 | ICE*Psa* I10 | South Korea | 2014 | kiwifruit | att-2 | ✓ | MTYW01000054.1, MTYW01000001.1 | |
| ICE*Ps* BRIP34876 | *P. syringae* BRIP34876 | ICE*Ps* BRIP34881 | Australia | 1971 | barley | att-2 | ✓ | AMXK01000055.1, AMXK01000099.1, AMXK01000014.1 | |
| ICE*Ps* BRIP34881 * | *P. syringae* BRIP34881 | | Australia | 1971 | barley | att-2 | ✓ | AMXL01000033.1, AMXL01000031.1 | |
| ICE*Ps* BRIP39023 * | *P. syringae* BRIP39023 | | Australia | 1988 | wheat | att-1 | ✗ | AMZX01000047.1, AMZX01000001.1 | |
| ICE*Ps* BS0292 * | *P. syringae* BS0292 | | unknown | unknown | unknown | att-2 | ✗ | FOVV01000006.1 (coordinates: 158,328-242,982) | |
| ICE*Ps* BS3827 * | *P. syringae* BS3827 | | unknown | unknown | unknown | att-2 | ✓ | FOQB01000001.1 (coordinates: 159,057-259,124) | |
| ICE*Ps* BS3829 * | *P. syringae* BS3829 | | unknown | unknown | unknown | att-2 | ✓ | FOPR01000002.1 (coordinates: 135,875-241,175) | |
| ICE*Ps* KCTC12500 | *P. syringae* KCTC12500 | ICE*Psy* 3023 | unknown | unknown | unknown | att-1 | ✗ | AYTM02000002.1 (coordinates: 3,253,758-3,390,606) | |
| ICE*Ps* KN2.a.3 * | *P. syringae* KN2.a.3 | | USA | 2000-14 | A. thaliana | unknown | ✓ | NHTA01000027.1; NHTA01000006.1; NHTA01000049.1 | |
| ICE*Ps* LMC.P91 * | *P. syringae* LMC.P91 | | USA | 2000-14 | A. thaliana | unknown | ✓ | NHTB01000006.1, NHTB01000127 | |
| ICE*Ps* Pengzhou8 * | *P. syringae* Pengzhou8 | | China | 2014 | kiwifruit | att-1 | ✓ | JAWHNP010000354 (coordinates: 126,432-230,024) | |
| ICE*Ps* Ps25 | *P. syringae* Ps25 | ICE*Pst* B13-200 | USA | 2016 | tomato | att-2 | ✗ | CP034558 (coordinates: 4,985,890-5,082,721) | |
| ICE*Pst* B13-200 * | *P. syringae* pv. *tomato* B13-200 | | Canada | 2013 | tomato | att-2 | ✗ | CP019871 (coordinates: 1,832,845-1,929,680) | |
| ICE*Psy* 2340 * | *P. syringae* pv. *syringae* 2340 | | Hungary | 1985 | pear | att-1 | ✓ | LIHT01000079.1 (coordinates: 110,882-208,477) | |
| ICE*Psy* 3023 * | *P. syringae* pv. *syringae* ICMP3023 | | UK | NA | bean | att-1 | ✗ | LJRK01000049.1 (coordinates: 340-137,148) | |

| | | | | | | | | | |
|---|---|---|---|---|---|---|---|---|---|
| ICE*Psy* 41A * | *P. syringae* pv. *syringae* 41A | | France | 2011 | apricot | att-2 | ✓ | JYHJ01000001.1 (coordinates: 860,840-963,244) | |
| ICE*Psy* B728a * | *P. syringae* pv. *syringae* B728a | | USA | 1985 | bean | att-2 | ✓ | CP000075.1 (coordinates: 1,604,398-1,724,178) | GI-7 |
| ICE*Psy* HS191 * | *P. syringae* pv. *syringae* HS191 | | Australia | 1969 | millet | att-1 | ✗ | CP006256.1 (coordinates: 5,140,380-5,226,421) | |
| ICE*Psy* NZIPFR-PS7 * | *P. syringae* pv. *syringae* NZIPFR-PS7 | | New Zealand | 2010 | cherry | att-2 | ✓ | LKCE01000012.1 (coordinates: 148,068-254,151) | |
| ICE*Psy* SM * | *P. syringae* pv. *syringae* SM | | USA | N/A | wheat | att-2 | ✗ | APWT01000010.1; APWT01000011.1 | |
| ICE*Pvir* CDRTc14 * | *P. viridiflava* CDRTc14 | | Austria | 2013 | whitetop (root) | att-2 | ✗ | MBPF01000022.1 (coordinates: 319,987-414,369) | |

**Table S2.** ***P. aeruginosa* ICEs.** * indicates the ICE is integrated in tandem with another ICE. Amino acid % identity indicated the % identity between the DEAD-BOX helicase of the element with the DEAD-BOX helicase of ICE*Psa* NZ13.

| Name | Original name | Strain ID | Isolation | Gene Bank accession | ICE family | *att* site | amino acid % identity |
|---|---|---|---|---|---|---|---|
| P chlororaphis PA23 | | *P. chlororaphis* strain PA23 | soybean root | CP008696.1 | NA | *att-2* | 86% |
| P mosselii SJ10 | | *P. mosselii* SJ10 | wastewater | CP009365.1 | NA | *att-2* | 81% |
| Pa B10W | | *P. aeruginosa* isolate B10W | wastewater | CP017969.1 | pKLC102/PAGI-2 | *att-2* | 82% |
| Pa B136-33 | | *P. aeruginosa* B136-33 | human | CP004061.1 | pKLC102/PAGI-2 | *att-1* | 83% |
| pKLC102 | pKLC102 | *P. aeruginosa* strain C | human | AY257538.1 | pKLC102/PAGI-2 | NA | 82% |
| Pa FA-HZ1 | | *P. aeruginosa* strain FA-HZ1 | wastewater | CP017353 | pKLC102/PAGI-2 | *att-2* | 83% |
| Pa MTB-1 | | *P. aeruginosa* MTB-1 | soil | CP006853.1 | pKLC102/PAGI-2 | *att-1* | 82% |
| Pa NCGM2.S1 | | *P. aeruginosa* NCGM2.S1 | human | AP012280.1 | NA | *att-2* | 82% |
| Pa PA1 | | *P. aeruginosa* PA1 | human | CP004054.2 | pKLC102/PAGI-2 | *att-2* | 82% |
| PAPI-1 | PAPI-1 | *P. aeruginosa* PA14 | human | AY273869.1 | pKLC102/PAGI-2 | *att-1* | 81% |
| Pa PA14Or | | *P. aeruginosa* isolate PA14Or | NA | LT608330.1 | pKLC102/PAGI-2 | *att-1* | 81% |
| Pa PA1R | | *P. aeruginosa* PA1R | NA | CP004055.1 | pKLC102/PAGI-2 | *att-2* | 82% |
| Pa PA38182 | | *P. aeruginosa* PA38182 | human | HG530068.1 | NA | NA | 83% |
| Pa PA7 | GI PA7 | *P. aeruginosa* PA7 | human | CP000744.1 | pKLC102/PAGI-2 | *att-2* | 83% |
| Pa PA96 | | *P. aeruginosa* PA96 | human | CP007224.1 | pKLC102/PAGI-2 | *att-2* | 82% |
| PAGI-5 | PAGI-5 | *P. aeruginosa* PSE9 | NA | EF611301.1 | pKLC102/PAGI-2 | NA | 83% |
| Pa SGVI ST111 | ICE1 | *P. aeruginosa* SGVI ST111 | human | KT887560.1 | pKLC102/PAGI-2 | NA | 82% |
| Pa 8380 | | *P. aeruginosa* strain 8380 | human | AP014839.2 | pKLC102/PAGI-2 | *att-1* | 82% |
| Pa BAMCPA07-48 | | *P. aeruginosa* strain BAMCPA07-48 | human | CP015377.1 | pKLC102/PAGI-2 | *att-1* | 82% |
| Pa Carb01 63 I | | *P. aeruginosa* strain Carb01 63 | human | CP011317.1 | pKLC102/PAGI-2 | *att-1* | 83% |
| Pa Carb01 63 II | | *P. aeruginosa* strain Carb01 63 | human | CP011317.1 | pKLC102/PAGI-2 | *att-1* | 83% |
| Pa DN1 | | *P. aeruginosa* strain DN1 | soil | CP017099.1 | NA | *att-2* | 81% |
| Pa F23197 | | *P. aeruginosa* strain F23197 | human | CP008856.1 | pKLC102/PAGI-2 | *att-2* | 82% |
| Pa F30658 II | | *P. aeruginosa* strain F30658 | human | CP008857.1 | pKLC102/PAGI-2 | *att-2* * | 82% |
| Pa F30658 I | | *P. aeruginosa* strain F30658 | human | CP008857.1 | NA | *att-1* | 82% |
| Pa F63912 | | *P. aeruginosa* strain F63912 | human | CP008858.1 | pKLC102/PAGI-2 | *att-1* | 82% |
| Pa H27930 | | *P. aeruginosa* strain H27930 | human | CP008860.1 | pKLC102/PAGI-2 | *att-2* * | 82% |
| Pa NCGM 1900 | | *P. aeruginosa* strain NCGM 1990 | human | AP014622.1 | NA | *att-2* | 82% |

| | | | | | | | |
|---|---|---|---|---|---|---|---|
| Pa NCGM 1984 | | *P. aeruginosa* strain NCGM 1984 | human | AP014646.1 | NA | *att-2* | 82% |
| Pa NCGM257 | | *P. aeruginosa* strain NCGM257 | human | AP014651 | pKLC102/PAGI-2 | *att-1* | 82% |
| Pa PA1RG | | *P. aeruginosa* strain PA1RG | hospital sewage | CP012679.1 | pKLC102/PAGI-2 | *att-2* | 82% |
| Pa PSE305 | | *P. aeruginosa* strain PSE305 | sheep | HG974234.1 | pKLC102/PAGI-2 | *att-1* | 82% |
| Pa S04 90 I | | *P. aeruginosa* strain S04 90 | human | CP011369.1 | pKLC102/PAGI-2 | *att-1* | 82% |
| Pa S04 90 II | | *P. aeruginosa* strain S04 90 | human | CP011369.1 | NA | *att-2* | 82% |
| Pa S86968 II | | *P. aeruginosa* strain S86968 | human | CP008865.1 | pKLC102/PAGI-2 | *att-2* | 82% |
| Pa S86968 I | | *P. aeruginosa* strain S86968 | human | CP008865.1 | pKLC102/PAGI-2 | *att-1* | 83% |
| Pa T63266 | | *P. aeruginosa* strain T63266 | human | CP008868.1 | pKLC102/PAGI-2 | *att-2* | 82% |
| Pa W16407 | | *P. aeruginosa* strain W16407 | human | CP008869.1 | pKLC102/PAGI-2 | *att-1* | 82% |
| Pa UCBPP-PA14 | PAPI-1 | *P. aeruginosa* UCBPP-PA14 | human | CP000438.1 | pKLC102/PAGI-2 | *att-1* | 81% |
| Pa VRFPA04 | | *P. aeruginosa* VRFPA04 | human | CP008739.2 | NA | *att-2* | 83% |
| PA W45909 | | *P. aeruginosa* strain W45909 | human | CP008871.1 | pKLC102/PAGI-2 | *att-2* | 83% |
| PA YL84 | | *P. aeruginosa* YL84 | compost | CP007147 | pKLC102/PAGI-2 | *att-2* | 82% |
| Pf-5 PAPI | PFGI-1 | *P. protegens* Pf-5 | rizhosphere | CP000076.1 | pKLC102/PAGI-2 | *att-2* | 83% |
| P. sp. WCS374 | | *P* . sp. WCS374 | rhizosphere | CP007638.1 | NA | *att-1* | 83% |

**Table S3. PsICE backbone genes.**

| Number | Name | locus tag for *Psa* NZ13 |
|---|---|---|
| 1 | *parA* – chromosome partitioning ATPase | IYO_024345 |
| 2 | hypothetical protein | IYO_024350 |
| 3 | hypothetical protein - DUF2786 | IYO_024355 |
| 4 | *dnaB* – replicative DNA helicase | IYO_024360 |
| 5 | hypothetical protein | |
| 6 | hypothetical protein | |
| 7 | hypothetical protein | |
| 8 | *parB* – chromosome partitioning ATPase | IYO_024385 |
| 9 | hypothetical protein - DUF2857 | |
| 10 | hypothetical protein | |
| | CR1 | |
| 11 | hypothetical protein - DUF1845 | |
| 12 | hypothetical protein - DUF3158 | |
| 13 | *ssb* – single-stranded DNA-binding protein | IYO_024415 |
| | CR2 | |
| 14 | helicase | IYO_024430 |
| | CR3 | |
| 15 | *pilL* – conjugative transfer protein | IYO_024440 |
| 16 | *pilN* – conjugative transfer protein | IYO_024445 |
| 17 | *pilO* – conjugative transfer protein | IYO_024450 |
| 18 | *pilP* – conjugative transfer protein | IYO_024455 |
| 19 | *pilQ* – conjugative transfer protein | IYO_024460 |
| 20 | *pilR* – conjugative transfer protein | IYO_024465 |
| 21 | *pilS* – conjugative transfer protein | IYO_024470 |
| 22 | *pilU* – conjugative transfer protein | IYO_024475 |
| 23 | *pilV* – conjugative transfer protein | IYO_024480 |
| 24 | *pilM* – conjugative transfer protein | IYO_024485 |
| | CR4 | |
| 25 | *topIII* – topoisomerase III | IYO_024590 |
| 26 | *tonB* – periplasmatic protein now DUF3577 | |
| 27 | hypothetical protein - DUF3275 | |
| 28 | hypothetical protein | |
| 29 | hypothetical protein | |
| 30 | hypothetical protein | |
| 31 | hypothetical protein | |
| 32 | methylase – plasmid related protein | IYO_024640 |
| 33 | DEAD box helicase –superfamily II DNA/RNA helicase | IYO_024645 |
| | CR5 | |
| 34 | MCP – Methyl-accepting chemotaxis protein | |
| 35 | ICE pep – probable exported protein | IYO_024660 |
| 36 | lytic transglycosylase – Soluble lytic murein transglyco | IYO_024665 |
| 37 | hypothetical protein - DUF2859 | IYO_024670 |
| 38 | *traD* – TC4P conjugative coupling factor | IYO_024675 |
| 39 | hypothetical protein – DUF4400 | IYO_024680 |
| 40 | *uvrD* – helicase | IYO_024685 |
| | CR6 | |
| 41 | hypothetical protein | |
| 42 | hypothetical protein | |

| | | |
|---|---|---|
| 43 | hypothetical protein – DUF3262 | IYO_024710 |
| 44 | hypothetical protein – DUF2976 | IYO_024715 |
| 45 | hypothetical protein – DUF3487 | IYO_024720 |
| 46 | hypothetical protein – DUF2895 | IYO_024725 |
| 47 | hypothetical protein – traK superfamily | IYO_024730 |
| 48 | hypothetical protein – virB10 like domain | IYO_024735 |
| 49 | hypothetical protein | IYO_024740 |
| 50 | *traC* – conjugal transfer protein (virB4) | IYO_024745 |
| 51 | hypothetical protein | IYO_024750 |
| 52 | DSBA – protein-disulfite isomerase | IYO_024755 |
| | CR7 | |
| 53 | hypothetical protein – DUF1525 | IYO_024835 |
| 54 | *traU* – conjugal transfer protein | IYO_024840 |
| 55 | hypothetical protein | IYO_024845 |
| 56 | hypothetical protein | IYO_024850 |
| 57 | *traG* – conjugal transfer protein | IYO_024855 |
| | CR8 | |
| 58 | *rulB* – error-prone, lesion bypass DNA polymerase | IYO_024865 |
| 59 | *rulA* – error-prone repair protein | IYO_024870 |
| 60 | hypothetical protein | IYO_024875 |
| | CR9 | |
| 61 | *traI* – relaxase | IYO_024880 |
| 62 | *int* – integrase | IYO_024885 |

**Table S4. Alfy homology detection.** The % homology indicates the % of the length of the "subject ICE" sequence that has strong homologies with the "Homologous ICE".

| Subject ICE | Homologous ICE | % length of homology |
|---|---|---|
| ICEPar4457 | ICEPsaNZ13 | 95.1 |
| ICEPs11168 | ICEPsBS3827 | 22.8 |
| ICEPs11168 | ICEPs82L1 | 16.5 |
| ICEPs11168 | ICEPsy41A01 | 9.7 |
| ICEPaf4394 | ICEPsPengzhou8 | 40.5 |
| ICEPaf4394 | ICEPsatLMG2095 | 37 |
| ICEPaf4394 | ICEPsaNZ13, ICEPsaC18, ICEPsBRIP34881, ICEPs82L1, ICEPar4457 | 7 |
| ICEPatATCC11528_2 | PatATCC11528_1 | 21.5 |
| ICEPatATCC11528_2 | ICEPsa292-2 | 17.9 |
| ICEPatATCC11528_2 | ICEPs254-4 | 13.9 |
| ICEPatATCC11528_2 | ICEPs304-1 | 12.1 |
| ICEPatATCC11528_2 | ICEPsaNZ64_Cu | 5.5 |
| ICEPatATCC11528_2 | ICEPsaC66 | 5.3 |
| ICEPatATCC11528_2 | ICEPs2L4 | 5 |
| ICEPco1_6 | ICEPsatLMG2095 | 39.1 |
| ICEPco1_6 | ICEPs82L1 | 8.3 |
| ICEPco19117 | ICEPsy41A01 | 42.2 |
| ICEPco19117 | ICEPsBS3827 | 7.6 |
| ICEPco19117 | ICEPco1_6 | 5.2 |
| ICEPfm207-1 | ICEPsaMAFF212063 | 59.4 |
| ICEPfm207-1 | ICEPstB13-200 | 28.6 |
| ICEPpaLMG2367 | ICEPsaNZ45_Cu | 49.2 |
| ICEPpaLMG2367 | ICEPsyB728a, ICEPs164-1 | 19.3 |
| ICEPpaLMG2367 | ICEPsyB728a, ICEPsaNZ45_Cu, ICEPsaC15, ICEPs164-1 | 13.3 |
| ICEPpaLMG2367 | ICEPsyB728a, ICEPsaP155, ICEPsaNZ45_Cu, ICEPsaC15, ICEPs164-1 | 6.1 |
| ICEPph1302A | ICEPsyHS191 | 15.3 |
| ICEPph1302A | ICEPsy2340 | 9.8 |
| ICEPph1302A | ICEPsBRIP39023 | 6.6 |
| ICEPph1302A | ICEPsKN2.a.3 | 5.5 |
| ICEPs164-1 | ICEPsaNZ45_Cu | 44.1 |
| ICEPs164-1 | ICEPsaC15 | 10.6 |
| ICEPs164-1 | ICEPsaC3 | 8.6 |
| ICEPs164-1 | ICEPsyHS191 | 5 |
| ICEPs248-6 | PatATCC11528_1 | 25.6 |
| ICEPs248-6 | ICEPs304-1 | 17.6 |
| ICEPs248-6 | ICEPs254-4 | 17.3 |
| ICEPs248-6 | ICEPatATCC11528_2 | 7.5 |
| ICEPs248-6 | ICEPsa292-2 | 6.7 |
| ICEPs248-6 | ICEPsaC66 | 5.2 |
| ICEPs254-4 | ICEPs304-1 | 19.7 |
| ICEPs254-4 | ICEPatATCC11528_2 | 17.4 |
| ICEPs254-4 | ICEPs248-6 | 17 |
| ICEPs254-4 | PatATCC11528_1 | 7.8 |
| ICEPs254-4 | ICEPsa292-2 | 6.7 |

| | | |
|---|---|---|
| ICEPs2L4 | ICEPsaNZ64_Cu | 23.9 |
| ICEPs2L4 | ICEPsaC66 | 20.9 |
| ICEPs2L4 | ICEPsaNZ13, ICEPsBRIP34881, ICEPar4457 | 15.7 |
| ICEPs2L4 | ICEPatATCC11528_2 | 6.8 |
| ICEPs304-1 | ICEPs248-6 | 21.1 |
| ICEPs304-1 | ICEPs254-4 | 16 |
| ICEPs304-1 | PatATCC11528_1 | 15.3 |
| ICEPs304-1 | ICEPatATCC11528_2 | 14.3 |
| ICEPs304-1 | ICEPsa292-2 | 10.6 |
| ICEPs304-1 | ICEPsaNZ64_Cu | 7.6 |
| ICEPs309-1 | nh | 78.7 |
| ICEPs31R1 | ICEPsaNZ47_Cu | 23.5 |
| ICEPs31R1 | ICEPvirCDRTc14 | 21.4 |
| ICEPs31R1 | ICEPsaC6 | 16.1 |
| ICEPs31R1 | ICEPsaC27 | 11.2 |
| ICEPs31R1 | ICEPsaI12.29, ICEPsaI10 | 10 |
| ICEPs31R1 | ICEPsaC2 | 7.3 |
| ICEPs82L1 | ICEPco1_6 | 9.3 |
| ICEPs82L1 | ICEPs91L8 | 9 |
| ICEPs82L1 | ICEPsaNZ13, ICEPsBRIP34881, ICEPar4457 | 8.1 |
| ICEPs82L1 | ICEPs11168 | 7.4 |
| ICEPs82L1 | ICEPsy3023 | 6.7 |
| ICEPs82L1 | ICEPsBS3827 | 6 |
| ICEPs82L1 | ICEPsBS0292 | 5.3 |
| ICEPs91L8 | ICEPsBS3829 | 34 |
| ICEPs91L8 | ICEPsaNZ13, ICEPsaC18, ICEPsBRIP34881, ICEPs82L1, ICEPar4457, ICEPaf4394 | 11.1 |
| ICEPs91L8 | ICEPsaNZ13, ICEPsaC18, ICEPar4457 | 9.5 |
| ICEPs91L8 | ICEPs82L1 | 9.1 |
| ICEPs91L8 | ICEPsaNZ13, ICEPsaC18, ICEPsBRIP34881, ICEPar4457 | 8 |
| ICEPs91L8 | ICEPsaC3 | 6.7 |
| ICEPsa292-2 | ICEPatATCC11528_2 | 19 |
| ICEPsa292-2 | nh | 17.3 |
| ICEPsa292-2 | PatATCC11528_1 | 10.7 |
| ICEPsa292-2 | ICEPs304-1 | 10.3 |
| ICEPsa292-2 | ICEPs254-4 | 8.3 |
| ICEPsa292-2 | ICEPsaMAFF212063 | 5 |
| ICEPsaC11 | ICEPvirCDRTc14 | 16.2 |
| ICEPsaC11 | ICEPsaNZ13, ICEPsBRIP34881, ICEPar4457 | 11.7 |
| ICEPsaC11 | ICEPsaI12.29, ICEPsaI10 | 7 |
| ICEPsaC15 | ICEPsaNZ45_Cu, ICEPs164-1 | 28.9 |
| ICEPsaC15 | ICEPsaC3 | 24.2 |
| ICEPsaC15 | ICEPsyB728a, ICEPs164-1, ICEPpaLMG2367 | 12.3 |
| ICEPsaC15 | ICEPsPengzhou8 | 9.2 |
| ICEPsaC15 | ICEPs164-1 | 8.1 |
| ICEPsaC18 | ICEPsaNZ13, ICEPar4457 | 46.5 |
| ICEPsaC18 | ICEPsySM | 11.8 |
| ICEPsaC18 | nh | 7.9 |
| ICEPsaC2 | ICEPvirCDRTc14 | 16.9 |
| ICEPsaC2 | ICEPsaC6 | 16.3 |

| | | |
|---|---|---|
| ICEPsaC2 | ICEPsaI12.29, ICEPsaI10 | 15.1 |
| ICEPsaC2 | ICEPsaNZ47_Cu | 13.9 |
| ICEPsaC2 | ICEPsaC27 | 12.1 |
| ICEPsaC2 | ICEPs31R1 | 7.5 |
| ICEPsaC27 | ICEPvirCDRTc14 | 17.5 |
| ICEPsaC27 | ICEPsaI12.29, ICEPsaI10 | 17.5 |
| ICEPsaC27 | ICEPsaC2 | 14.4 |
| ICEPsaC27 | ICEPs31R1 | 13.3 |
| ICEPsaC27 | ICEPsaNZ47_Cu | 11.7 |
| ICEPsaC27 | ICEPsaC6 | 10.4 |
| ICEPsaC3 | ICEPsaC15 | 20.6 |
| ICEPsaC3 | ICEPsPengzhou8 | 10.4 |
| ICEPsaC3 | ICEPsBS3829 | 8.3 |
| ICEPsaC3 | ICEPsyB728a, ICEPsaNZ45_Cu, ICEPsaC15, ICEPs164-1, ICEPpaLMG2367 | 6.4 |
| ICEPsaC6 | ICEPsaNZ47_Cu | 35.2 |
| ICEPsaC6 | ICEPs31R1 | 17.8 |
| ICEPsaC6 | ICEPsaC2 | 14.9 |
| ICEPsaC6 | ICEPsaI12.29, ICEPsaI10 | 14.1 |
| ICEPsaC6 | ICEPvirCDRTc14 | 7.4 |
| ICEPsaC6 | ICEPsaC27 | 6.4 |
| ICEPsaC66 | ICEPsaNZ64_Cu | 33 |
| ICEPsaC66 | ICEPs2L4 | 21.5 |
| ICEPsaC66 | ICEPatATCC11528_2 | 6.9 |
| ICEPsaI10 | ICEPsaI12.29 | 100 |
| ICEPsaI12.29 | ICEPsaI10 | 100 |
| ICEPsaMAFF212063 | ICEPfm207-1 | 58.6 |
| ICEPsaMAFF212063 | ICEPstB13-200 | 19.8 |
| ICEPsaMAFF212063 | nh | 7.7 |
| ICEPsaNZ13 | ICEPar4457 | 97 |
| ICEPsaNZ45_Cu | ICEPs164-1 | 37.2 |
| ICEPsaNZ45_Cu | ICEPpaLMG2367 | 35.7 |
| ICEPsaNZ47_Cu | ICEPsaC6 | 33.6 |
| ICEPsaNZ47_Cu | ICEPsaI12.29, ICEPsaI10 | 16.6 |
| ICEPsaNZ47_Cu | ICEPs31R1 | 16.6 |
| ICEPsaNZ47_Cu | ICEPsaC2 | 11.2 |
| ICEPsaNZ47_Cu | ICEPsaC27 | 10.3 |
| ICEPsaNZ64_Cu | ICEPsaC66 | 34.5 |
| ICEPsaNZ64_Cu | ICEPs2L4 | 24.3 |
| ICEPsaNZ64_Cu | ICEPsaNZ13, ICEPar4457 | 6.7 |
| ICEPsaNZ64_Cu | ICEPatATCC11528_2 | 6.3 |
| ICEPsaP155 | ICEPsaNZ45_Cu, ICEPsaC15, ICEPs164-1 | 12.8 |
| ICEPsaP155 | ICEPsyB728a, ICEPsaNZ45_Cu, ICEPsaC15, ICEPs164-1, ICEPpaLMG2367 | 8.2 |
| ICEPsaP155 | ICEPsBS3829 | 7.3 |
| ICEPsaP155 | ICEPsBS3829, ICEPs91L8 | 6.3 |
| ICEPsaP155 | ICEPsy41A01 | 5.7 |
| ICEPsatLMG2095 | ICEPaf4394 | 53.9 |
| ICEPsatLMG2095 | ICEPco1_6 | 34.1 |
| ICEPsatLMG2095 | ICEPsPengzhou8, ICEPaf4394 | 10 |

| | | |
|---|---|---|
| ICEPsBRIP34881 | ICEPsaNZ13, ICEPar4457 | 53.7 |
| ICEPsBRIP34881 | ICEPsBS0292 | 36.2 |
| ICEPsBRIP39023 | ICEPsKN2.a.3 | 16.3 |
| ICEPsBRIP39023 | ICEPsLMC.P91 | 12.5 |
| ICEPsBRIP39023 | ICEPatATCC11528_2 | 8.6 |
| ICEPsBRIP39023 | ICEPph1302A | 8.2 |
| ICEPsBRIP39023 | ICEPsaC66 | 5 |
| ICEPsBS0292 | ICEPsBRIP34881 | 34.6 |
| ICEPsBS0292 | ICEPsyNZIPFR-PS7 | 8.9 |
| ICEPsBS0292 | ICEPsatLMG2095, ICEPaf4394 | 6.8 |
| ICEPsBS3827 | ICEPs11168 | 17.2 |
| ICEPsBS3827 | ICEPar4457 | 8.7 |
| ICEPsBS3827 | ICEPsaNZ13, ICEPsaC18, ICEPar4457 | 7 |
| ICEPsBS3827 | ICEPsaNZ13, ICEPsaC18, ICEPs91L8, ICEPar4457 | 6.5 |
| ICEPsBS3827 | ICEPs82L1 | 5.8 |
| ICEPsBS3827 | ICEPsBS3829 | 5.3 |
| ICEPsBS3829 | ICEPs91L8 | 39.7 |
| ICEPsBS3829 | ICEPsaC3 | 11.9 |
| ICEPsBS3829 | ICEPsBS3827 | 6.5 |
| ICEPsBS3829 | ICEPsyB728a, ICEPsaNZ45_Cu, ICEPsaC15, ICEPs164-1, ICEPpaLMG2367 | 5.2 |
| ICEPsKN2.a.3 | ICEPsLMC.P91 | 20.8 |
| ICEPsKN2.a.3 | ICEPsBRIP39023 | 13.2 |
| ICEPsKN2.a.3 | ICEPsaNZ13, ICEPsaC18, ICEPsBRIP34881, ICEPs91L8, ICEPs82L1, ICEPar4457, ICEPaf4394 | 9.2 |
| ICEPsKN2.a.3 | ICEPph1302A | 5.4 |
| ICEPsKN2.a.3 | ICEPsy41A01, ICEPsaNZ13, ICEPsLMC.P91, ICEPsBRIP34881, ICEPs91L8, ICEPs82L1, ICEPs2L4, ICEPs11168, ICEPar4457 | 5 |
| ICEPsLMC.P91 | ICEPsyHS191 | 27.9 |
| ICEPsLMC.P91 | ICEPsKN2.a.3 | 18.9 |
| ICEPsLMC.P91 | ICEPsBRIP39023 | 10.4 |
| ICEPsLMC.P91 | ICEPsy41A01, ICEPsaNZ13, ICEPsBRIP34881, ICEPs91L8, ICEPs82L1, ICEPs2L4, ICEPs11168, ICEPar4457 | 7 |
| ICEPsLMC.P91 | ICEPsaNZ13, ICEPsaC18, ICEPsBRIP34881, ICEPs91L8, ICEPs82L1, ICEPs2L4, ICEPar4457, ICEPaf4394 | 5.6 |
| ICEPsPengzhou8 | ICEPaf4394 | 53 |
| ICEPsPengzhou8 | ICEPsaC3 | 17.2 |
| ICEPsPengzhou8 | ICEPsaC15 | 6.7 |
| ICEPsPengzhou8 | ICEPsaC11 | 6.1 |
| ICEPstB13-200 | ICEPfm207-1 | 51.4 |
| ICEPstB13-200 | ICEPsaMAFF212063 | 33.7 |
| ICEPsy2340 | ICEPsy3023 | 8.3 |
| ICEPsy2340 | ICEPph1302A | 6.6 |
| ICEPsy2340 | ICEPsyB728a, ICEPsaNZ45_Cu, ICEPsaC15, ICEPs164-1, ICEPpaLMG2367 | 6 |
| ICEPsy3023 | ICEPsy2340 | 10.9 |
| ICEPsy3023 | ICEPsyNZIPFR-PS7 | 6.3 |
| ICEPsy41A01 | ICEPco19117 | 30.1 |
| ICEPsy41A01 | ICEPsaNZ13, ICEPsBRIP34881, ICEPs2L4, ICEPar4457 | 12.9 |

| | | |
|---|---|---|
| ICEPsy41A01 | ICEPs11168 | 10.9 |
| ICEPsy41A01 | ICEPsBS3827 | 10.8 |
| ICEPsy41A01 | ICEPsaNZ13, ICEPsBRIP34881, ICEPar4457 | 5.5 |
| ICEPsyB728a | ICEPs164-1 | 27.3 |
| ICEPsyB728a | ICEPsaNZ45_Cu, ICEPsaC15, ICEPs164-1 | 17.2 |
| ICEPsyB728a | ICEPsyHS191 | 10.8 |
| ICEPsyB728a | ICEPph1302A | 5.1 |
| ICEPsyHS191 | ICEPsLMC.P91 | 26.9 |
| ICEPsyHS191 | ICEPph1302A | 12.8 |
| ICEPsyHS191 | ICEPsyB728a | 12.2 |
| ICEPsyHS191 | ICEPsatLMG2095, ICEPsBS0292, ICEPaf4394 | 5.2 |
| ICEPsyHS191 | ICEPsy2340 | 5.1 |
| ICEPsyNZIPFR-PS7 | ICEPsBS0292 | 12.6 |
| ICEPsyNZIPFR-PS7 | ICEPsaNZ13, ICEPar4457 | 11.3 |
| ICEPsyNZIPFR-PS7 | ICEPsy3023 | 7.2 |
| ICEPsyNZIPFR-PS7 | ICEPsyB728a | 6.8 |
| ICEPsySM | ICEPsaC18 | 9.4 |
| ICEPsySM | ICEPsaC15 | 6.4 |
| ICEPvirCDRTc14 | ICEPsaC27 | 18.4 |
| ICEPvirCDRTc14 | ICEPs31R1 | 17.3 |
| ICEPvirCDRTc14 | ICEPsaC11 | 15.8 |
| ICEPvirCDRTc14 | ICEPsaC2 | 14.9 |
| ICEPvirCDRTc14 | ICEPsaI12.29, ICEPsaI10 | 8 |
| ICEPvirCDRTc14 | ICEPsaC6 | 7.8 |
| ICEPvirCDRTc14 | ICEPsaNZ47_Cu | 5.6 |
| PatATCC11528_1 | ICEPs248-6 | 25.1 |
| PatATCC11528_1 | ICEPatATCC11528_2 | 22.7 |
| PatATCC11528_1 | ICEPs304-1 | 13.2 |
| PatATCC11528_1 | ICEPsa292-2 | 11.3 |
| PatATCC11528_1 | ICEPs254-4 | 8.4 |

**Table S5. Genes in Tn*6212* .**

| *Psa* NZ13 accession number | gene | predicted function | ΔTn*6212* | Tn*6212* Δ1 | Tn*6212* Δ2 | Tn*6212* Δ3 |
|---|---|---|---|---|---|---|
| IYO_024500 | *MCP* | a methyl-accepting chemotaxis protein predicted to be involved in taxis toward malate | deleted | deleted | | |
| | hp | protein of unknown function (DUF1264) | deleted | deleted | | |
| IYO_024505 | *mer* | transcriptional regulator | deleted | deleted | | |
| IYO_024510 | transporter | transporter related to the human bile acid:sodium symporters or arsenite efflux pump ACR3 | deleted | deleted | | |
| IYO_024515 | hp | unknown | deleted | deleted | | |
| IYO_024520 | cl channel | voltage-gated chloride channel | deleted | deleted | | |
| IYO_024525 | hp | unknown | deleted | | deleted | |
| IYO_024530 | hp | unknown | deleted | | deleted | |
| IYO_024535 | hp | unknown | deleted | | deleted | |
| IYO_024540 | hp | unknown | deleted | | deleted | |
| IYO_024545 | *eno* | enolase | deleted | | deleted | |
| IYO_024550 | *ppa* | inorganic phosphatase | deleted | | deleted | |
| IYO_024555 | hp | uracil-DNA glycosylase | deleted | | deleted | |
| IYO_024560 | *dctT* | di-carboxylic acid transporter | deleted | | | |
| IYO_024565 | *lysR* | transcriptional regulator | deleted | | | deleted |
| IYO_024570 | *cta* | catabolism-associated protein | deleted | | | deleted |
| IYO_024575 | *lpr* | lipoprotein | deleted | | | deleted |
| IYO_024580 | *xerC* | tyrosine recombinase | | | | deleted |

## Table S6. Plasmids and strains

| Plasmid Name | Characteristic | Reference |
|---|---|---|
| pGEM®-T | high copy number cloning vector: *AmpR* , pBR322 ori | Promega |
| pKR2013 | Helper plasmis for tri-parental mating. KmR, *incP4* , *tra* , *mob* . | Ditta et al., 1980 |
| pK18mobsacB | Mobilizable vector *sacB* , *lacZa* , KmR, mcs | Schafer et al., 1994 |
| pUC18-mini-Tn*7T* -LAC | Chromosome integration vector wirh inducible Ptac promoter; AmpR, Gr | Choi and Schweizer, 2006 |

| Strain Name | Characteristic | Reference |
|---|---|---|
| *E. coli* S17-1 Tn*5hah* Sgid1 | Thi-Pro-Hsd-*recA* -zzz::RP4-2 (*tet* ::Mu, *kan* ::Tn*7* [TpR, SmR]) | Zhang et al., 2015 |

**Table S7. Primers**

| Primer name | Primer sequence |
|---|---|
| Δ1_BamHI_For | AAGGATCCGAGTTTACGCCGGATCGTTG |
| Δ1_Cross_For | AGTTTAAGTTTAGTAGTACTGTGCGGGGGAAGAGAAATGAT |
| Δ1_Cross_Rev | CAGTACTACTAAACTTAAACTGAGGCCTGGTCTTGAAGCTA |
| Δ1_Ext_For | TGCAAGTCGCTGGTTTTCAA |
| Δ1_Ext_Rev | ATTTTGATGAGCCGACGTCG |
| Δ1_HindIII_Rev | ATAAGCTTACTTTTCACTGGGCATACGC |
| Δ2_BamHI_For | AAGGATCCCCGATCAACTGACGCATGTC |
| Δ2_Cross_For | AGTTTAAGTTTAGTAGTACTGACTCACCCGAATGTTAGGCA |
| Δ2_Cross_Rev | CAGTACTACTAAACTTAAACTTGTCAGGCGATCTTGGTGAT |
| Δ2_Ext_For | CTGATCGGAACCGTGGTTTC |
| Δ2_Ext_Rev | CAGTACTACTAAACTTAAACTAGCGCGAAAATGAGTGAGAC |
| Δ2_HindIII_Rev | ATAAGCTTATCGTGCTGTTTTGCCTGTT |
| Δ3_BamHI_For | AAGGATCCGTTGTTACGGCGTACACTCC |
| Δ3_Cross_For | AGTTTAAGTTTAGTAGTACTGCGCTGCCCTTTAAGTGTTGT |
| Δ3_Cross_Rev | CAGTACTACTAAACTTAAACTAGCGCGAAAATGAGTGAGAC |
| Δ3_Ext_For | AGTTTAAGTTTAGTAGTACTGACTCACCCGAATGTTAGGCA |
| Δ3_Ext_Rev | AAGCTTTGCATCCTCCAACG |
| Δ3_PstI_Rev | AACTGCAGTGTCAGTTCCGTACCTGGAC |
| Δcatass_BamHI_Rev | AAGGATCCGCCCGCATATTTACGATGCC |
| Δcatass_Cross_For | AGTTTAAGTTTAGTAGTACTGACGACTGATTGAACAGCGCAG |
| Δcatass_Cross_Rev | TCAGTACTACTAAACTTAAACTCAACTCGTTCATCGCTGCAG |
| Δcatass_EcoRI_For | AAGAATTCAAGCTAAGCCTACGCCAGC |
| ΔdctT_BamHI_Rev | TTGGATCCAGCTACTCCTGGCTCACGAA |
| ΔdctT_Cross_For | GGTTTAAGTTTAGTAAGCTTGGGCCCATTATAGCTGCGAGTC |
| ΔdctT_Cross_Rev | CCAAGCTTACTAAACTTAAACCCCTGAGCCTTTGGCTACTTG |
| ΔdctT_EcoRI_For | TTGAATTCGATCTTCGTTGATCGCTTCC |
| ΔdctT_Ext_For | AATCAAATCGGAACGCTGAC |
| ΔdctT_Ext_Rev | TTGTGAATATCGCCGTCAAA |
| Δeno_Cross_For | GGTTTAAGTTTAGTAAGCTTGCCGGTAAACCTGTTGGACTG |
| Δeno_Cross_Rev | CAAGCTTACTAAACTTAAACTCCTGGATTTGCGTTTTCATT |
| Δeno_EcoRI_For | AAGAATTCGCGGTACAGAGGGGCTAGAT |
| Δeno_Ext_For | ACGGCTTTCAACTGACGTTT |
| Δeno_Ext_Rev | TGACGCAGTGTGGACAAAAT |
| Δeno_XmaI_Rev | AACCCGGGCAGGGAAAAGCGTCAGTAGC |
| Δlipo_BamHI_For | AAGGATCCACCTCTCCGACAGCAGTTTG |
| Δlipo_Cross_For | AGTTTAAGTTTAGTAGTACTCTTTGTCAGTGGCGCAAACC |
| Δlipo_Cross_Rev | AGTACTACTAAACTTAAACTAAGACCCACCTGCCTCTGAA |
| Δlipo_HindIII_Rev | ATAAGCTTCGGGCGTATTCATCAGTACC |
| ΔlysR_BamHI_For | AAGGATCCGTTGTTACGGCGTACACTCC |
| ΔlysR_Cross_For | AGTTTAAGTTTAGTAGTACTGAATTCGTGAGCCAGGAGTAG |
| ΔlysR_Cross_Rev | CAGTACTACTAAACTTAAACTGGCGTAGGCTTAGCTTCAT |
| ΔlysR_HindIII_Rev | ATAAGCTTTGGTGAAGACAATGGCCTGG |
| ΔTn6212_Cross_For | GTTTAAGTTTAGTGATATCGGACGTATACCGGTAGCGACGA |
| ΔTn6212_Cross_Rev | CCGATATCACTAAACTTAAACTCAAACTGGCCACCTCTACC |
| ΔTn6212_EcoRI_Rev | TTTGAATTCTAACGGCCATAACCGACAAT |
| ΔTn6212_Ext_For | GGTGTCGGCATGTAGAAAGG |
| ΔTn6212_Ext_Rev | CCTTGTGCCAGAATCCATCT |
| ΔTn6212_XbaI_For | TTTTCTAGAATTTGATACGCCCAAAACCA |
