# Supplementary material for: Rapid dissemination of host metabolism–manipulating genes via integrative and conjugative elements
Source: Proc Natl Acad Sci U S A. 2024 Mar 8;121(11):e2309263121. doi: 10.1073/pnas.2309263121 (PMC10945833; doi:10.1073/pnas.2309263121)
Supplement: Supplementary file 2 — Appendix 02 (PDF) [file pnas.2309263121.sapp2.pdf]

Supplementary Figures for

Rapid dissemination of host metabolism-manipulating genes via integrative and conjugative elements

Elena Colombi, Frederic Bertels, Guilhem Doulcier, Ellen McConnell, Tatyana Pichugina, Kee Hoon Sohn, Christina Straub, Honour C. McCann* and Paul B. Rainey*

Email: honour.mccann@tuebingen.mpg.de, rainey@evolbio.mpg.de

Contents

Figure S1. Phylogeny of the strains harbouring the PsICEs……………………..………………………2

Figure S2. PsICEs are members of a broad family of ICEs circulating in *Pseudomonas*…………..…..3

Figure S3. The backbone genes of the PsICEs………………………………………………………......4

Figure S4. Neighbor-net network tree of backbone genes of PsICEs..…………………………….......5

Figure S5. Conservation of Tn*6212*.……………………………......……………………………..........6

Figure S6. Tn*6212* DctT secretion assays.……………………..………...………………………………7

Figure S7. Growth of *Psa* NZ13 and *Psa* NZ13 ΔTn*6212* on kiwifruit……………………………….....8

Figure S8. Tn*6212* mutant fitness on multiple carbon sources.……….……………………….............9

Figure S9. Expression correlation between *Psa* NZ13ΔTn*6212* and *Psa* NZ13 Δ*lysR*…….…………..10

Figure S10. Colony expansion rate on multiple carbon sources. …………………………….…...........11

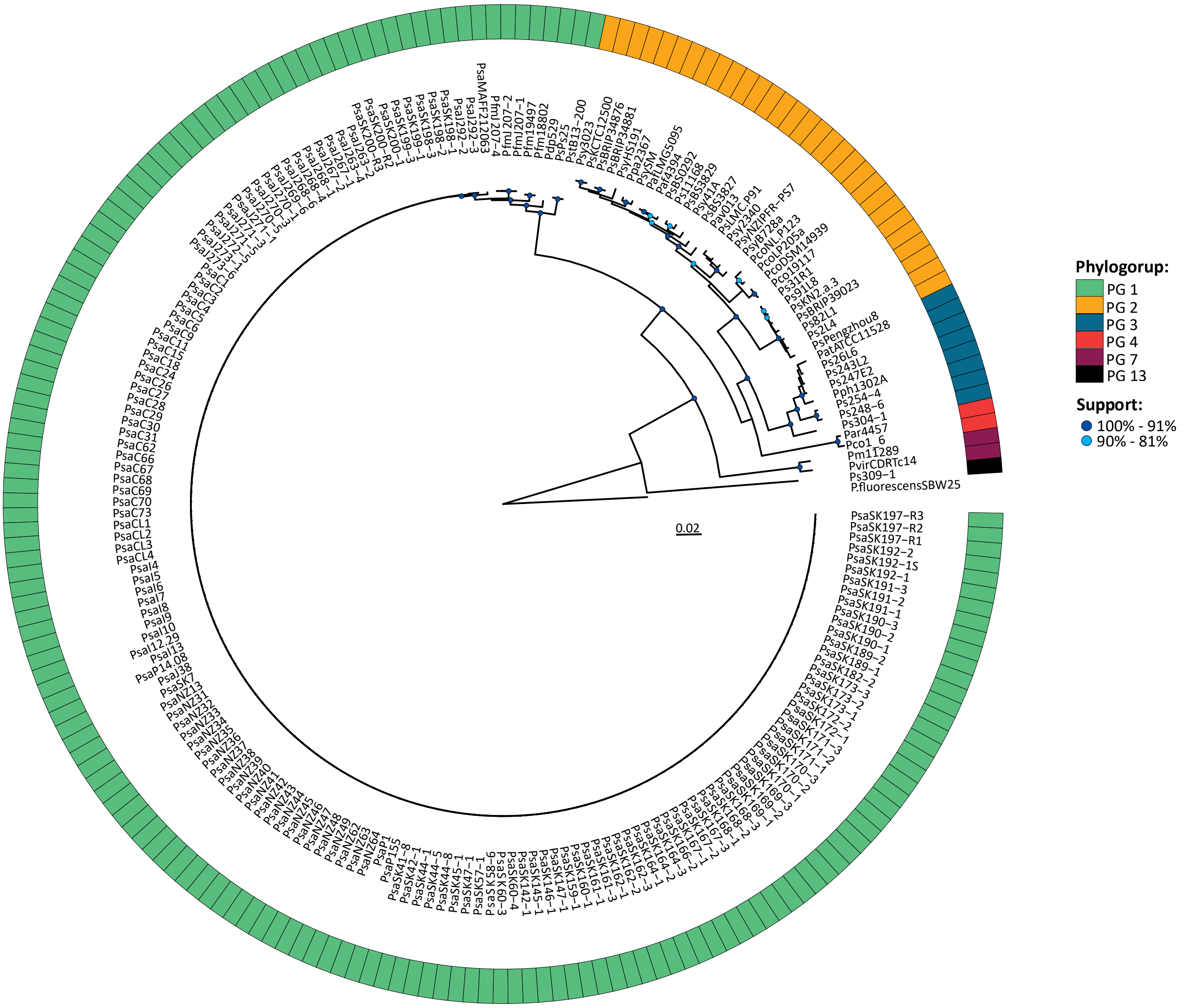

Fig. S1. Phylogeny of the strains harbouring the PsICEs. PhyML tree based on the concatenation of *gapA*, *gltA*, *gyrB* and *rpoD*. *P. fluorescens* SBW25 was used as outgroup. The ring displays the phylogroup of the *P. syringae* species complex strain. Support indicates bootstrap support values. Scale bar indicates substitution per site.

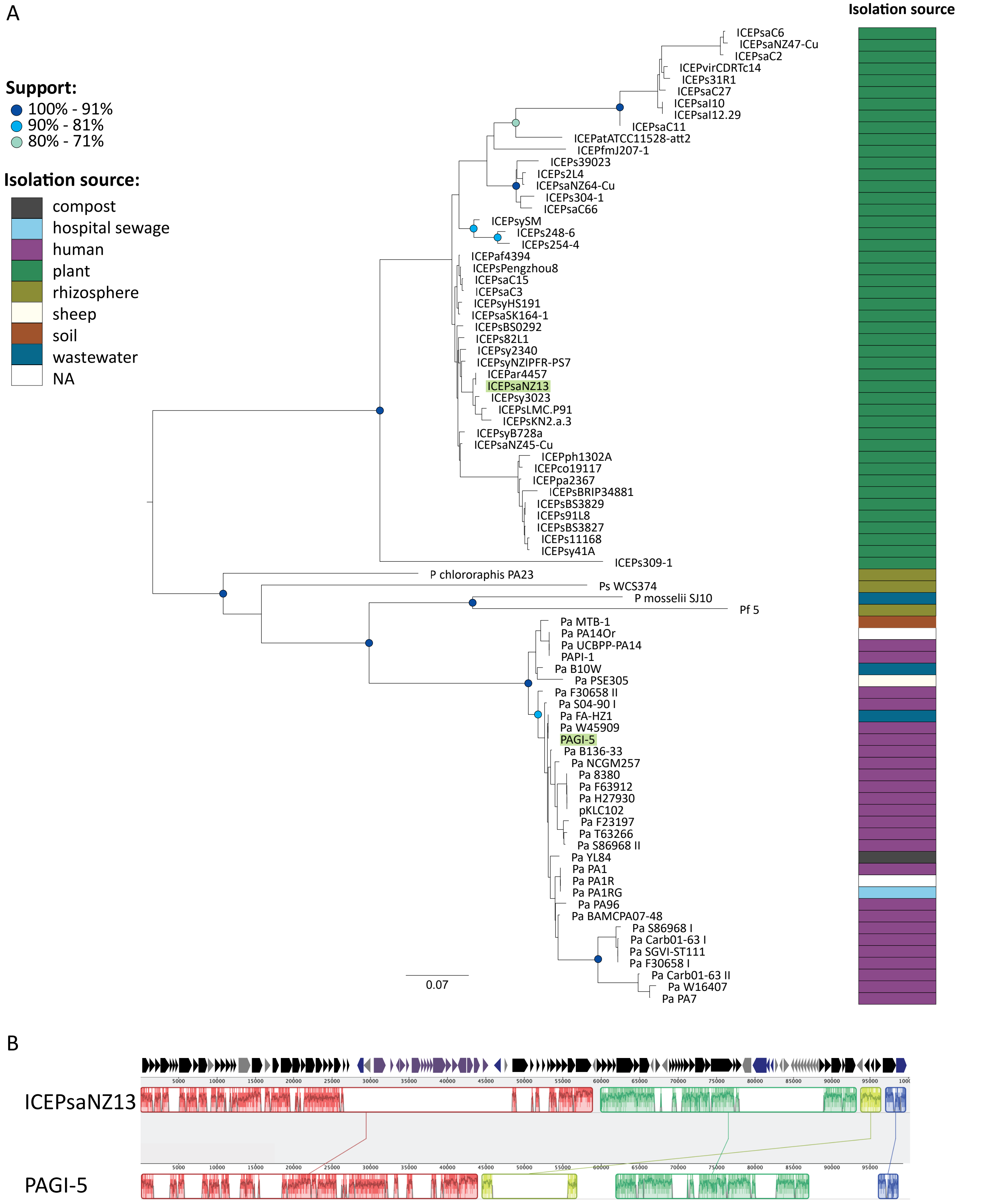

Fig. S2. PsICEs are members of a broad family of ICEs circulating in the genus Pseudomonas. A) *parA*, *topIII* and *xerC* were aligned with MAFFT and concatenated. After gap removal, the maximum-likelihood tree was built with PhyML and the tree was rooted at midpoint. Support indicates bootstrap support values. ICEs in other species are identified according to accepted names (e.g. 'PAGI-5', 'pKLC102') or using the species/pathovar prefix and strain ID suffix (e.g. 'PaPA1'). Scale bar indicates substitution per site. B) A progressiveMauve alignment of entire ICEPsaNZ13 (representative of *P. syringae* ICEs) and entire PAGI-5 ICE (representative of the *P. aeruginosa* pKLC102/PAGI-2 family of ICEs). Colored regions in the progressiveMauve alignment indicate LCBs (locally colinear blocks) across the two ICEs. Colourless stretches indicate ICE unique regions. The genes in the map of ICEPsaNZ13 are colored as in Figure 2.

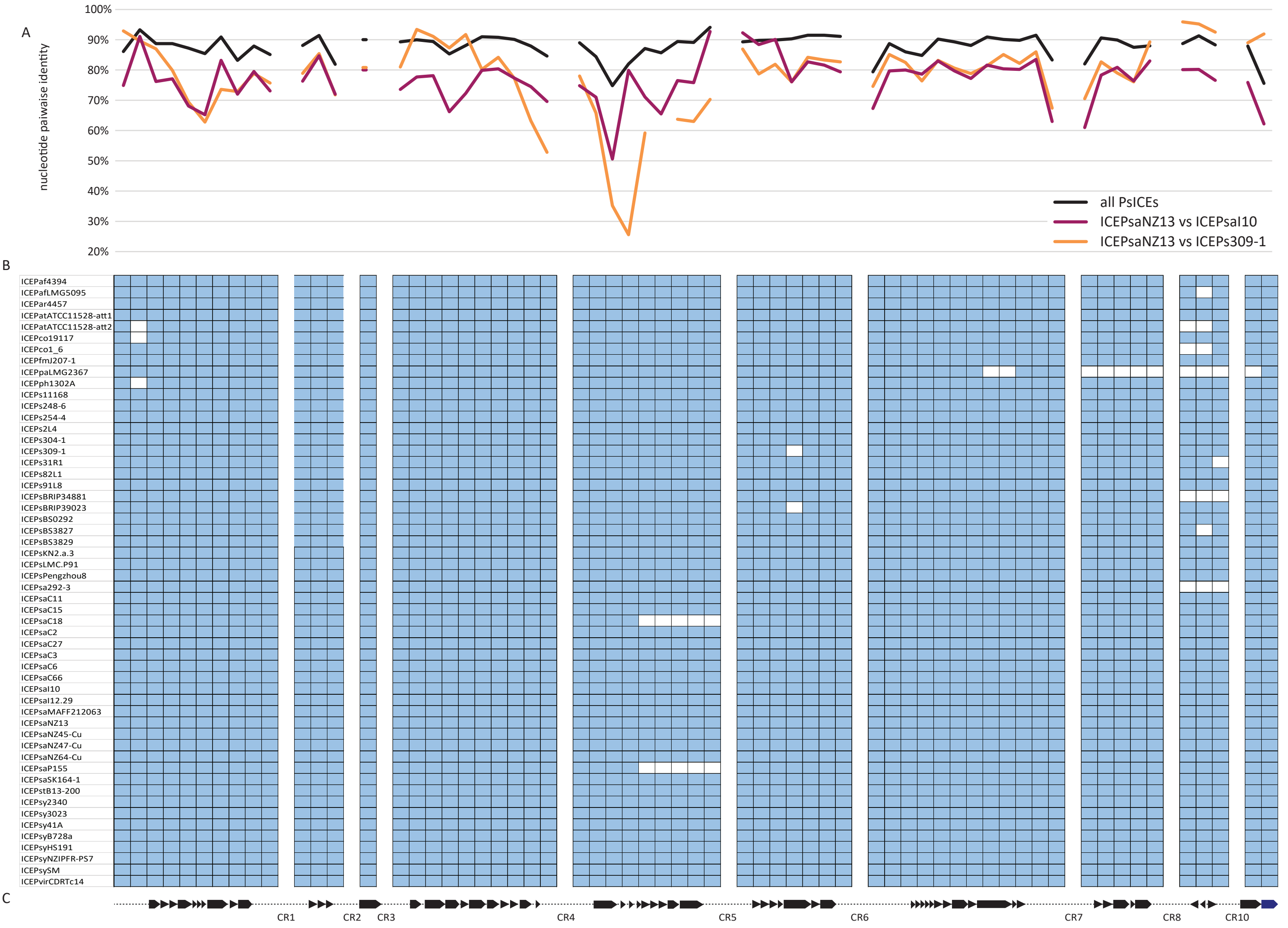

Fig. S3. The backbone genes of the PsICEs. A) Nucleotide pairwise identity of each backbone gene. B) Graphical representation of the backbone genes. C) Presence and absence of backbone genes in the non-redundant PsICE. In light blue gene presence, in white gene absence. CR stands for cargo region.

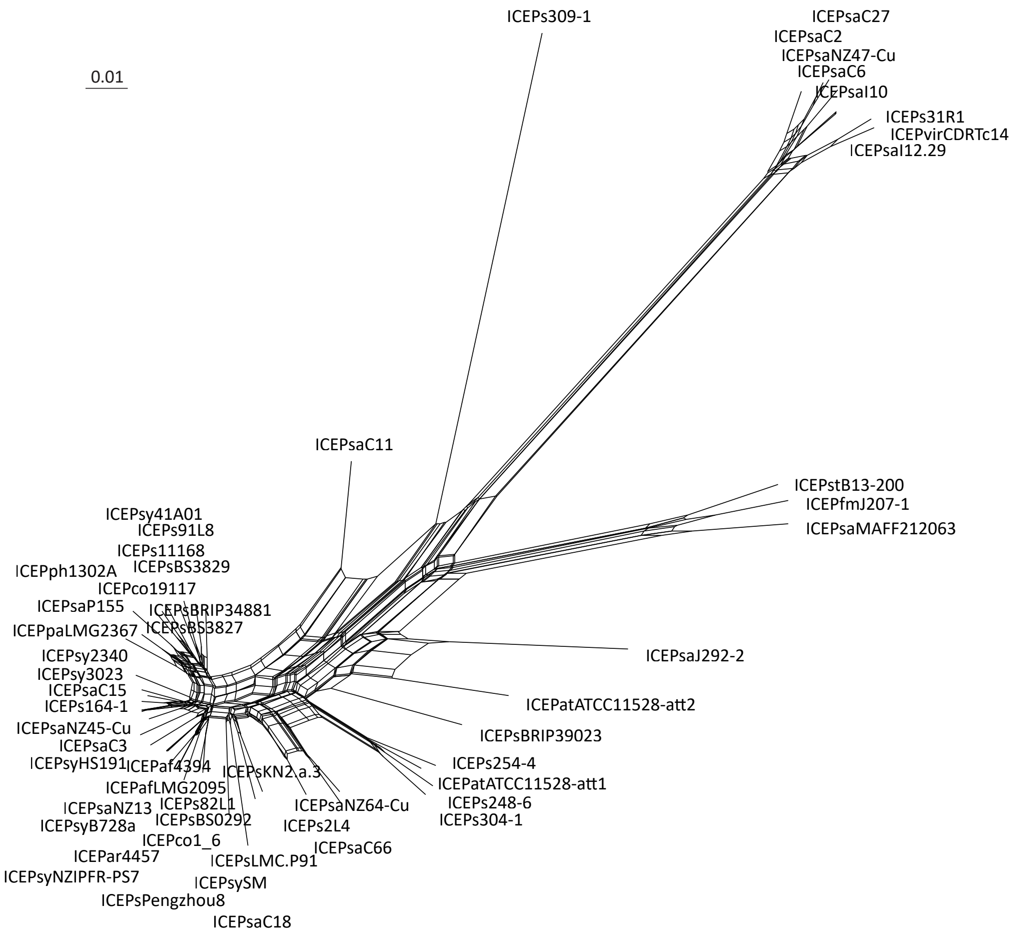

Fig. S4. Neighbor-net network tree of backbone genes of PsICEs. Neighbor-Net generated in Splitstree using a concatenated alignment of 58 backbone genes conserved in all 53 non-redundant ICEs. Scale bar indicates substitution per site.

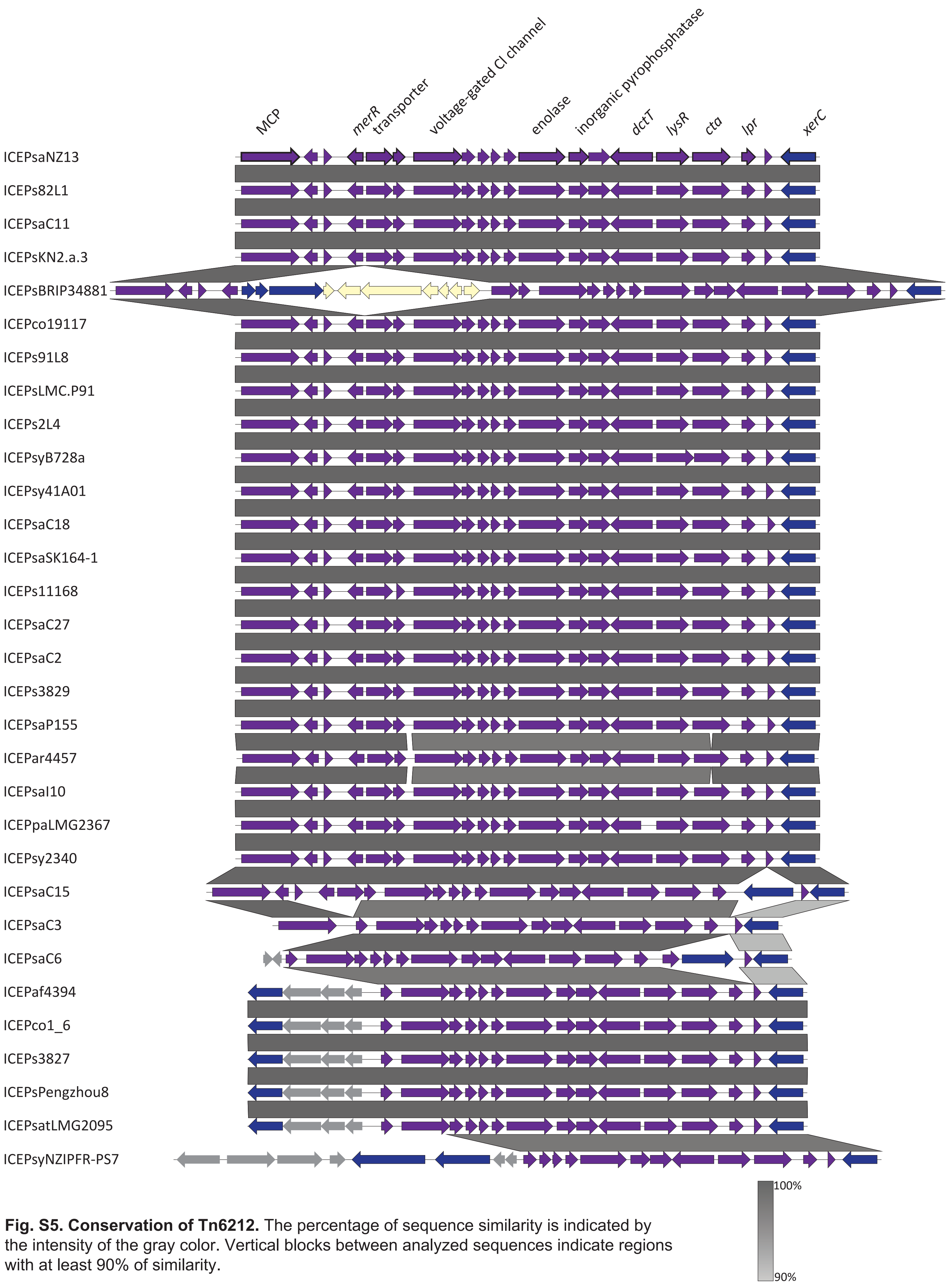

Fig. S5. Conservation of Tn6212. The percentage of sequence similarity is indicated by the intensity of the gray color. Vertical blocks between analyzed sequences indicate regions with at least 90% of similarity.

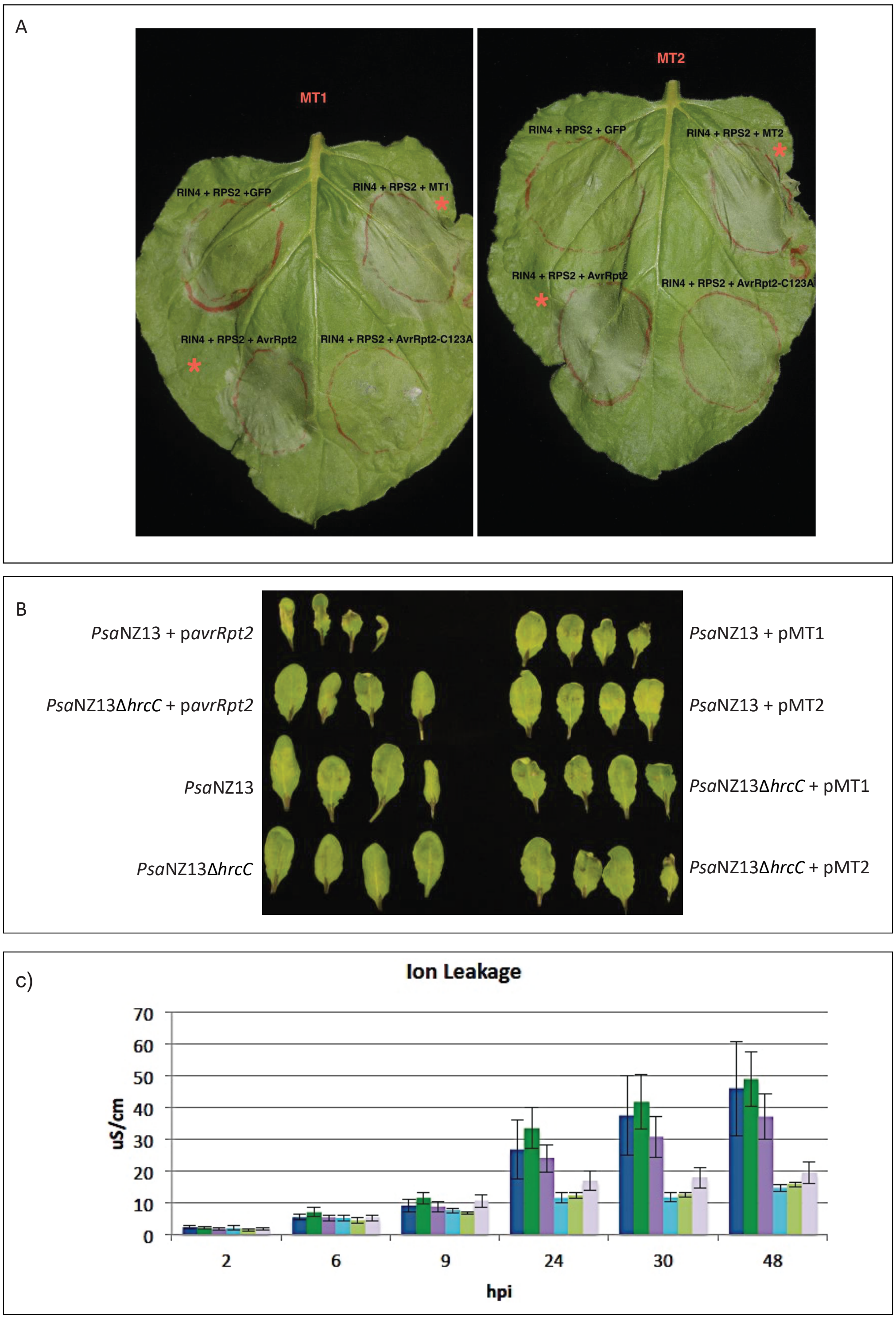

Fig. S6. Tn6212 DctT secretion assays. A) Agroinfiltration in *Nicotiana bethamiana* leaves using the indicated expression constructs. Pictures were taken at 24 hpi, an asterisk indicates hypersensitive response (HR) observed. B) Secretion assay in *Arabidopsis thaliana*. Leaves of *A. thaliana* Col-0 were pressure infiltrated with the indicated strain and HR development recorded at 48 hpi. The experiment was repeated two times inoculating two leaves of three plants per strain used. C) Ion leakage in *A. thaliana*. Conductivity (µS/cm) of solution containing leaf discs inoculated with *Psa* N13 (blue bars), *Psa* NZ13 + pMT-1 (dark green bars), *Psa* NZ13 + pMT-2 (purple bars), *Psa* NZ13 Δ*hrcC* (azure bars), *Psa* NZ13 Δ*hrcC* + pMT-1 (light green bars) and *Psa* NZ13 Δ*hrcC* + pMT-2 (lilac bars). Data are means and standard deviation of four replicates.

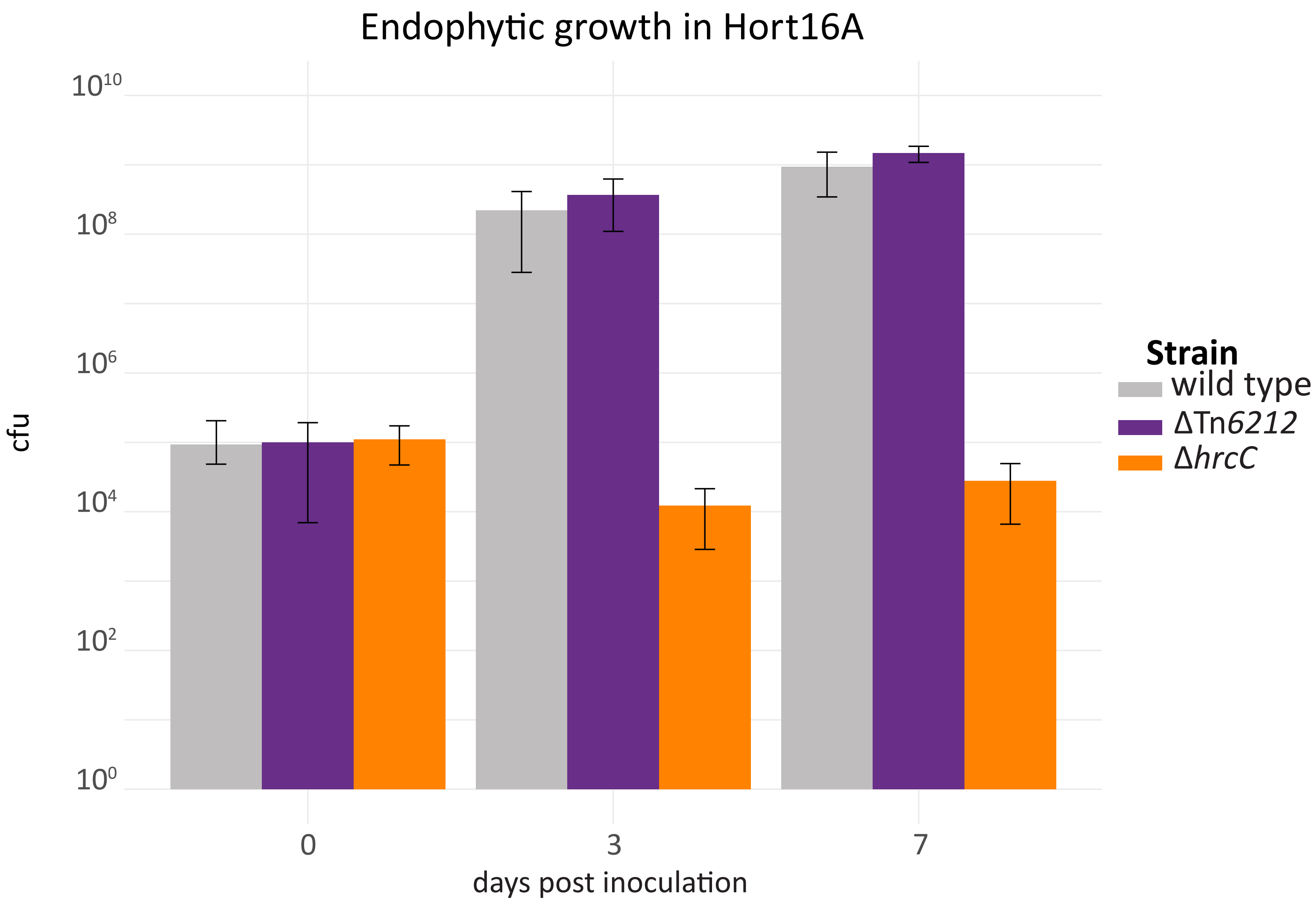

Fig S7. Growth of *Psa* NZ13 and *Psa* NZ13 ΔTn*6212* on kiwifruit. Growth of *Psa* NZ13 (grey bars), *Psa* NZ13 ΔTn*6212* (purple bars) and *Psa* NZ13 Δ*hrcC* (orange bars) was assessed endophytically on leaves of the kiwifruit cultivar Hort16A. Data are means and standard deviation of five replicates. Two tailed t-test revealed no statistical difference ($P>0.05$) between *Psa* NZ13 and the Tn*6212* mutants.

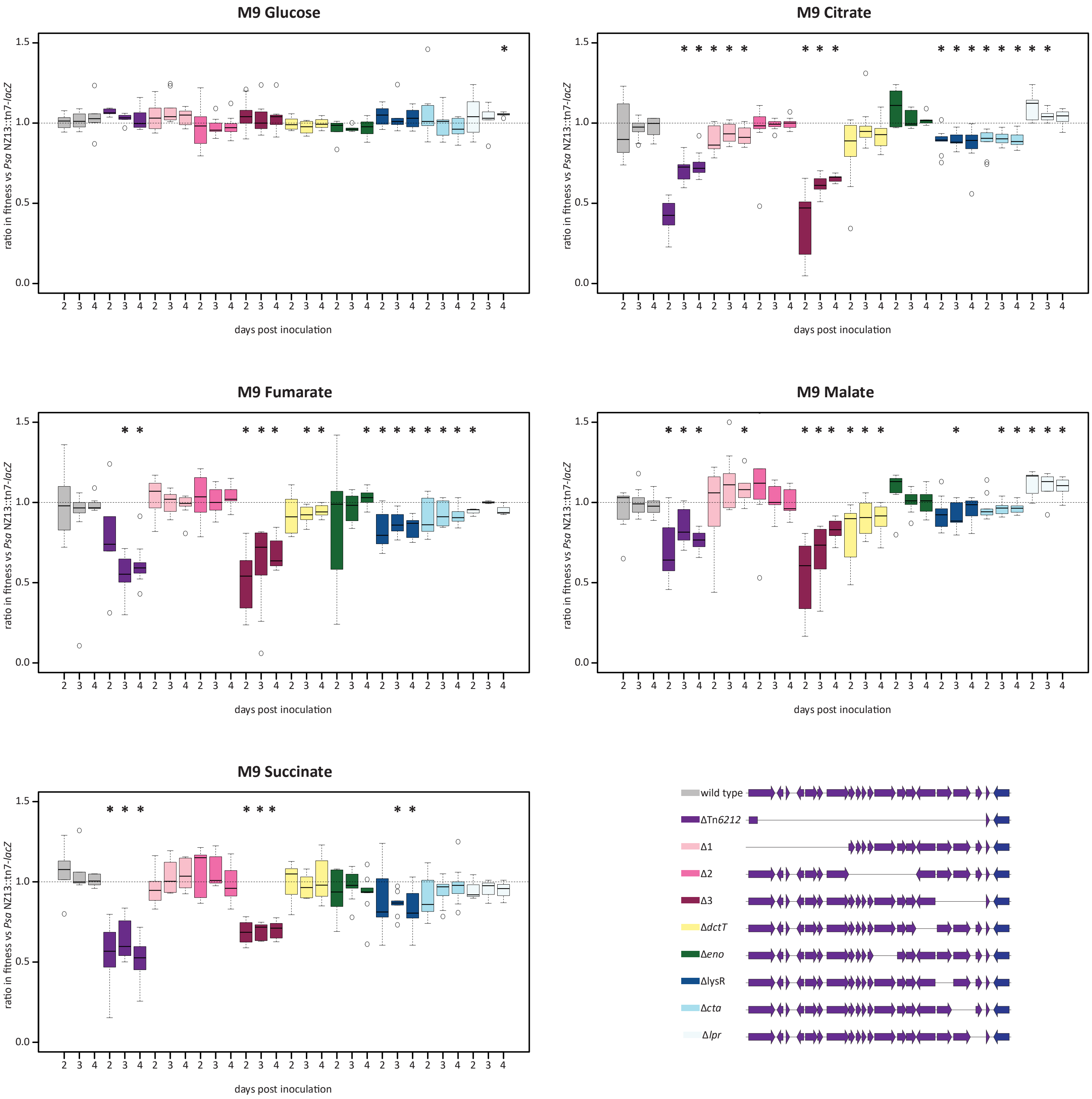

Fig. S8. Tn6212 mutant fitness on multiple carbon sources. Box plots of the fitness of the *Psa* NZ13 and the mutants on Tn*6212* versus *Psa* NZ::tn7-lacZ. Competition assays were established using equal starting densities (1:1) in M9 minimal medium supplemented with different carbon sources, measuring wildtype to mutant growth at 2, 3 and 4 days post inoculation. Values smaller than 1 indicate the competitor exhibits lower fitness relative to the wildtype strain. The experiment was performed with three replicates and repeated three times. From left to right, *Psa* NZ13 wild type, *Psa* NZ13ΔTn*6212, Psa* NZ13 Tn*6212*Δ1, *Psa* NZ13 Tn*6212*Δ2, , *Psa* NZ13 Tn*6212*Δ3, Psa *NZ13* Δ*dctT*, *Psa* NZ13 Δ*eno*, *Psa* NZ13 Δ*lysR*, *Psa* NZ13 Δ*cta*, *Psa* NZ13 Δ*lpr*. An asterisk indicates the fitness difference is statistically significant (one-sided one sample t-test $p < 0.05$).

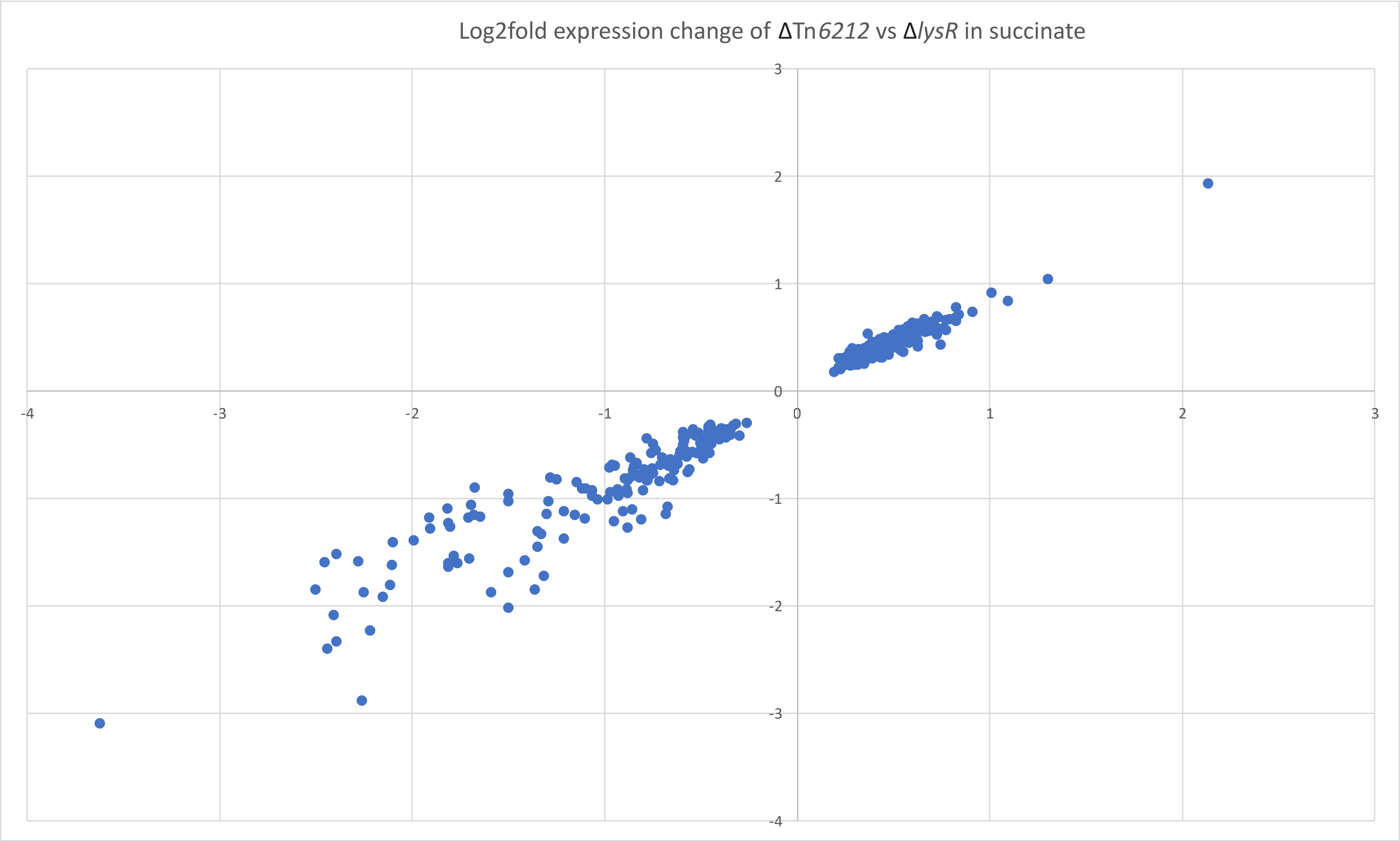

Fig. S9. Correlation between genes exhibiting significant expression fold change in *Psa* NZ13ΔTn*6212* and *Psa* NZ13 Δ*lysR*. Log2fold expression change of genes exhibiting significant differences in both ΔTn*6212* (x-axis) and Δ*lysR* (y-axis) strains during growth on succinate.

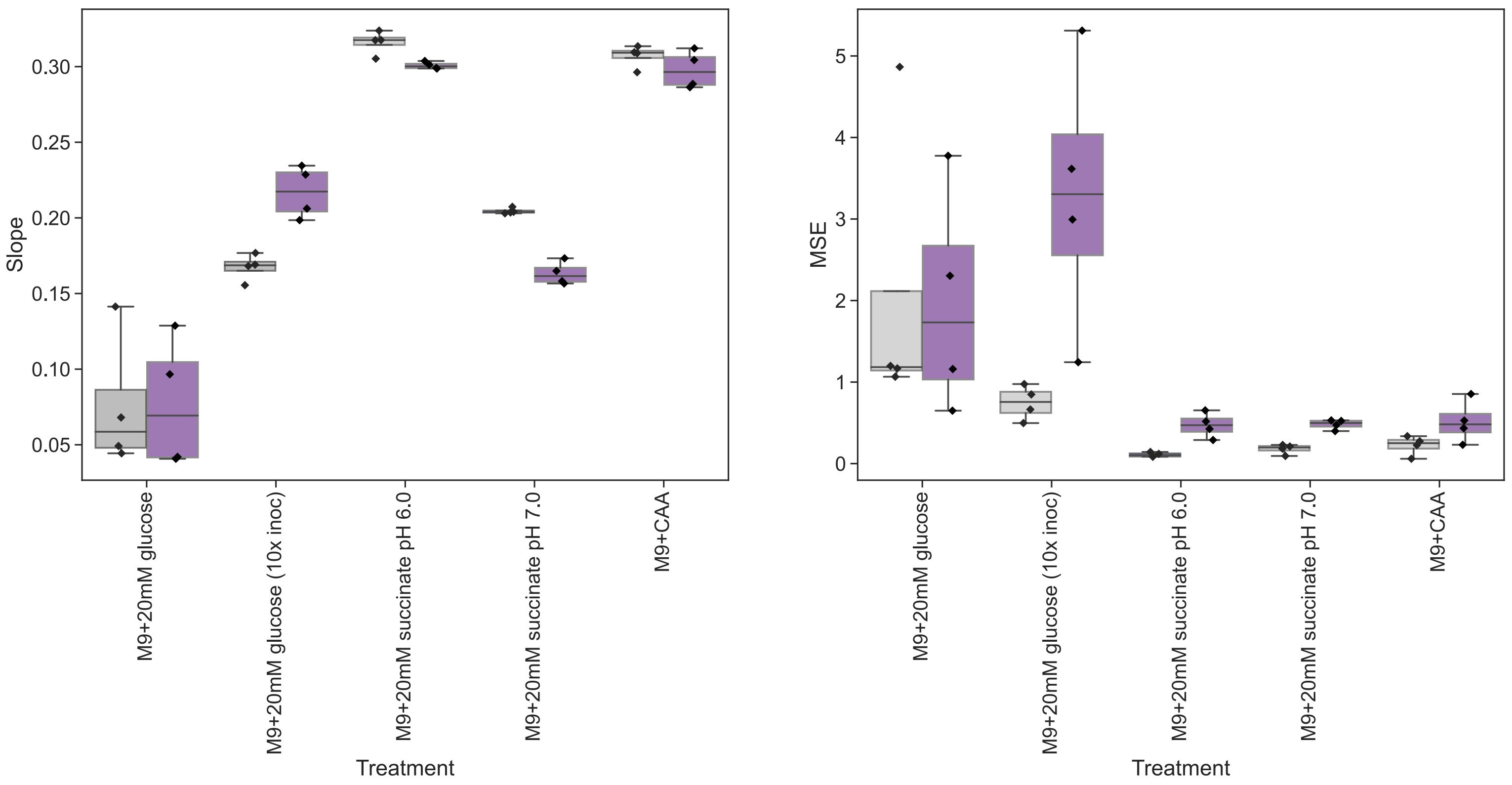

Fig. S10. Colony expansion rate on multiple carbon sources. The dynamics of colony growth was fitted with a line (Swarm = Slope * t + Intercept). Here, we show the statistics of Slope parameter (left panel), which is the expansion rate of colonies. The mean square error (MSE) is the average squared difference between the predicted fit and the actual data value, which is an estimator of the quality of fit. On panels, each colony is represented by a dot. They are grouped by the treatment (x-axis) and genotype (bar color: grey – *Psa* NZ13, purple – ΔTn*6212*). The boxplots display datasets based on the five-number summary: the minimum, the maximum (shown as whiskers), the sample median (central line), and the first and third quartiles. The parameter R^2 is the fraction of the variation of data explained by the linear model. R^2 > 0.8 for all replicates and genotypes indicates linear relationship of the colony expansion dynamic. Statistical significance was calculated with the t-test, the experiment was performed with 5 biological replicates. Statistical analysis using Student's t-test tested whether the mean expansion rate statistically differs between genotypes for the same treatment, where H0: means of expansion rates of *Psa* NZ13 and ΔTn*6212* for given conditions are equal and H1: means of expansion rates of *Psa* NZ13 and ΔTn*6212* for given conditions are not equal.
